# Modular Medical Imaging Agents Based on Azide–Alkyne Huisgen Cycloadditions: Synthesis and Pre‐Clinical Evaluation of ^18^F‐Labeled PSMA‐Tracers for Prostate Cancer Imaging

**DOI:** 10.1002/chem.202001795

**Published:** 2020-07-21

**Authors:** Verena I. Böhmer, Wiktor Szymanski, Keimpe‐Oeds van den Berg, Chantal Mulder, Piermichele Kobauri, Hugo Helbert, Dion van der Born, Friederike Reeβing, Anja Huizing, Marten Klopstra, Douwe F. Samplonius, Ines F. Antunes, Jürgen W. A. Sijbesma, Gert Luurtsema, Wijnand Helfrich, Ton J. Visser, Ben L. Feringa, Philip H. Elsinga

**Affiliations:** ^1^ Department of Nuclear Medicine and Molecular Imaging Department of Radiology Department of Surgical Oncology University of Groningen University Medical Center Groningen Hanzeplein 1 9713 GZ Groningen The Netherlands; ^2^ Stratingh Institute for Chemistry University of Groningen Nijenborgh 4 9747 AF Groningen The Netherlands; ^3^ FutureChemistry Toernooiveld 100 6525 EC Nijmegen The Netherlands; ^4^ Syncom Kadijk 3 9747 AT Groningen The Netherlands

**Keywords:** cancer, click chemistry, cycloadditions, imaging agents, positron emission tomography

## Abstract

Since the seminal contribution of Rolf Huisgen to develop the [3+2] cycloaddition of 1,3‐dipolar compounds, its azide–alkyne variant has established itself as the key step in numerous organic syntheses and bioorthogonal processes in materials science and chemical biology. In the present study, the copper(I)‐catalyzed azide–alkyne cycloaddition was applied for the development of a modular molecular platform for medical imaging of the prostate‐specific membrane antigen (PSMA), using positron emission tomography. This process is shown from molecular design, through synthesis automation and *in vitro* studies, all the way to pre‐clinical *in vivo* evaluation of fluorine‐18‐ labeled PSMA‐targeting ‘F‐PSMA‐MIC’ radiotracers (t_1/2_=109.7 min). Pre‐clinical data indicate that the modular PSMA‐scaffold has similar binding affinity and imaging properties to the clinically used [^68^Ga]PSMA‐11. Furthermore, we demonstrated that targeting the arene‐binding in PSMA, facilitated through the [3+2]cycloaddition, can improve binding affinity, which was rationalized by molecular modeling. The here presented PSMA‐binding scaffold potentially facilitates easy coupling to other medical imaging moieties, enabling future developments of new modular imaging agents.

## Graphical abstract

**Medical imaging–click it**! A versatile, Huisgen [3+2] cycloaddition‐based platform for prostate cancer diagnostics is introduced. The modular nature of this approach allows for attachment of different imaging probes and facile optimisation of binding potency to the Prostate‐Specific Membrane Antigen (PSMA).

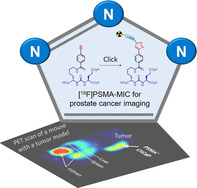

## Introduction

**Figure 1:**
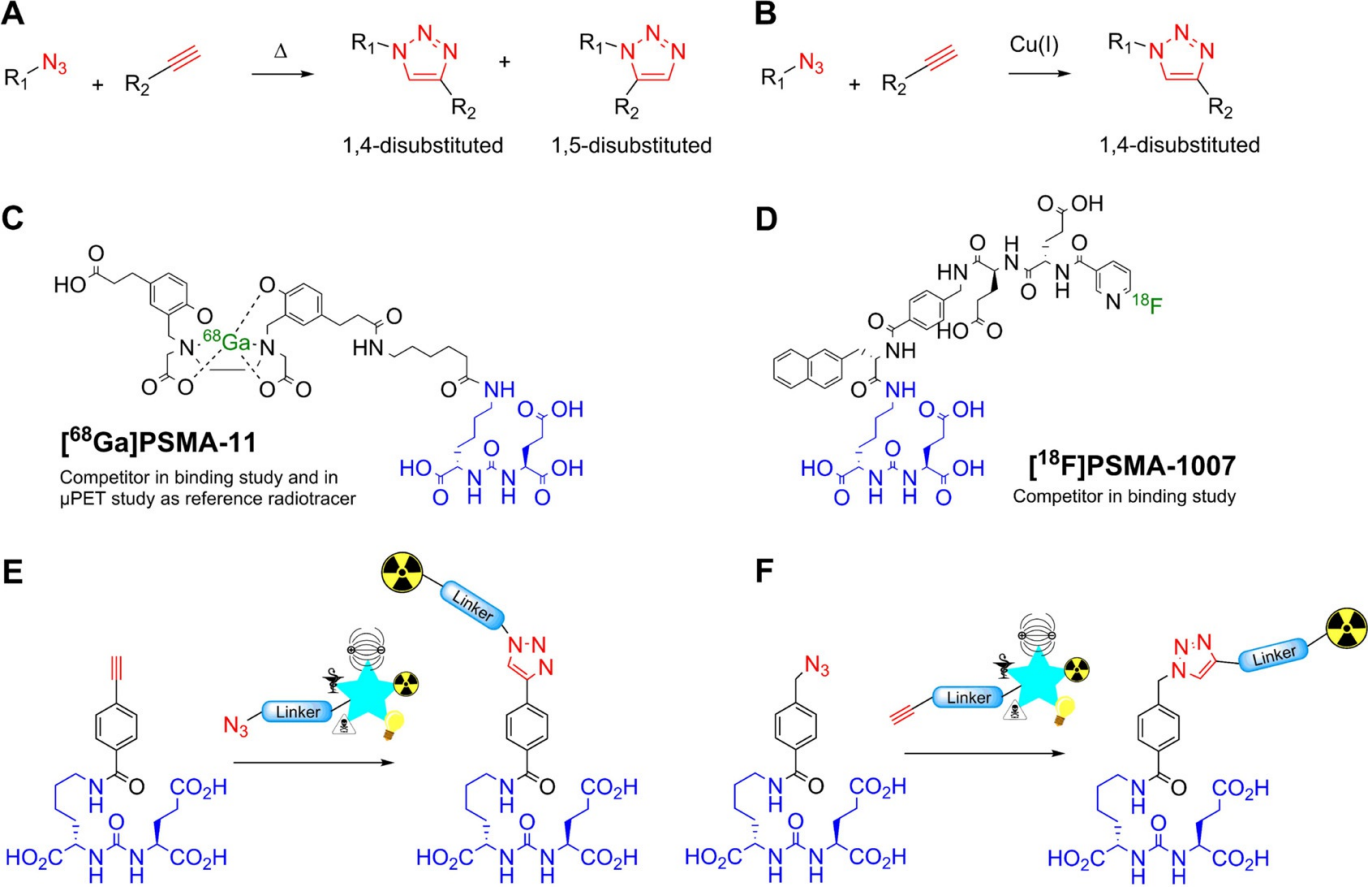
Overview of the [3+2] cycloadditions, clinically used prostate cancer radiotracers and the molecular platforms presented in this study. (A) Thermal azide–alkyne Huisgen [3+2] cycloaddition.^4^ (B) The copper(I)‐catalyzed azide–alkyne cycloaddition (CuAAC).^4^ (C) Structure of [^68^Ga]PSMA‐11 with the chelator HBED‐CC and the glutamate‐urea‐lysine (Glu‐urea‐Lys) motif (highlighted in blue) that binds to the prostate‐specific membrane antigen (PSMA).^50^ (D) Structure of [^18^F]PSMA‐1007.^48^ (E) Principle of a modular imaging agent consisting an alkyne‐functionalized Glu‐urea‐Lys motif that can be ‘clicked’ to a selected signaling moiety with azide‐functionality. The signaling moiety is chosen out of the range of different moieties, represented as the star, that is required for the aimed medical imaging application. The here presented study is showcasing its application in PET imaging. (F) The same principle of modular imaging agents using an azide‐functionalized Glu‐urea‐Lys motif^52^ to cover various suitable functionalized medical imaging moieties.

The accelerating pace of modern science frequently depends on breakthrough discoveries that reveal their true impact only decades later, as is evident for the azide–alkyne 1,3‐dipolarcycloaddition that revolutionized syntheses ranging from materials science to chemical biology. Recent progress in bioconjugations *in vitro*, bioorthogonal chemistry, *in vivo* transformations and medical imaging, among others, has revealed a key role for the azide–alkyne cycloaddition. Although reactions of 1,3‐dipolar compounds, such as ozones, nitrones or azides, were already known at the time, it was Rolf Huisgen who changed the face of heterocyclic chemistry by introducing the principle of [3+2]cycloadditions using 1,3‐dipolar compounds,^1^, ^2^ in particular the reaction of azides and alkynes providing 1,4‐ and 1,5‐ disubstituted 1,2,3‐triazoles (Figure 1 A).^3^, ^4^ With the introduction of the ‘click chemistry’ concept by Kolb, Finn and Sharpless in 2001, the azide–alkyne [3+2] cycloaddition was crowned to be the ‘cream of the crop’.^5^ Inspired by Huisgen's seminal work, Sharpless and Meldal discovered the regioselective, Cu^I^‐catalyzed azide–alkyne cycloaddition (CuAAC) variant (Figure 1 B).^4^, ^6^ Ever since, the Huisgen azide–alkyne cycloaddition is known to be the prototypical click chemistry method: it is a highly selective reaction, is performed under mild conditions, and proceeds with high yield while maximizing atom economy.^5^, ^7^ The resulting 1,2,3‐triazole scaffold showed to have biological activities^6^, ^8^ and was identified to be a bioisostere for esters,^9^ aromatic rings, double bonds, and amides.^10^ Therefore, compounds bearing this motif are widely applied in medicinal chemistry,^11^, ^12^ whereas click chemistry inspired the development of *in vivo* applications, such as the Staudinger‐Bertozzi ligation^13^ and the copper‐free, strain‐promoted click reaction (SPAAC).^14^ The fastest bioorthogonal reaction known at this moment is the inverse‐electron demand Diels–Alder of tetrazines with cyclooctenes.^15^

Gradually, CuAAC reactions were also used in clinics for the production of imaging agents, which enable the non‐invasive diagnosis through various modalities including magnetic resonance imaging (MRI),^16^, ^17^ optical imaging^18^ and positron emission tomography (PET).^19^, ^20^ Additionally, these imaging techniques were combined to obtain anatomical accuracy and associated physiological information, such as in the case of PET‐MRI imaging.^21^ The applied imaging agents are designed to unveil specific biomarkers that are targeted by ligands, such as small molecules, antibodies, affibodies or peptides,^22^ and visualized with a signaling moiety, for example, a complex of paramagnetic metal, fluorescent moiety or a radionuclide.^23^, ^24^

Click reactions are ideal reactions for syntheses of imaging agents, since they are highly specific and they do not require protection‐deprotection steps,^25^ which simplifies purification and further down‐stream processing. The up to 10^7^‐fold higher reaction speed of CuAAC compared to the thermal Huisgen [3+2] cycloaddition^26^ is particularly attractive for the synthesis of radiotracers,^27^ which is time‐sensitive due to short half‐lives of PET‐radionuclides (^11^C: 20.4 min, ^18^F: 109.7 min, and ^68^Ga: 67.9 min) that form the foundation of PET imaging due to their main decay mechanism of β^+^ decay ( >99 % for ^11^C, 96.7 % for ^18^F, 88.6 % for ^68^Ga).^19^, ^27^, ^28^ Since its first PET‐application in 2006,^29^ CuAAC found several applications in radiotracer preparation,^30^, ^31^, ^32^ the triazole appending‐agents (e.g. TAAG prosthetic group) and multivalent or multimodal imaging agents.^33^, ^34^, ^35^

Facing the challenges to develop new molecular scaffolds to be used as modular imaging agents for a broader range of medical applications, we explore azide–alkyne cycloadditions for quick assembly of imaging agents. Our key challenge is to develop a flexible synthetic platform to access imaging agents that are modular with respect to imaging modality and to the degree of multivalency. Here we present a CuAAC‐based radiotracer targeting prostate cancer (PCa), including automated synthesis, molecular modeling, *in vitro* studies and data obtained all the way to the *in vivo* evaluation in mice to showcase its potential for a clinically relevant disease.

PCa is the third most frequently diagnosed cancer among the male European population in 2018.^36^ The high morbidity constitutes a world‐wide health problem.^37^, ^38^, ^39^, ^40^ The current detection is based on the determination of prostate specific antigen (PSA) levels in blood, a digital rectal exam, and biopsies.^41^ However, the varying etiopathology of PCa makes it difficult to define the correct critical limit of PSA‐levels.^39^ For efficient diagnosis, a PCa‐specific non‐invasive diagnosis supported by medical imaging was urgently needed. In the 90’s, the discovery of the prostate‐specific membrane antigen (PSMA), overexpressed in PCa, improved the clinical assessment of PCa by nuclear medicine imaging.^39^, ^42^, ^43^, ^44^ Next to the presence in primary tumors, PSMA is expressed in metastases and primary lymph nodes, as well as in the recurrent disease.^45^, ^46^, ^47^ Hence, three PSMA‐targeting tracers have been clinically introduced for this purpose: [^68^Ga]PSMA‐11, [^18^F]PSMA‐1007 and [^18^F]DCFPyL.^48^, ^49^ They all are using the glutamate‐urea‐lysine (Glu‐urea‐Lys) binding motif (Figure 1 C and D).^50^ Realizing that this small motif binds specifically and with high affinity to PSMA and lends itself to further modifications, we envisioned that it provides a privileged scaffold for the development of click‐based PSMA‐targeted imaging agents.^51^ This was further supported by the key observation that a 1,2,3‐triazole attached to an oxyethylene‐linker compels PSMA to rearrange by molecular interactions and leads to improved binding.^51^

In the present study, we introduce a versatile, CuAAC‐based modular molecular platform for development of PSMA‐targeting imaging agents. In particular, we present a novel, fluorine‐18 based PSMA‐targeting radiotracer designated [^18^F]PSMA‐MIC01. To reduce radiation burden for the radiochemist and allow a robust and reproducible synthesis, [^18^F]PSMA‐MIC01 production was automated in a FlowSafe radiosynthesis module (see Supporting Information for more detail), which combines ^18^F‐fluorination in continuous‐flow microfluidics with a versatile CuAAC reaction performed in‐batch mode. After synthesis, optimization and characterization in terms of radiotracer stability, lipophilicity and *in vitro* binding affinity, the imaging potential of [^18^F]PSMA‐MIC01 was evaluated *in vivo* and compared to [^68^Ga]PSMA‐11. Additionally, aiming to increase the binding affinity, a second generation of click‐based PSMA‐targeting radiotracers was developed based on computational design, by introducing an additional aromatic ring in the side chain. Due to the ability to engage in the Huisgen [3+2] cycloaddition, the PSMA‐binding scaffold presented here can potentially be easily modified for other medical imaging modalities (Figure 1 E and F).

## Results and Discussion

### Design of F‐PSMA‐MIC01

**Figure 2:**
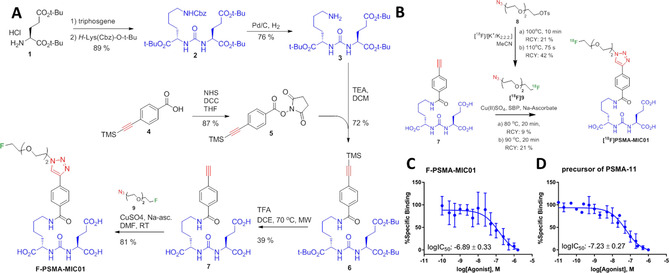
Synthesis and binding affinity of F‐PSMA‐MIC01. (A) Synthesis route of the alkyne‐Glu‐urea‐Lys motif and the reference compound F‐PSMA‐MIC01. (B) Radiolabeling towards radiotracer [^18^F]‐PSMA‐MIC01. a) Manual synthesis route of [^18^F]PSMA‐MIC01. The final radiotracer was obtained in an overall radiochemical yield of 9 % in a total production time of 148 min, including purification of intermediate and product. b) The automated synthesis route using the FlowSafe radiosynthesis module. (C–D) logIC_50_ determination of the F‐PSMA‐MIC01 (C) and the precursor of [^68^Ga]PSMA‐11 (D) using the cell‐based competitive binding radioassay with [^68^Ga]PSMA‐11 as competitor on the PSMA‐positive LNCaP cell line. Mean values ± SD (*n*=3).

PSMA is a well‐characterized target in structure–activity‐relationship (SAR) studies.^53^ The natural function of this membrane zinc‐metallopeptidase is to cleave glutamate from *N*‐acetyl‐l‐aspartyl‐l‐glutamate. This antigen has a glutamate‐favoring S1’‐pocket^54^, ^55^, ^56^ and SAR analysis revealed an adaptive, hydrophobic‐favoring S1‐pocket, created by an arginine patch formed by Arg463, Arg534 and Arg536 that can accommodate a variety of inhibitors.^57^ PSMA‐targeting compounds with the Glu‐urea‐Lys motif bind to the S1‐hydrophobic pocket and the S1’‐pocket, as well as to the zinc ions.^57^ Interestingly, it was found that the presence of a 1,2,3‐triazole motif in PSMA inhibitors enables binding to an additional arene‐binding site, which has inspired us to use this moiety in developing PSMA‐targeting radiotracers with high affinity.^57^ For this purpose, we designed a modular synthesis approach for PSMA‐targeting radiotracers, which can potentially be applied to different imaging modalities, by adapting the existing Glu‐urea‐Lys motif^57^ so that it is able to undergo the Huisgen [3+2] cycloaddition. We introduce the radiotracer [^18^F]PSMA‐MIC01 (Figure 2 A), which is formed by the alkyne‐Glu‐urea‐Lys motif and PET‐radionuclide ^18^F, spaced from the 1,2,3‐triazole by a diethylene‐glycol‐linker, which was shown to display the right linker length.^51^

### Synthesis of precursors and F‐PSMA‐MIC01

The synthesis of amine‐Glu‐urea‐Lys motif **3** was performed as previously described.^58^, ^59^, ^60^ The alkyne‐functionality was introduced by NHS‐ester coupling to 4‐[(trimethylsilyl)ethynyl] benzoic acid **4**, followed by reaction with amine **3**. Deprotection with trifluoroacetic acid gave alkyne‐Glu‐urea‐Lys motif **7** (Figure 2 A). The fluorinated azide‐reference **9** was obtained in 33 % yield by substitution reaction of tosylate **8** using tetrabutylammonium fluoride (see Supporting Information for experimental details). CuAAC of precursor **9** with alkyne‐Glu‐urea‐Lys motif **7** gave the compound F‐PSMA‐MIC01 in 81 % yield (Figure 2 A).

### Radiolabelling of [^18^F]PSMA‐MIC01

With a radiochemical yield (RCY)^61^ of 21 %, the purified intermediate [^18^F]**9** was used for the CuAAC reaction with **7**. Subsequently, the crude reaction mixture was purified by semi‐preparative HPLC and formulated into a 5 mL injectable solution of 10 % EtOH in phosphate‐buffered saline (PBS). [^18^F]PSMA‐MIC01 was manually produced in an overall RCY of 9 % with an overall production time of 148 min (Figure 2 B).

Clinical translation requires higher amounts of radioactivity than those manually achievable, which are limited by radiation burden for the radiochemist. Therefore, the synthesis of [^18^F]PSMA‐MIC01 was automated on a FlowSafe radiosynthesis module, a continuous‐flow microfluidics platform (see Supporting Information for details). [^18^F]PSMA‐MIC01 was produced in an overall RCY of 21 % with an overall production time of 139 min (see Supporting Information for experimental details). The higher RCY can be explained by the use of the microfluidic set up for the [^18^F]fluorination towards intermediate [^18^F]**9**. Microfluidic systems have a higher surface‐to‐volume ratio which results in an increased heat transfer capacity compared to in‐batch syntheses.^62^ This enabled reduction of the effective reaction time of the [^18^F]fluorination to 75 s with concomitant reduction of ^18^F‐side‐products and increased the intermediary RCY of [^18^F]**9** to 42 % and overall RCY to 21 %. The obtained molar activity of [^18^F]PSMA‐MIC01 (A_M_: 14.1±12 GBq μmol^−1^) and high radiochemical purity (see Supporting Information for UPLC chromatogram) was sufficient for evaluation of the *in vivo* organ distribution (*vide infra*). The A_M_ can be increased by increasing the starting amount of ^18^F, which would improve the binding potency of the tracer due to less competition. The stability of the radiotracer [^18^F]PSMA‐MIC01 in 10 % EtOH/PBS was tested for 4 h with radio‐HPLC. No degradation products could be detected (chromatogram shown in the Supporting Information), indicating that the radiotracer is stable. The measured lipophilicity (log*D*) in *n*‐octanol/PBS was −3.01±0.22 (see Supporting Information). It has been indicated in literature that for the detection of primary PCa and lymph node metastasis, a log*D* value between −2 and −3 is ideal.^63^ The here obtained log*D* is therefore in this ideal range.

### *In vitro* studies of F‐PSMA‐MIC01

The binding affinity of F‐PSMA‐MIC01 to PSMA was determined in a cell‐based competitive binding radioassay using [^68^Ga]PSMA‐11 (Figure 1 C) and the reference compound F‐PSMA‐MIC01 as competitor on PSMA‐expressing LNCaP cells.^64^ As expected, we discovered that F‐PSMA‐MIC01 was able to block the binding of [^68^Ga]PSMA‐11 and had a binding affinity in the nanomolar range, as shown in Figure 2 C. To compare the binding affinity of F‐PSMA‐MIC01 with „gold standard“ PSMA‐tracers, the same assay was performed using the precursor of [^68^Ga]PSMA‐11 (Figure 2 D). To our delight, the obtained logIC_50_ values for F‐PSMA‐MIC01 and the precursor of [^68^Ga]PSMA‐11 showed the same high inhibitory potency.

### *In vivo* studies of [^18^F]PSMA‐MIC01

**Figure 3:**
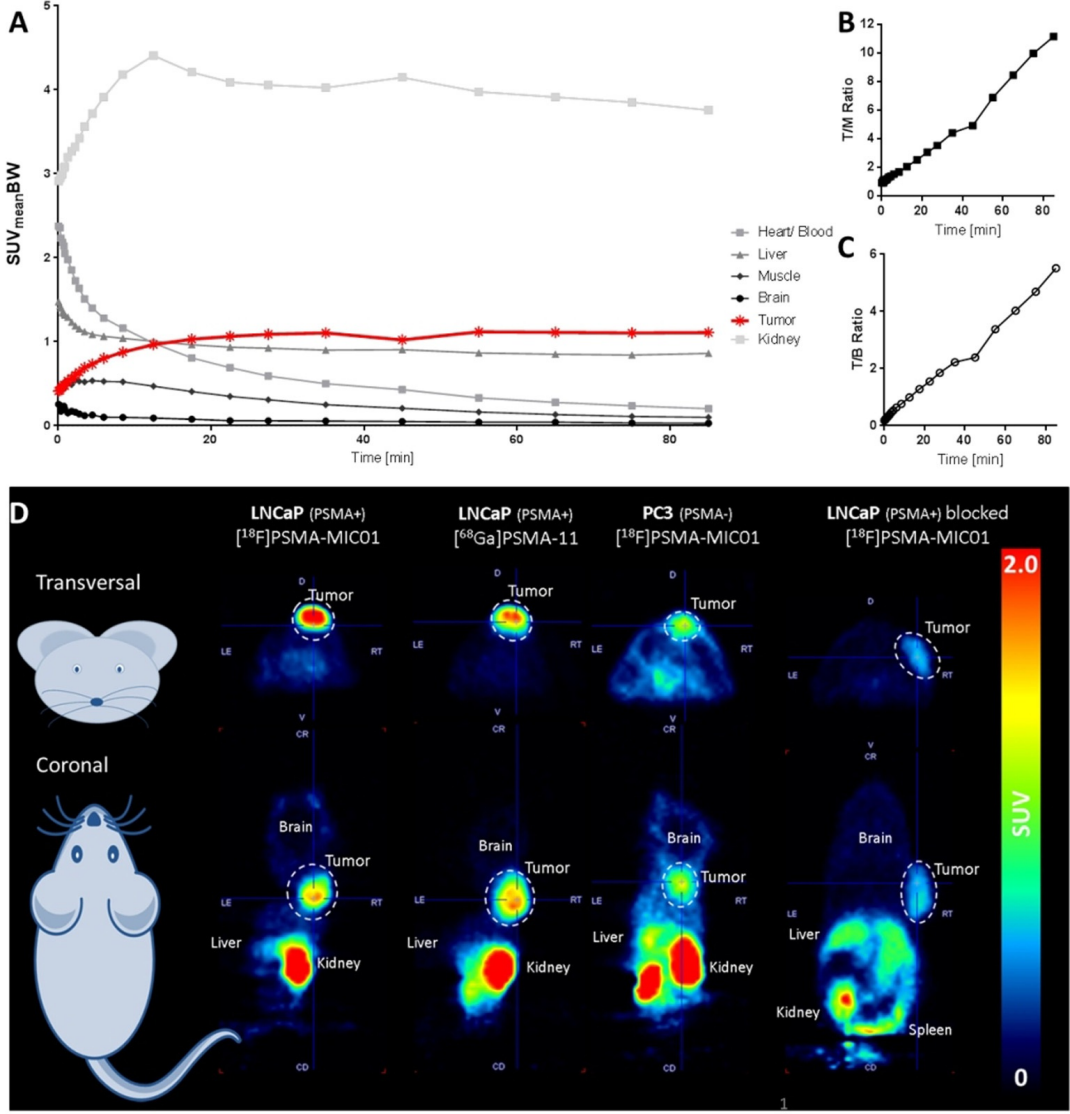
Organ distribution of [^18^F]PSMA‐MIC01 in a murine model. (A) Time‐activity curves in several organs during a 90 min dynamic PET scan, calculated based on the body‐weight corrected Standardized Uptake Value (SUV_meanBW_). The values are represented as Mean (*n*=6). SD is removed for readability (for complete graphs, see Supporting Information). (B) Tumor‐to‐muscle (T/M) ratio. (C) Tumor‐to‐blood (T/B) ratio. (D) Representative PET images obtained during a 30 min static PET scan, started 60 min p.i. The dotted lines highlight the tumors (LNCaP‐ or PC3‐ xenografts). The first two scans shown, [^68^Ga]PSMA‐11 and [^18^F]PSMA‐MIC01, are performed in the same animals on consecutive days. The upper row shows the transversal view on mouse and the lower row the coronal view.

The *in vivo* imaging potential of [^18^F]PSMA‐MIC01 was evaluated using a murine animal model (see Supporting Information for experimental details).^65^ This was performed in a procedure that involved the study of the tumor uptake, binding specificity and comparison to [^68^Ga]PSMA‐11. Tumor uptake of [^18^F]PSMA‐MIC01 was assessed by performing a 90 min dynamic PET scan. The time‐activity curves (TAC, Figure 3 A) represent the radiotracer kinetics of [^18^F]PSMA‐MIC01, calculated by image quantification using the Standardized Uptake Values (SUV_meanBW_).^66^ The TACs reveal that, after 20 min, the uptake in the PSMA‐positive LNCaP tumor is increased compared to heart/blood, liver, muscle and brain. This is also supported by the increasing tumor‐to‐blood (T/B) and the tumor‐to‐muscle (T/M) ratios (Figure 3 B and C).

After successful demonstration of the tumor uptake of [^18^F]PSMA‐MIC01, binding specificity to PSMA was evaluated and compared to [^68^Ga]PSMA‐11. For this purpose, three experimental groups were defined: i) Comparison of tumor uptake in LNCaP xenografts of [^18^F]PSMA‐MIC01 and [^68^Ga]PSMA‐11 in the same animal. ii) A negative‐control tumor model, in which a PSMA‐negative xenograft is used based on the PC3 cell line,^64^ to check whether the observed tumor uptake is caused by specific interactions with PSMA or rather based on non‐specific effects, such as the enhanced permeability and retention (EPR) effect.^67^ iii) Confirmation of binding specificity of radiotracer [^18^F]PSMA‐MIC01, by blocking PSMA in LNCaP‐xenografts prior to radiotracer injection,^65^ using the potent PSMA‐inhibitor 2‐(phosphonomethyl)pentanedioic acid (2‐PMPA, IC_50_: 0.3 nm 68). All groups were evaluated by visual assessment of the PET image and the percentage injected dose per gram (%ID g^−1^).

The PET images (Figure 3 D) visualize the organ distribution of [^18^F]PSMA‐MIC01 in different groups. In all four conditions, tumor uptake was detected. Although the tumor uptake based on visual assessment of the SUV‐based PET image of [^18^F]PSMA‐MIC01 and [^68^Ga]PSMA‐11 looks quite similar, the uptake in the PC3‐ and blocked LNCaP‐xenografts is clearly reduced. This is in agreement with the *ex vivo* organ distribution of [^18^F]PSMA‐MIC01, shown in Table 1, in which parts of the organs were dissected after the PET scan and the radioactivity content was measured. The tumor uptake of [^68^Ga]PSMA‐11 was 6.8±6.3 %ID g^−1^, whereas the uptake of [^18^F]PSMA‐MIC01 was 11.8±4.2 %ID g^−1^ in LNCaP xenografts. Although [^18^F]PSMA‐MIC01 showed equivalent uptake compared to [^68^Ga]PSMA‐11 in terms of the probability value, the Cohen's d (*d*=0.93, see Supporting Information for calculation) indicates even a large effect size between these two groups. In literature, the LNCaP tumor uptake of [^18^F]PSMA‐1007 is reported to be 8.04±2.4 %ID g^−1^,^65^ which is in the same range than the values obtained in this study for [^68^Ga]PSMA‐11 and [^18^F]PSMA‐MIC01. For non‐specific binding of [^18^F]PSMA‐MIC01 in the PSMA‐negative PC3 xenograft, an uptake value of 3.0±1.8 %ID g^−1^ was measured. Compared to the LNCaP‐xenografts, this is significantly lower and indicates only minor non‐specific binding effects. In the blocking group, we observed tumor uptake of 2.8±0.8 %ID g^−1^, which is a similar to the PSMA‐negative PC3 xenograft.

**Table 1:** *Ex vivo* organ distribution of the radiotracers [^18^F]PSMA‐MIC01 and [^68^Ga]PSMA‐11, radioactivity was corrected for the injected dose per gram (%ID g^−1^).

	**LNCaP** (PSMA+) [^18^F]PSMA‐MIC01	**LNCaP** (PSMA+) [^68^Ga]PSMA‐11	**PC3** (PSMA‐) [^18^F]PSMA‐MIC01	**LNCaP** (PSMA+) blocked [^18^F]PSMA‐MIC01
**tumor**	**11.7±4.2**	**6.8±6.3**	**3.0±1.7**	**2.8±0.8**
whole blood	1.6±1.3	2.2±3.8	3.4±1.8	1.8±0.6
plasma	0.9±5.2	1.0±0.5	6.0±3.5	3.8±1.3
urine	314±420	45.4±30.8	184±260	644±627
heart	0.6±0.4	0.2±0.0	1.0±0.6	0.7±0.5
lungs	1.3±0.5	1.1±0.4	2.1±1.1	1.1±0.3
spleen	5.8±3.4	15.9±7.3	3.1±1.4	1.0±0.2
liver	5.6±1.3	0.2±0.3	9.4±2.9	5.7±1.4
stomach	0.6±0.2	0.4±0.2	1.2±0.6	7.3±16.4
kidney	42.0±9.0	69.1±21.1	39.8±28.8	28.5±20.7
muscles	0.5±0.2	0.2±0.1	0.6±0.3	0.3±0.1
small intestine	1.6±2.1	0.5±0.6	1.3±0.6	1.2±1.5
large intestine	1.4±1.5	0.7±0.9	1.4±0.5	0.9±0.3
pancreas	0.8±0.7	0.6±0.6	0.8±0.3	0.5±0.2
bone	0.2±0.1	0.1±0.1	0.5±0.2	0.3±0.1
brain	0.1±0.0	0.0±0.0	0.2±0.1	0.1±0.0
salivary glands	0.5±0.3	0.9±0.4	1.1±0.6	1.0±0.8

The values are represented as Mean±SD %D g^−1^. (n=6 mice for [^18^F]PSMA‐MIC01 on LNCaP‐xenografts, n=5 mice for [^68^Ga]PSMA‐11 and [^18^F]PSMA‐MIC01 on PC3‐xenograft).

[^68^Ga]PSMA‐11 and other PSMA‐binding tracers are known to have a quite high accumulation in the salivary glands of patients^69^ which is a limiting factor in its application as theranostic agent due to the possible side‐effect of xerostomia.^70^ The *ex vivo* organ distribution data show that the salivary gland uptake is low in all groups (0.5 to 1.1 %ID g^−1^). In summary, the *in vivo* data suggest that the tracer uptake of [^18^F]PSMA‐MIC01 is comparable with [^68^Ga]PSMA‐11.

### Design of 2^nd^ generation F‐PSMA‐MIC compounds

**Figure 4:**
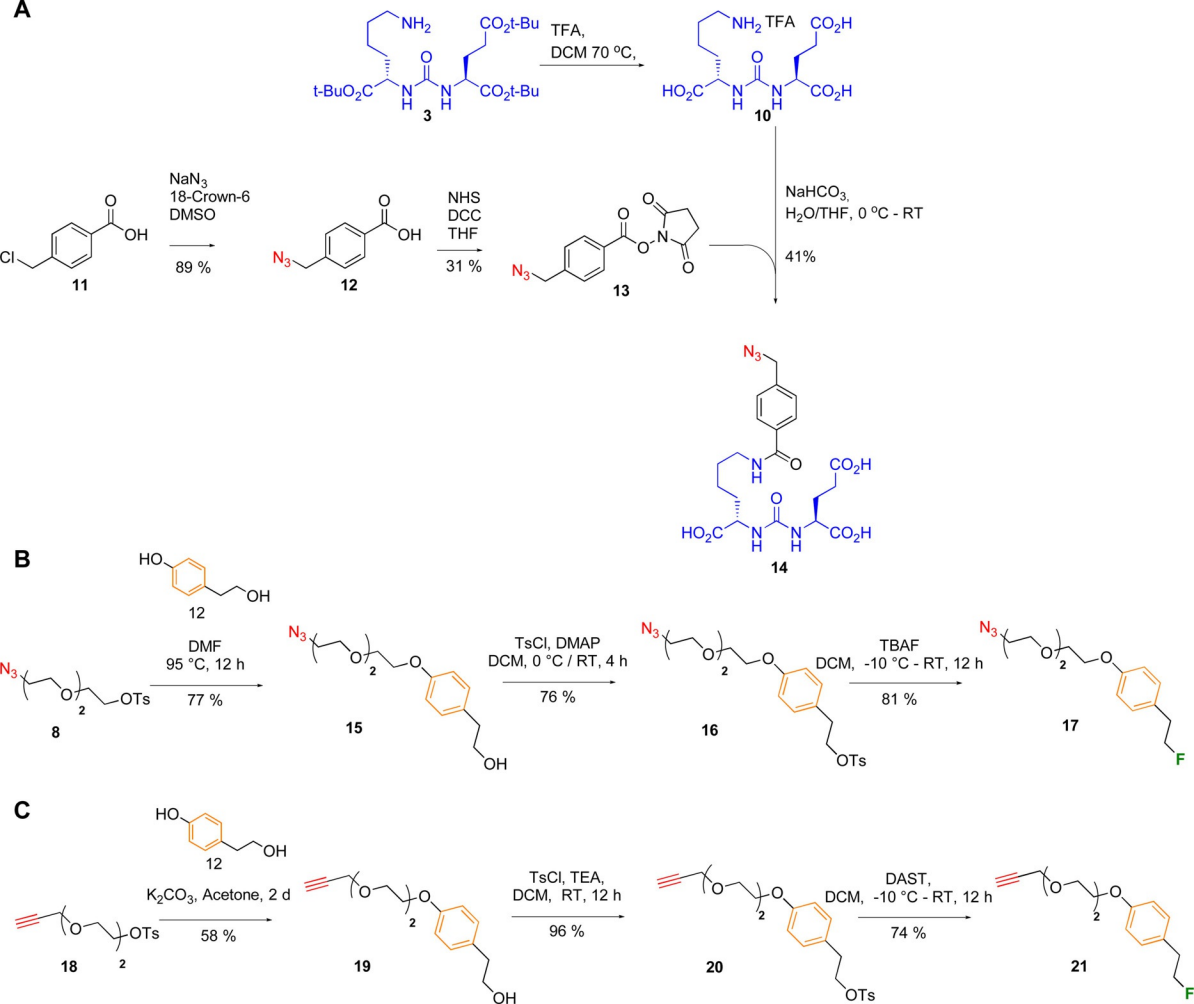
Overview of the compounds used for the 2^nd^ generation F‐PSMA‐MIC compounds.

Encouraged by the good imaging performance of [^18^F]PSMA‐MIC01, we explored the application of CuAAC to introduce structural changes that further improve the binding of [^18^F]PSMA‐MIC01 towards PSMA. It is known that the incorporation of 1,2,3‐triazole and polyethylene‐glycol linkers in PSMA‐targeting compounds induces a rotation of Trp541 towards Arg511,^51^ thus opening the arene‐binding cleft and precluding the closure of the entrance lid. It was shown that the combination of a 1,2,3‐triazole, di‐ or tetra‐ethylene‐glycol linker and a dinitro‐phenyl group resulted in increase of the binding affinity.^51^ Based on this observation, we designed a second generation of tracers, F‐PSMA‐MIC02−F‐PSMA‐MIC04, for PET imaging purposes (Figure 4). Their design was aimed at studying the effect of the following modifications: i) the arrangement of the triazole group, by functionalizing the PSMA‐binding scaffold with both alkyne‐ (F‐PSMA‐MIC01 and F‐PSMA‐MIC02) and azide‐motifs (F‐PSMA‐MIC03 and F‐PSMA‐MIC04); ii) the introduction of an additional aromatic ring to target the arene‐binding site in F‐PSMA‐MIC02 and F‐PSMA‐MIC‐04. To avoid challenging nucleophilic substitutions on electron‐rich aromatics,^71^ it was decided to add another ethylene‐linker between the benzene ring and the ^18^F‐radionuclide. With this design, all compounds could be radiolabeled by the same procedure, using a tosylate moiety as leaving group.

### Synthesis of 2^nd^ generation F‐PSMA‐MIC compounds

Whereas the synthesis of F‐PSMA‐MIC01 employed alkyne‐Glu‐urea‐Lys motif **7**, the design of molecules F‐PSMA‐MIC03 and F‐PSMA‐MIC04 required the preparation of the previously reported azide analogue **14** (Figure 4).^52^ To this end, compound **3** was first deprotected and coupled to activated 4‐azidomethyl benzoic acid **13** in a yield of 41 % (Figure 4 A) (see Supporting Information for experimental details). Azide‐ and alkyne‐precursors **8** and **18** were modified with 4‐(2‐hydroxyethyl)phenol **12** to introduce the benzene‐ring, and were fluorinated using tetrabutylammonium fluoride or diethylaminosulfur trifluoride (DAST) in a yield of 81 % for azide‐precursor **17** and 74 % for alkyne‐precursor **21**. F‐PSMA‐MIC02, F‐PSMA‐MIC03 and F‐PSMA‐MIC04 were obtained in CuAAC reaction in yields of 33 %, 43 % and 9 %, respectively (see Supporting Information for experimental details).

### Molecular modeling studies of F‐PSMA‐MIC compounds

**Figure 5:**
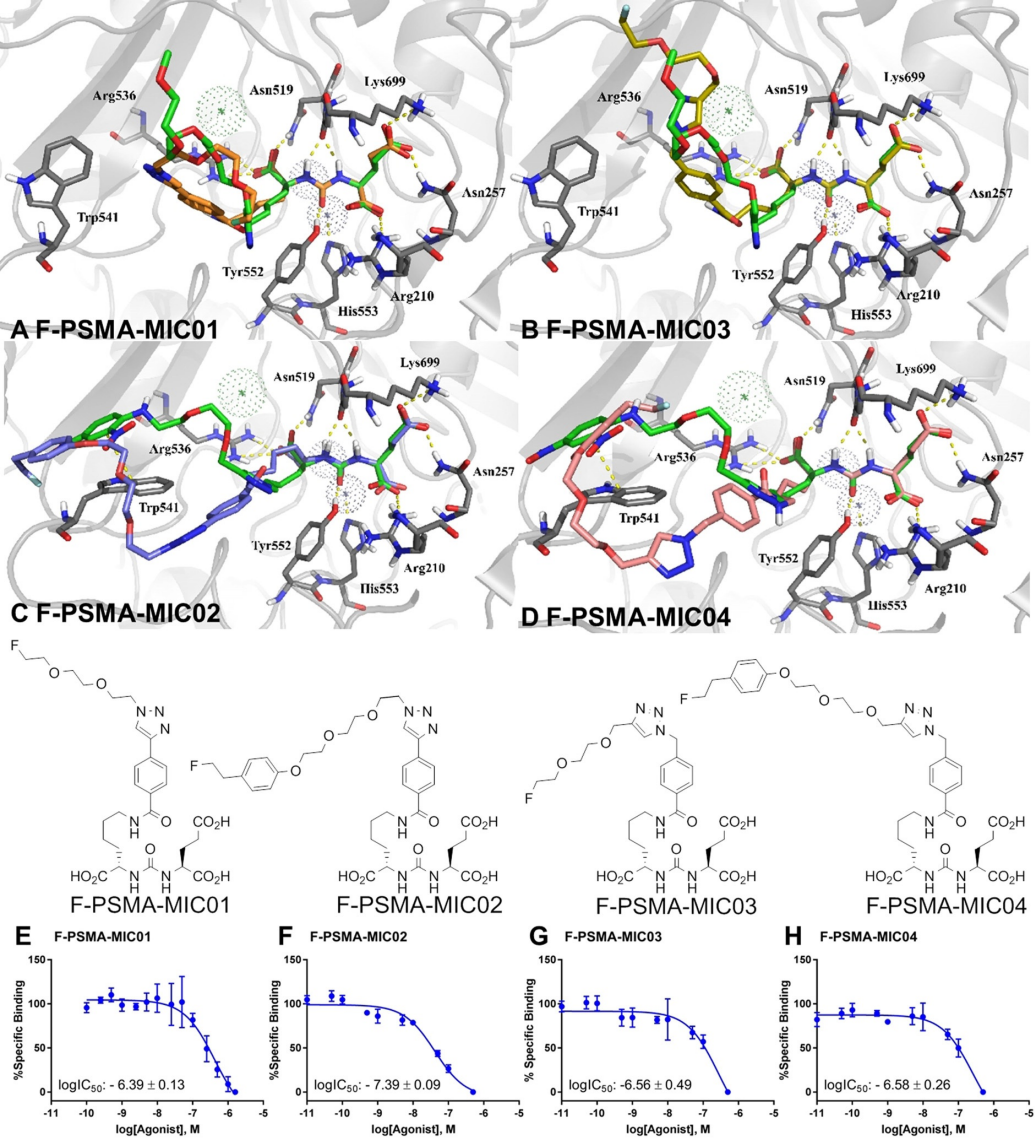
Molecular docking studies and binding affinities of the 2 ^nd^ generation F‐PSMA‐MIC compounds. A–D: Molecular docking poses. (A) F‐PSMA‐MIC01 (orange) and (B) F‐PSMA‐MIC03 (yellow), superimposed on the binding mode of MeO‐P4 with PSMA (PDB ID: 2XEJ); (C) F‐PSMA‐MIC02 (purple) and (D) F‐PSMA‐MIC04 (pink), superimposed on the binding mode of ARM‐P2 with PSMA (PDB ID: 2XEI). Protein is represented as grey cartoon with key residues in sticks, co‐crystallized ligands in green, metal ions as dotted spheres. Hydrogen bonds and π−π stackings are depicted as yellow dashed lines. (E–H) LogIC_50_ determination. Mean values ± SD (E,F and H: *n*=3, G: *n*=4). Competitive binding radioassays of the F‐PSMA‐MIC compounds on LNCaP cells using [^18^F]PSMA‐1007 as radioactive competitor.

**Figure 6:**
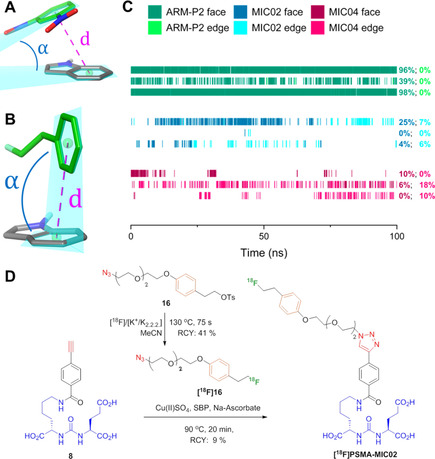
Analysis of the π−π stacking of Trp541 and the additional aromatic ring in F‐PSMA‐MIC02 and F‐PSMA‐MIC04 and the radiolabeling of the strongest binder in this study. (A) Example of a face‐to‐face π−π stacking between dinitrophenyl (DNP, green) and Trp541 (gray) from the complex of ARM‐P2 with PSMA (PDB ID: 2XEI). (B) Example of an edge‐to‐face π−π interaction between the additional electron‐rich ring (green) and Trp541 (gray) from the second MD run of F‐PSMA‐MIC04 (frame number 282). The ring distance and ring angle measurements are illustrated as pink dotted lines and blue arcs, respectively. In all the structures, carbon atoms are colored as indicated above, and other atoms are colored blue (nitrogen), red (oxygen) and light green (fluorine). (C) Timeline representation of the π−π interactions in the three MD runs of ARM‐P2 (green), F‐PSMA‐MIC02 (blue) and F‐PSMA‐MIC04 (red). Dark colors indicate face‐to‐face interactions and bright colors indicate edge‐to‐face interactions. On the right side, the frequency of the interactions for individual runs is reported with the same coloring. (D) The automated synthesis route of [^18^F]PSMA‐MIC02 using the FlowSafe radiosynthesis module.

The influence of the structural modifications on the binding towards PSMA was first evaluated in a molecular docking study using previously reported crystal structures.^51^ Crystal structures of PSMA with the Glu‐urea‐Lys motif coupled by a 1,2,3‐triazole either to methoxy tetra‐ethylene glycol linker (MeO‐P4) or to a dinitrophenyl di‐ethylene glycol linker (ARM‐P2) were used, to include the two distinct conformations of Trp54.^51^ This key residue is flipped when no interaction is occurring at the remote arene‐binding site^51^ (Figure 5 A and B), whereas it is flat when a stabilizing π−π interaction is formed (Figure 5 C and D). All the inhibitors show similar docking poses to the parent compounds, MeO‐P4 and ARM‐P2. The Glu‐urea‐Lys motifs of all inhibitors interact with the protein active site residues Arg210, Asn257, Tyr552, Lys553, Lys699, Asn519 and Arg536. For F‐PSMA‐MIC01 and F‐PSMA‐MIC03, the diethylene glycol‐linker is not involved in specific interactions, as it can be expected due to its large flexibility. On the other hand, F‐PSMA‐MIC02 and F‐PSMA‐MIC04 target the arene‐binding site and engage in a π−π interaction with Trp541 as ARM‐P2, albeit with suboptimal ring orientations. To assess the evolution and the stability of this interaction, molecular dynamics (MD) simulations were performed on the crystal structure of ARM‐P2 and the docked conformations of F‐PSMA‐MIC02 and F‐PSMA‐MIC04 (Figure 6). Three 100 ns long MD simulations were carried out for each compound (see Supporting Information for computational details).

ARM‐P2 features an electron‐deficient ring designed to interact with the electron‐rich indole moiety of Trp541. In MD simulations, we were able to reproduce this face‐to‐face π−π stacking that was remarkably stable over the course of the simulations (Figure 6 C). Examining molecules F‐PSMA‐MIC02 and F‐PSMA‐MIC04, which for reasons of synthetic accessibility featured an electron‐rich ring, revealed that this interaction is present, albeit intermittent and at intervals is of an edge‐to‐face nature (Figure 6 A and B), which is consistent with the electrostatic view of the π−π interaction of two electron‐rich aromatics.^72^ This electron‐rich aromatic ring also forms cation–π interactions with Arg511 in the arene‐binding site (see Supporting Information).

Overall, molecular modeling suggests that π−π contacts with PSMA are enabled by the addition of an aromatic ring and contribute to the binding affinity. However, the docking simulations were not able to discriminate between the two different arrangements of the triazole group in compounds F‐PSMA‐MIC01/MIC‐02 and F‐PSMA‐MIC03/MIC04.

### *In vitro* studies of the 2^nd^ generation F‐PSMA‐MIC compounds

During the pre‐clinical evaluation of [^18^F]PSMA‐MIC01, many hospitals including the University Medical Center Groningen changed from using [^68^Ga]PSMA‐11 to [^18^F]PSMA‐1007. Therefore, the binding affinities for the 2^nd^ generation PSMA‐tracers‐tracers were determined in a radioassay using [^18^F]PSMA‐1007 as radioactive competitor (Figure 1 D).

To determine the influence of the structural changes introduced in the 2^nd^ generation F‐PSMA‐MIC compounds, we first evaluated the arrangement of triazole‐ring by comparing F‐PSMA‐MIC01 with F‐PSMA‐MIC03, yet we observed no significant difference. However, in the case of targeting the arene‐binding site (F‐PSMA‐MIC02 and F‐PSMA‐MIC04), the rigid triazole‐benzene part of F‐PSMA‐MIC02 gives a lower logIC_50_ value, representing a higher binding affinity towards PSMA. Binding affinities of the second generation PSMA‐tracers showed that F‐PSMA‐MIC02 has a higher binding affinity than F‐PSMA‐MIC01. The positive influence of a hydrophobic, rigid linker attached to the lysine part was already reported earlier.^73^ This suggests that the strongest PSMA binding affinity of F‐PSMA‐MIC02 is due to the rigid triazole‐benzene part and as the affinity observed for this compound was the highest, we proceeded to radiolabel [^18^F]PSMA‐MIC02 and fully automate its synthesis.

### Radiolabeling of the 2^nd^ generation radiotracer [^18^F]PSMA‐MIC02

The manual synthesis showed good conversion towards [^18^F]PSMA‐MIC02 and the procedure was implemented and optimized on the FlowSafe radiosynthesis module in an overall RCY of 9 %, yielding a 5 mL injectable solution of 10 % EtOH in PBS with an overall production time of 169 min. The obtained log*D* value for [^18^F]PSMA‐MIC02 is −3.22±0.10 and its stability was tested for 4 h in 10 % EtOH/PBS (see HPLC chromatograms in the Supporting Information). The log*D* value of [^18^F]PSMA‐MIC02 was slightly higher than the log*D* of [^18^F]PSMA‐MIC01.

## Conclusions and Outlook

We have established a flexible molecular platform showcasing its potential for the development of prostate cancer imaging agents based on the Cu^I^‐catalyzed Huisgen [3+2] cycloaddition and showed the successful route from molecular design all the way to *in vivo* evaluation. Pre‐clinical analysis of [^18^F]PSMA‐MIC01 revealed similar imaging performance as compared to the clinically used [^68^Ga]PSMA‐11 radiotracer. Importantly, the binding potential of the Glu‐urea‐Lys motif was maintained, offering prospects for the use of clickable alkyne‐PSMA‐binding motif **7** as a general modular platform.

Further investigation of the clickable PSMA‐scaffold **7** led to the design of a second generation of F‐PSMA‐MIC compounds. Molecular docking and dynamic studies were conducted to analyze the interaction of these compounds with PSMA. The *in vitro* data indicate that targeting the arene‐binding site only partly improves binding affinity due to the electron‐rich aromatic introduced to target the arene‐binding site. The alkyne‐modified PSMA‐scaffold revealed a robust and reproducible binding affinity towards PSMA and is a useful scaffold for ‘clicking’ to imaging agents that enable other modalities, such as chelators or fluorescent dyes or to increase the (multi)valency. This modular click‐based strategy would be applicable for other molecular targets as well. It also demonstrates how fundamental discoveries in heterocyclic synthesis, as achieved by Huisgen and colleagues, ultimately provides major perspectives for early detection of life‐threatening diseases.

## Conflict of interest

FutureChemistry and Syncom were commercial partners in this project

## Supporting information

As a service to our authors and readers, this journal provides supporting information supplied by the authors. Such materials are peer reviewed and may be re‐organized for online delivery, but are not copy‐edited or typeset. Technical support issues arising from supporting information (other than missing files) should be addressed to the authors.

Supplementary
