# Supplementary material for: Modular Medical Imaging Agents Based on Azide–Alkyne Huisgen Cycloadditions: Synthesis and Pre‐Clinical Evaluation of 18F‐Labeled PSMA‐Tracers for Prostate Cancer Imaging
Source: Chemistry. 2020 Jul 21;26(47):10871–81. doi: 10.1002/chem.202001795 (PMC7496508; doi:10.1002/chem.202001795)
Supplement: Supplementary file 1 — Supplementary [file CHEM-26-10871-s001.pdf]

Chemistry–A European Journal

Supporting Information

Modular Medical Imaging Agents Based on Azide–Alkyne Huisgen Cycloadditions: Synthesis and Pre-Clinical Evaluation of ^{18}F-Labeled PSMA-Tracers for Prostate Cancer Imaging

Verena I. Böhmer,[a, b] Wiktor Szymanski,*[a, b] Keimpe-Oeds van den Berg,[a] Chantal Mulder,[a] Piermichele Kobauri,[b] Hugo Helbert,[a, b] Dion van der Born,[c] Friederike Reeßing,[a, b] Anja Huizing,[a, b] Marten Klopstra,[d] Douwe F. Samplonius,[a] Ines F. Antunes,[a] Jürgen W. A. Sijbesma,[a] Gert Luurtsema,[a] Wijnand Helfrich,[a] Ton J. Visser,[d] Ben L. Feringa,*[b] and Philip H. Elsinga*[a]

Table of Contents

Experimental Section 3

1. General Materials. 3
2. Organic Chemistry 4
   2.1. Synthesis of F-PSMA-MIC01 4
   2.2. Synthesis of F-PSMA-MIC02 11
   2.3. Synthesis of F-PSMA-MIC03 13
   2.4. Synthesis of F-PSMA-MIC04 22
3. Radiochemistry 24
   3.1. Fluorine-18 Production and Preparation. 24
4. Radiotracer Stability of [^{18}F]PSMA-MIC01 and [^{18}F]PSMA-MIC02 28
5. Distribution Coefficient Log*D* 30
6. Cell Culture. 30
   6.1. Cell Binding Studies. 31
7. Animal Study. 32
   7.1. *In vivo* Study. 32
   7.2. Organ Distribution and Metabolite Analysis. 32
8. Computational Details 33
9. References 59

NMR and Mass Spectra 60

   10. Synthesis of F-PSMA-MIC01 60
   11. Synthesis of F-PSMA-MIC02 64
   13. Synthesis of F-PSMA-MIC03 66
   14. Synthesis of F-PSMA-MIC04 69

Experimental Section

1. General Materials.

Solvents and reagents were purchased from commercial suppliers FluoroChem, TCI Chemicals, Rathburn, Sigma-Aldrich, Acros chemicals, Fluka, Merck, Honeywell and Braun. Column chromatography was performed using Merck silica gel 60 Å (40-63 μm). ^{1}H-NMR (500 MHz) were measured on a Bruker Avance 4-channel NMR Spectrometer. ^{1}H-NMR (400 MHz) and ^{19}F-NMR were measured on an Agilent Technologies 400-MR (400/54 Premium Shielded) Spectrometer (400 MHz). NMR spectra were analyzed with the Software MestReNova (Mestrelab Research) and chemical shifts are expressed in ppm with residual chloroform (δ = 7.26 ppm (^{1}H)), methanol (δ = 3.35 ppm (^{1}H)), or dimethylsulfoxide (δ = 2.77 ppm (^{1}H)) as reference. In case not stated otherwise, radio-thin layer chromatography (rTLC) and thin layer chromatography (TLC) were conducted with Sigma-Aldrich silica gel on TLC Al foils with fluorescent indicator 254 nm and measured with an Amersham Typhoon GE Healthcare Bio-Sciences AB Fluorescent analyzer or Cyclone phosphor storage system from PerkinElmer Life and Analytical Science, Waltham, USA. High Performance Liquid Chromatography (HPLC) was performed on a preparative HPLC system composed of a Waters Pump Control Module II, XBridge Prep C18 5μm 10x250mm column, Waters 2489 UV/Visible Detector, Berthold FlowStar LB 513 radioactivity.

FlowSafe radiosynthesis module was developed and programmed by FutureChemistry. The FlowSafe radiosynthesis module is a synthesizer in which radiochemical reactions can be automated. It is a continuous-flow microfluidic platform using a glass microreactors (100 uL), which are connected to a back-pressure regulator. The regulator adjusts the pressure within the microreactor to 5.0 bar, which increases the boiling points of solvent. Additionally, this module can combine microfluidics with in-batch reactions, as well as purification, by solid-phase-extraction or HPLC.

[^{18}O]H_2O was purchased from Cortecnec. For γ-counter measurements the Wizard 2480 from Perkin Elmer was used. PET image analysis and quantification was performed using PMOD v3.9 software (PMOD Technologies, Zürich, Switzerland).

The docking calculations were performed on a HP EliteDesk, with an Intel Core i7-6700 processor with four cores and an NVIDIA GeForce GTX 1060 3GB graphics card, using Schrödinger Release 2019-4, Maestro 12.2.[1] The molecular dynamics (MD) and the quantum chemistry (QM) calculations were performed with the Desmond and Jaguar modules on the Peregrine cluster at the University of Groningen. The docking images were

obtained with Pymol 2.2.3, and the MD analysis graphs were obtained through the Simulation Interactions Diagram tool in Maestro.

2. Organic Chemistry

2.1. Synthesis of F-PSMA-MIC01

2.1.1. (9*S*,13*S*)-Tri-tert-butyl 3,11-dioxo-1-phenyl-2-oxa-4,10,12-triazapentadecane-9,13,15-tricarboxylate (2).

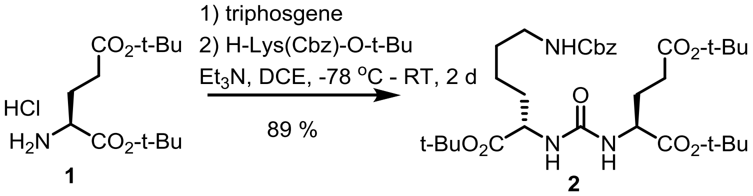

L-glutamic acid di-*tert*-butyl ester hydrochloride **1** (10.0 g, 34 mmol, 1.7 eq.) and triethylamine (Et_3N, 15.4 mL, 111.0 mmol) were dissolved in dichloroethane (300 mL) and the resulting solution was cooled to -78 °C. Triphosgene (3.41 g, 11.5 mmol, 0.6 eq.) in dichloroethane (100 mL) was added dropwise to the reaction mixture. Upon complete addition, the reaction was allowed to warm to room temperature and stir for 30 min. H-Lys(Z)-O-t-Bu hydrochloride (7.55 g, 20.2 mmol) was added, followed by Et_3N (2.8 mL, 20.2 mmol, 1.0 eq.). The reaction mixture was allowed to stir at room temperature over the weekend. The reaction can be followed on TLC by means of cerium nitrate dip reagent (with heating). The reaction mixture was then diluted with dichloroethane (500 mL), and washed with water (2 x 500 mL). The crude mixture was dried over sodium sulfate (Na_2SO_4) and concentrated under reduced pressure. A clear oil (16.4 g) was isolated. Column chromatography of the resulting oil (silica gel, hexane : ethyl acetate (EtOAc) gradient) yielded the target compound **2** as a colorless oil (11.2 g, 18.0 mmol, 89 %). ^1H NMR (299 MHz, Chloroform-*d*) δ 7.40 – 7.28 (m, 5H), 5.22 – 5.00 (m, 6H), 4.33 (d, *J* = 4.6 Hz, 2H), 2.41 – 2.19 (m, 4H), 2.19 – 1.98 (m, 2H), 1.98 – 1.71 (m, 4H), 1.45 (s, 18H), 1.43 (s, 9H), and is in agreement with literature data.[2]

2.1.2. Di-tert-butyl (((*S*)-6-amino-1-(tert-butoxy)-1-oxohexan-2-yl)carbamoyl)-L-glutamate (3).

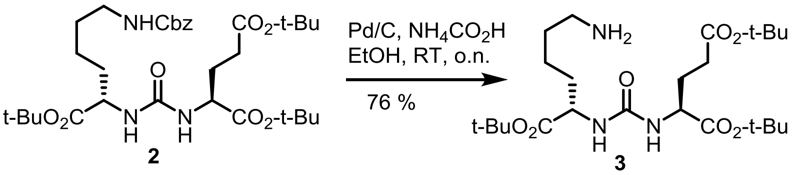

To a solution of compound **2** (11.17 g, 17.96 mmol) in ethanol (EtOH, 360 mL) were added ammonium formate (11.33 g, 179.6 mmol, 10.0 eq.), followed by 10 % palladium on carbon (10 % Pd/C, 1.13 g). The suspension was stirred at room temperature overnight. The reaction mixture was filtered over Celite and concentrated to give 9.23 g oil, which solidified to a white residue. The product, which still contained ammonium formate, was dissolved in

dichloromethane (DCM, 100 mL), filtered, and washed with 50 mL water. The layers were separated by centrifuging (4700 rpm, 20 min). The organic layer was washed with 20 mL brine, dried over Na_2SO_4, filtered, and concentrated to give compound **3** (6.62 g, 13.6 mmol, 76 %) as a white foam with a purity of 99.8 % according to ELSD-HPLC. ^{1}H NMR (300 MHz, Chloroform-*d*) δ 6.34 (d, *J* = 7.9 Hz, 1H), 6.12 (d, *J* = 8.2 Hz, 1H), 4.39 – 4.22 (m, 2H), 3.10 (m, 2H), 2.34 (m, 2H), 2.06 (d, *J* = 7.0 Hz, 1H), 1.79 (dq, *J* = 21.8, 6.3 Hz, 5H), 1.59 (s, 4H), 1.45 (s, 18H), 1.43 (s, 9H). HPLC-MS: 3.963 min purity 99.8% (ELSD), ES-MS *m/z* 488.2 [M+1] and is in agreement with literature data.[2]

2.1.3. 2,5-Dioxopyrrolidin-1-yl 4-((trimethylsilyl)ethynyl)benzoate (5).

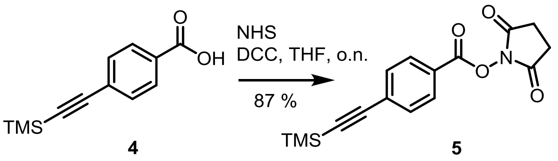

To 4-[(trimethylsilyl)ethynyl] benzoic acid **4** (500 mg, 2.29 mmol) and *N*-hydroxysuccinimide (NHS, 264 mg, 2.29 mmol) in tetrahydrofuran (THF, 18 mL) was added *N,N'*-dicyclohexylcarbodiimide (DCC, 473 mg, 2.29 mmol). The mixture was stirred under nitrogen overnight. After 10 min a suspension started to form. The reaction mixture was filtered over Celite and the Celite cake was washed with THF. The filtrate was concentrated to give 725 mg crude product. The product was purified by automated column chromatography (silica gel, heptane : EtOAc gradient) to give compound **5** (0.63 g, 2.00 mmol, 87 %) as a white solid, which was used in the next step without further characterization. ^{1}H NMR (299 MHz, DMSO-d_6) δ 8.09 – 8.01 (m, 2H), 7.72 – 7.65 (m, 2H), 2.88 (s, 4H), 0.25 (s, 9H).

2.1.4. Di-tert-butyl (((*S*)-1-(tert-butoxy)-1-oxo-6-(4-((trimethylsilyl)ethynyl)benzamido) hexan-2-yl)carbamoyl)-L-glutamate (6).

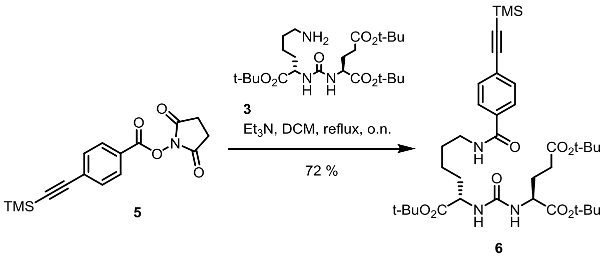

A mixture of compound **3** (0.50 g, 1.03 mmol), succinimide **5** (0.32 g, 1.03 mmol, 1.0 eq.) and Et_3N (0.14 mL, 1.0 mmol, 1.0 eq.) in 50 mL DCM was stirred at reflux temperature under nitrogen overnight. The mixture was washed with 50 mL water, dried over Na_2SO_4, filtered and concentrated to give 0.82 g yellow oil. The crude product was purified by automated column chromatography (silica gel, gradient heptane : EtOAc) to give compound **6** (500 mg, 0.727 mmol, 72 %) as a white foam with a purity of 89 % according to HPLC, which was used in the next step without further characterization. ^{1}H NMR (299 MHz, Chloroform-*d*) δ 8.35 (s, 1H), 7.78 (d, *J* = 8.0 Hz, 2H), 7.48 (d, *J* = 8.0 Hz, 2H), 6.75 (s, 2H), 5.22 (s, 2H), 4.29 (d, *J* = 22.5 Hz, 2H), 3.44 (s, 2H),

2.33 (tq, J = 17.1, 10.5, 8.6 Hz, 2H), 2.07 (m, 2H), 1.84 (q, J = 8.1, 7.3 Hz, 4H), 1.45 (s, 9H), 1.44 (s, 9H), 1.43 (s, 9H), 0.26 (s, 9H). ES-MS *m/z* 688.3 [M+1].

2.1.5. (((*S*)-1-Carboxy-5-(4-ethynylbenzamido)pentyl)carbamoyl)-L-glutamic acid (7).

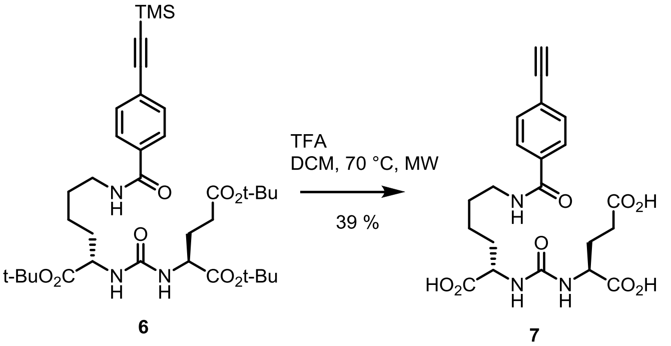

Compound **6** (1.3 g, 1.89 mmol) was stirred in dry dichloroethane (5 mL) and trifluoroacetic acid (TFA, 10 mL) at room temperature for 3 h. The reaction mixture was worked up by evaporation and co-evaporation with dichloroethane three times to remove residual TFA. The compound was purified by automated reverse phase column chromatography. Fractions containing the product were combined and partially evaporated. The aqueous residue was dried by freeze drying. The product **7** was isolated as a white solid (580 mg, 1.3 mmol, 69 %). ^{1}H NMR (299 MHz, Methanol-d_4) δ 8.50 (d, J = 5.9 Hz, 1H), 7.85 – 7.71 (m, 2H), 7.59 – 7.46 (m, 2H), 4.29 (ddd, J = 8.2, 6.5, 4.9 Hz, 2H), 3.65 (s, 1H), 3.38 (tt, J = 6.4, 3.3 Hz, 2H), 2.50 – 2.31 (m, 2H), 2.25 – 2.05 (m, 2H), 2.04 – 1.79 (m, 2H), 1.79 – 1.59 (m, 4H), 1.49 (p, J = 7.3 Hz, 2H). ES-MS *m/z* 448.2 [M+1], 917.2 [2M+23].

2.1.6. 2-(2-(2-azidoethoxy)ethoxy)ethoxy)ethyl (8).

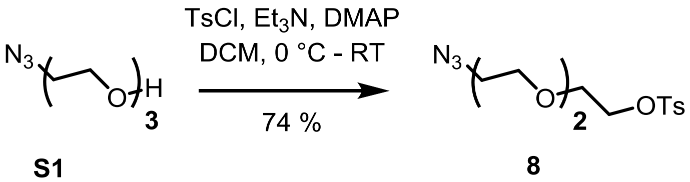

2-(2-(2-azidoethoxy)ethoxy)ethan-1-ol **S1** (1.0 g, 5.7 mmol) was dissolved in 6 mL anhydrous DCM with Et_3N (1.5 g, 14.9 mmol) and 4-dimethylaminopyridine (DMAP, 0.09 g, 0.74 mmol) and cooled to 0 °C. *p*-Toluenesulfonyl chloride (TsCl, 1.5 g, 8.0 mmol) was dissolved in 3 mL DCM and slowly added to the solution. The reaction mixture was stirred at room temperature for 18 h. After completion, the reaction mixture was washed with 1 M hydrochloric acid (aq. 1 M HCl), saturated sodium bicarbonate (sat. $NaHCO_3$) and brine. The organic layer was separated and volatiles were removed *in vacuo* to obtained crude product **8** was purified by column chromatography (silica gel, 1:2 EtOAc: hexane). The product **8** was obtained as yellow oil (1.4 g, 4.3 mmol, 74 %). ^{1}H NMR (400 MHz, Chloroform-*d*) δ= 7.80 (d, J = 8.2 Hz, 2H), 7.36 – 7.33 (m, 2H), 4.18 – 4.15 (m, 2H), 3.72 – 3.69 (m, 2H), 3.64 (dd, J = 5.5, 4.6 Hz, 2H), 3.60 (s, 4H), 3.38 – 3.35 (m, 2H), 2.45 (s, 3H), 1.57 (s, 3H), which is in agreement with literature data.[3]

2.1.7. 1-Azido-2-(2-(2-fluoroethoxy)ethoxy)ethane (9).

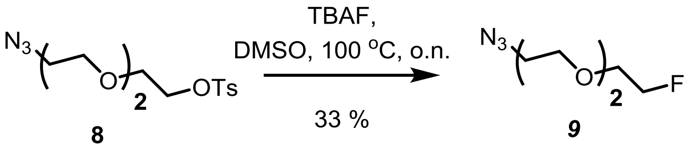

To a solution of compound **8** (200 mg, 0.60 mmol) in *tert*-butanol (4.8 mL) was added tetrabutylammonium fluoride (TBAF, 1 M in THF, 1.2 mL, 1.2 mmol, 2.0 eq.). The mixture was stirred in a closed vial at 100 °C under nitrogen overnight. The reaction mixture was concentrated and the residue was extracted with DCM (5 mL) / water (5 mL). The organic layer was dried over Na_2SO_4, filtered and concentrated to give 267 mg yellow oil. The crude product was purified by column (silica gel, heptane : EtOAc, gradient 5 % - 10 % EtOAc) to give compound **9** as a colorless oil (62 mg, 0.20 mmol, 33 %). ^{1}H NMR (299 MHz, Chloroform-*d*) δ 4.69 – 4.62 (m, 1H), 4.52 – 4.46 (m, 1H), 3.85 – 3.78 (m, 1H), 3.75 – 3.64 (m, 7H), 3.40 (t, *J* = 5.1 Hz, 2H), which is in agreement with literature data.[3]

2.1.8. (((*S*)-1-Carboxy-5-(4-(1-(2-(2-(2-fluoroethoxy)ethoxy)ethyl)-1H-1,2,3-triazol-4-yl)benzamido)pentyl)carbamoyl)-L-glutamic acid (F-PSMA-MIC01).

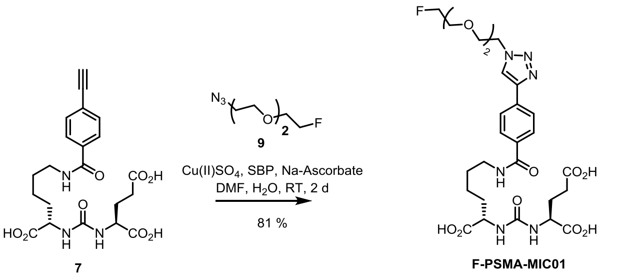

A mixture of compounds **7** (30 mg, 0.07 mmol) and **9** (21 mg, 0.067 mmol, 1.0 eq.) in dimethylformamide (DMF, 1.5 mL) was stirred under nitrogen. A sonicated yellow suspension of copper(II) sulfate pentahydrate (CuSO$_4$ · 5 H_2O, 0.83 mg, 0.03 mmol, 0.05 eq.) and L-ascorbic acid sodium salt (1.3 mg, 0.007 mmol, 0.1 eq.) in water (0.5 mL) was added. The resulting yellow solution was stirred for 2 d. A colorless reaction mixture was formed. The mixture was concentrated and purified by preparative HPLC to give reference compound **F-PSMA-MIC01** as a white solid (34 mg, 0.054 mmol, 81 %). ^{1}H NMR (299 MHz, Methanol-d_4) δ 8.47 (s, 1H), 7.92 (app d, *J* = 1.2 Hz, 4H), 4.66 (t, *J* = 5.0 Hz, 2H), 4.60 – 4.51 (m, 1H), 4.44 – 4.34 (m, 1H), 4.19 (s, 2H), 4.02 – 3.91 (m, 2H), 3.77 – 3.70 (m, 2H), 3.69 – 3.56 (m, 4H), 3.40 (t, *J* = 6.7 Hz, 2H), 2.38 (s, 2H), 2.12 (s, 2H), 1.95 (s, 2H), 1.66 (d, *J* = 8.6 Hz, 4H), 1.51 (s, 2H). ^{13}C NMR (75 MHz, Methanol-d_4) δ 168.20, 146.36, 133.96, 133.36, 127.62, 125.10, 122.43, 83.75, 81.52, 70.26, 70.16 (d, *J* = 1.5 Hz), 70.05, 70.01, 68.91, 50.16, 39.61, 32.85, 29.28, 28.80, 22.81. ^{19}F NMR (376 MHz, Methanol-d_4) δ = -224.62 (tt, *J* = 48.3, 30.3). ES-MS *m/z* 625.3 [M+1]. ESI-HR-MS: m/z 647.2437 [M+Na] (theoretical: m/z 647.2447 [M+Na]).

2.2. Synthesis of F-PSMA-MIC02

2.2.1. 2-(4-(2-(2-(2-azidoethoxy)ethoxy)ethoxy)phenyl)-ethan-1-ol (15).

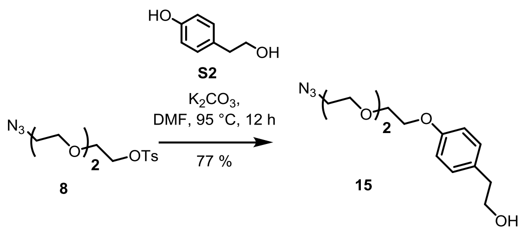

Potassium carbonate (1.19 g, 8.62 mmol, 2.0 eq.), 4-(2-hydroxyethyl)phenol **S2** (0.715 g, 5.17 mmol, 1.2 eq.) and **8** (1.42 g, 4.31 mmol, 1.0 eq.) were dissolved in 10 mL DMF. After completion, 50 mL of water and 20 mL DCM was added to the reaction mixture. The water layer was extracted three times with 20 mL DCM. The organic layers are combined and washed with aq. 1 M HCl, sat. $NaHCO_3$ and brine. The crude product was purified with column chromatography (silica gel, 3 % Methanol (MeOH) in DCM). **15** was obtained (0.974 mg, 3.3 mmol, 77 %). ^{1}H NMR(400 MHz, Chloroform-*d*) δ = 7.11 (d, *J* = 8.6 Hz, 2H), 6.87 (d, *J* = 11.6 Hz, 2H), 4.12 (t, *J* = 4.5, 3.5 Hz, 2H), 3.86 (t, *J* = 3.5, 4.7 Hz, 1H), 3.82 (t, *J* = 6.5 Hz, 2H), 3.76 – 3.72 (m, 2H), 3.68 (m, 2H), 3.38 (t, *J* = 5.1,3.8 Hz, 1H), 2.81 (t, *J* = 6.5 Hz, 2H).

2.2.2. 2-(4-(2-(2-(2-azidoethoxy)ethoxy)ethoxy)phenyl)ethyl-4-methylbenzene-1-sulfonate (16).

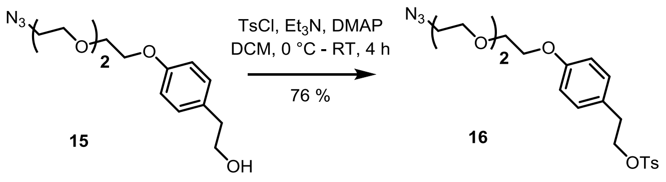

Compound **15** (0.1 g, 0.34 mmol, 1.0 eq.) was dissolved in 5 mL of anhydrous DCM and Et_3N (0.09 g, 0.89 mmol, 2.6 equiv.) was added to the solution. The solution was cooled to 0 °C using an ice bath under inert atmosphere. DMAP (2.89 mg, 0.0236 mmol, (0.05 eq.) was dissolved in 1 mL dry DCM and was added to the solution. TsCl (90.0 mg, 0.47 mmol, 1.4 equiv.) was dissolved in 3 mL anhydrous DCM and added slowly to the cooled reaction mixture. After addition the ice bath was removed and the reaction mixture is left to stir for 4 h at room temperature. After completion 50 mL of water and 50 mL of DCM was added to the reaction mixture. The organic layer was separated and the water layer was extracted with DCM (3x20 mL). The combined organic layer was washed with aq. 1 M HCl, sat. $NaHCO_3$ and brine, dried with magnesium sulfate ($MgSO_4$) and concentrated in vacuo. The obtained crude product was purified by column chromatography (silica gel, 1:2 EtOAc: hexane). The pure product **16** was obtained as a colourless oil (1.12 g, 2.49 mmol, 76 %). ^{1}H NMR (500 MHz, Chloroform-*d*) δ 7.69 (d, *J* = 6.4 Hz, 2H), 7.29 (d, *J* = 8.1 Hz, 2H), 7.01 (d, *J* = 8.5 Hz, 2H), 6.80 (d, *J* = 8.5 Hz, 2H), 4.16 (t, *J* = 7.1 Hz, 2H), 3.86 (t, *J* = 3.7,5.0 Hz, 2H), 3.74 (m, 2H), 3.70 - 3.67 (m, 4H), 3.38 (t, *J* = 5.1 Hz, 1H), 2.88 (t, *J* = 7.1 Hz, 1H), 2.43 (s, 2H). ESI-HR-MS: m/z 467.1965 [M+NH_4] (theoretical: m/z 467.1959 [M+NH_4])

2.2.3. 1-(2-(2-(2-azidoethoxy)Ethoxy)ethoxy)-4-(2-fluoroethyl)benzene (17).

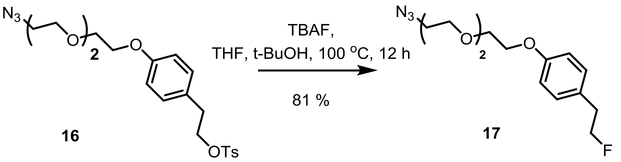

TBAF (1 M solution in THF, 1.14 mL, 1.14 mmol, 2.0 eq.) was added to a stirring solution of **16** (0.19 g, 0.57 mmol, 1.0 eq.) in *tert*-butanol (t-BuOH, 4.56 mL). The mixture was stirred for 12 h at 100 °C, and then over night at room temperature. The residue was dissolved in water and the crude product was extracted from the aqueous phase with DCM. The organic layer was dried over $NaSO_4$ and concentrated *in vacuo*. The crude product **17** was purified by column chromatography (25% EtOAc : hexane) and was obtained as a yellow oil (0.139, 0.47 mmol, 81 %). ^{1}H-NMR (500 MHz, Chloroform-*d*) δ 7.14 (d, *J* = 8.6 Hz, 2H), 6.87 (d, *J* = 8.6 Hz, 2H), 4.58 (dt, *J* = 47.1, 6.7 Hz, 2H), 4.12 (t, *J* = 4.6, 5.2 Hz, 2H), 3.86 (t, *J* = 5.2, 4.5 Hz, 2H), 3.74 (m, 2H), 3.71 – 3.66 (m, 4H), 3.39 (t, *J* = 5.1 Hz, 2H), 2.95 (dt, *J* = 22.8, 6.7 Hz, 2H). ESI-HR-MS: m/z 315.1831 [M+NH_4] (theoretical: m/z 315.1827 [M+NH_4])

2.2.4. 2-(4-(2-(2-(2-azidoethoxy)ethoxy)ethoxy)phenyl)ethyl-4-methylbenzene-1-sulfonate (F-PSMA-MIC02).

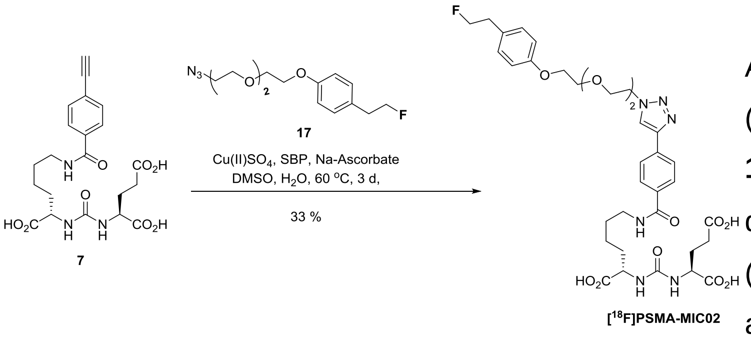

Alkyne-Glu-urea-Lys compound **7** (29.7 mg, 0.07 mmol) and tosylate **17** (27.6 mg, 0.1 mmol) were dissolved in 400 uL. $CuSO_4 \cdot 5\ H_2O$ (1.65 mg, 0.07 mmol), L-ascorbic acid sodium salt (2.62 mg, 0.1 mmol) and bathophenanthrolinedisulfonic acid disodium salt hydrate (SBP, 5.85 mg, 0.1 mmol) were dissolved in MilliQ water to obtain the click reagents in a green solution and added to the solution of **7** and **17** to obtain a reddish reaction mixture, which was heated up to 60 ^{o}C and left stirring over the weekend. The reaction was diluted in MilliQ water and purified by preparative HPLC and freeze-dried by **F-PSMA-MIC02** as a white, fluffy solid (16.5 mg, 0.02 mmol, 33 %). ^{1}H NMR (400 MHz, Methanol-*d₄*) δ 8.46 (s, 2H), 7.90 (s, 6H), 7.09 (d, *J* = 10 Hz, 3H), 6.77 (d, *J* = 10 Hz, 3H), 4.62 (t, *J* = 6.2 Hz, 3H), 4.52 (dt, *J* = 59.3, 8.1 Hz, 2H), 4.31 (s, 4H), 4.98 (t, *J* = 5.4 H, 6 Hz, 3H), 3.96 (t, *J* = 6.2 Hz, 3H), 3.74 (t, *J* = 5.8, 5.5 Hz, 3H), 3.66 (s, 6H), 3.42 (s, 3H), 2.87 (dt, *J* = 29.1, 8.2 Hz, 2H), 2.42 (m, 4H), 2.18 (m, 3H), 1.93 (m, 4H), 1.69 (m, 6H), 1.52 (m, 4H). Due to limited solubility we were unable to obtain ^{13}C NMR spectra in sufficient quality.^{19}F NMR (400 MHz, Methanol-*d_4*) δ -216. ESI-HR-MS: m/z 745.3200 [M+H] (theoretical: m/z 745.3203 [M+H])

2.3. Synthesis of F-PSMA-MIC03

2.3.1. (((*S*)-5-Amino-1-carboxypentyl)carbamoyl)-L-glutamic acid (10)

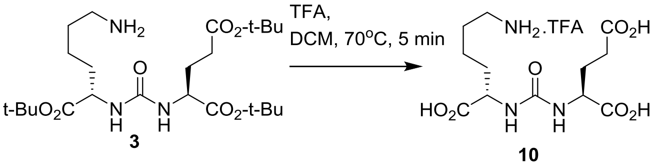

Compound **3** (666 mg, 1.37 mmol) was divided in two batches. Each batch was dissolved in 5 mL DCM and 10 mL TFA and stirred at 70 °C under microwave conditions for 5 min. The reaction mixture was concentrated *in vacuo* for 4.5 h (0.02 mbar, 50 °C) to give compound **10** as a hygroscopic, sticky, white solid (728 mg). ^{1}H NMR (300 MHz, Methanol-d_4) δ 4.37 – 4.24 (m, 2H), 2.92 (t, *J* = 7.5 Hz, 2H), 2.47 – 2.36 (m, 2H), 2.24 – 2.06 (m, 2H), 2.00 – 1.81 (m, 2H), 1.79 – 1.61 (m, 4H), 1.50 (d, *J* = 7.4 Hz, 2H), which is in agreement with literature data.[4] ^{19}F NMR (282 MHz, Methanol-d_4) δ -77.13. ES-MS *m/z* 319.7 [M+1].

2.3.2. 4-Azidomethyl benzoic acid (12).

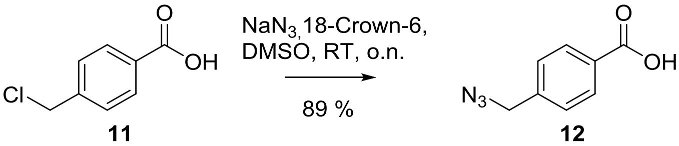

To a mixture of sodium azide (7.62 g, 117 mmol, 2.0 eq.) and 18-Crown-6 (1.17 mL, 5.86 mmol, 0.10 eq.) in dimethylsulfoxide (DMSO, 23 mL) was added 4-chloromethyl benzoic acid **11** (10.0 g, 58.6 mmol). The mixture was stirred at 25 °C overnight. The reaction mixture was poured into 200 mL EtOAc, washed with aq. 0.1 M HCl (2 × 200 mL) and brine (200 mL), dried over $NaSO_4$, filtered and concentrated to give azide **12** as a white solid (9.18 g, 51.8 mmol, 89 %). ^{1}H NMR (300 MHz, Chloroform-*d*) δ 8.24 – 8.03 (m, 2H), 7.44 (d, *J* = 7.9 Hz, 2H), 4.45 (s, 2H), in agreement with literature data.[5,6]

2.3.3. 2,5-Dioxopyrrolidin-1-yl 4-(azidomethyl)benzoate (13).

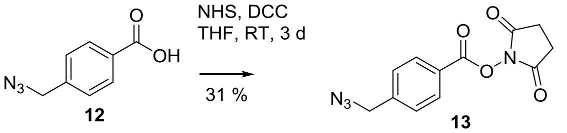

To a solution of acid **12** (3.00 g, 16.9 mmol) and NHS (1.95g, 16.9 mmol, 1.0 eq.) in THF (60 mL) was added DCC (3.50 g, 16.9 mmol, 1.0 eq.). The mixture was stirred overnight. Analysis by NMR overnight indicated 84% conversion. After stirring for another two nights the solids were filtered off and washed with THF. The filtrate was concentrated to give crude **13** as a white solid. Purification by column chromatography (silica gel, DCM : MeOH gradient) gave **13** as a white solid (1.42 g, 5.18 mmol, 31 %). ^{1}H NMR (299 MHz, Chloroform-*d*) δ 8.23 – 8.12 (m, 2H), 7.54 – 7.46 (m, 2H), 4.49 (s, 2H), 2.94 (s, 4H), in agreement with literature data.[6]

2.3.4. (((*S*)-5-(4-(azidomethyl)benzamido)-1-carboxypentyl)carbamoyl)-L-glutamic acid (14).

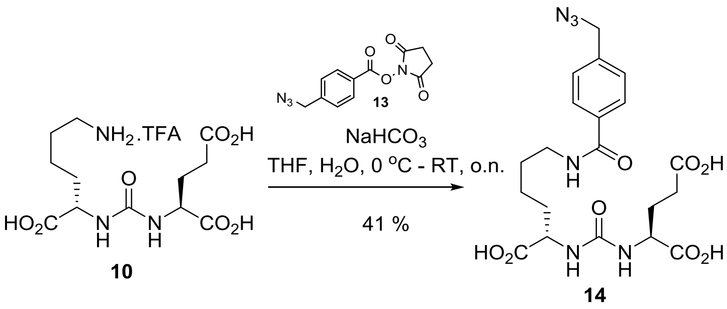

To a solution of amine **10** (723 mg, 1.60 mmol, corrected for solvent) and $NaHCO_3$ (806 mg, 9.60 mmol, 6.0 eq.) in water (22 mL) was added dropwise succinimide **13** (439 mg, 1.6 mmol, 1.0 eq.) in THF (22 mL), while cooling in ice. The mixture was stirred overnight. The reaction mixture was acidified with aq. 1 M HCl and concentrated *in vacuo* to give crude **14** (0.72 g) as a white solid. The product was purified by reversed phase column chromatography (120 g reverse phase - silica gel, gradient water : MeOH) to give compound **14** as a white solid (316 mg, 0.660 mmol, 41 %) with a purity of 98.2 % according to HPLC. ^{1}H NMR (299 MHz, Methanol-d_4) δ 8.47 (t, *J* = 5.5 Hz, 1H), 7.90 – 7.78 d, *J* = 7.9 Hz, 2H), 7.44 (d, *J* = 7.9 Hz, 2H), 4.44 (s, 2H), 4.29 (dt, *J* = 8.8, 4.7 Hz, 2H), 3.46 – 3.35 (m, 2H), 2.40 (dd, *J* = 8.6, 6.3 Hz, 2H), 2.25 – 2.05 (m, 2H), 1.99 – 1.80 (m, 2H), 1.67 (ddq, *J* = 20.1, 14.2, 7.2 Hz, 4H), 1.49 (p, *J* = 7.7 Hz, 2H). ES-MS *m/z* 479.2 [M+1].

2.3.5. 2-(2-(prop-2-yn-1-yloxy)ethoxy)ethan-1-ol (S3)

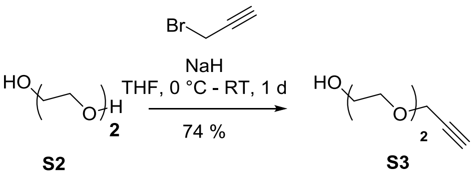

Diethylene glycol **S2** (10.0 g, 9.04 mmol) was dissolved in anhydrous THF (10.0 mL) and cooled to 0 ^{0}C. Sodium hydride (NaH, 1.5 g, 38.0 mmol) was slowly added to the solution. After 30 min a solution of propargyl bromide (3.5 g, 24.0 mmol) in THF (6.5 mL) was slowly added and the ice bath was removed. After 18 h the reaction was quenched with water and after extraction with DCM the combined organic layers were washed with brine and dried $MgSO_4$. After removal of the volatiles the residual oil was purified by column chromatography (silica gel, pentane : EtOAc; gradient 50% - 100% EtOAc) and yielded **S3** as a colorless oil (1.3 g, 9.0 mmol, 38 %). ^{1}H NMR (500 MHz, Chloroform-*d*) δ 4.22 – 4.20 (m, 2H), 3.76 – 3.68 (m, 6H), 3.63 – 3.60 (m, 2H), 2.44 (t, *J* = 2.4 Hz, 1H). This synthesis was adapted from literature[7] and ^{1}H NMR is in agreement with literature data.[8]

2.3.6. 2-(2-(prop-2-yn-1-yloxy)ethoxy)ethyl 4-methylbenzenesulfonate (18).

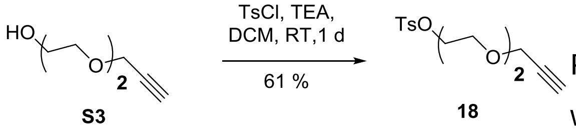

Propargyl-diethylene glycol **S3** (1.3 g, 9 mmol) was dissolved in dry DCM (8.5 mL). TsCl (2.03 g, 11 mmol) and Et_3N (1.8 g, 18 mmol) were added to the solution and was stirred for 18 h. The volatiles were removed *in vacuo* to obtain brownish triethylammonium chloride salts. The salts were washed with EtOAc and filtered. The volatiles were removed *in vacuo* and the residual oil was purified by column chromatography (silica gel, pentane : EtOAc; gradient 70:30% → 20:80%) and yielded **18** as a colorless oil (1.6 g, 5.5 mmol, 61 %). ^{1}H NMR (500 MHz, Chloroform-*d*) δ: 7.76 (d, *J* = 8.3 Hz, 2H), 7.31 (d, *J* = 8.3 Hz, 2H), 4.16 (dd, *J* = 6.0, 3.6 Hz, 4H), 3.73 – 3.67 (m, 2H), 3.66 – 3.58 (m, 4H), 2.48 – 2.38 (m, 4H), ^{1}H NMR is in agreement with literature data.[9]

2.3.7. 3-(2-(2-Fluoroethoxy)ethoxy)prop-1-yne (S4).

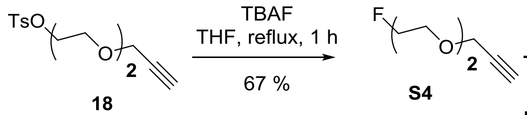

To tosylate **18** (300 mg, 1.35 mmol) in *t*-BuOH (8.2 mL) was added TBAF (1 M in THF, 2.27 mL, 2.27 mmol, 2.0 eq.). The yellow solution was stirred in a closed vial under nitrogen at 100 °C overnight. The mixture was concentrated. The residue was dissolved in DCM (10 mL) and washed with water (10 mL). The aqueous layer was extracted with DCM (10 mL). The combined organic layers were dried over Na_2SO_4, filtered and concentrated to give a dark yellow oil. The crude product was purified by column (silica gel, diethyl ether : pentane 1/9). The fractions with R_f 0.38 were concentrated (39 °C, 300 mbar) to give compound **S4** as a colorless oil (83 mg, 0.568 mmol, 42 %). ^{1}H NMR (300 MHz, Chloroform-*d*) δ 4.69 – 4.63 (m, 1H), 4.52 – 4.46 (m, 1H), 4.21 (d, *J* = 2.4 Hz, 2H), 3.83 – 3.78 (m, 1H), 3.72 (s, 5H), 2.43 (t, *J* = 2.4 Hz, 1H), in agreement with literature data.[10]

2.3.8. (((*S*)-1-carboxy-5-(4-((4-((2-(2-fluoroethoxy)ethoxy)methyl)-1H-1,2,3-triazol-1-yl)methyl)benzamido)pentyl)carbamoyl)-L-glutamic acid (F-PSMA-MIC03)

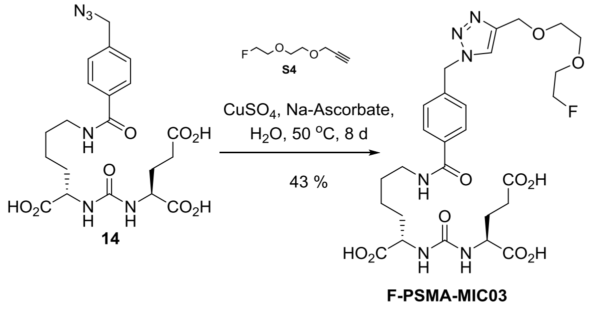

A mixture of compound **14** (100 mg, 0.209 mmol and alkyne **S4** (contains 20 wt% methyl-*tert*-butylether (TBME, 38 mg, 0.209 mmol, 1.0 eq.) in DMF (5 mL) was stirred under a nitrogen atmosphere. A suspension of $CuSO_4 \cdot 5\ H_2O$ (2.6 mg, 0.010 mmol, 0.05 eq.) and L-ascorbic acid

sodium salt (4.1 mg, 0.021 mmol, 0.10 eq.) in water (1.7 mL) was sonicated for 30 min and added and the reaction mixture was stirred at room temperature over the weekend. HPLC-MS indicated low conversion. A sonicated suspension of copper(II) sulfate pentahydrate (26 mg, 0.10 mmol, 0.5 eq.) and L-ascorbic acid sodium salt (41 mg, 0.21 mmol, 1 eq.) in water (3.7 mL) was added to the reaction mixture. Alkyne **S4** (19 mg, 0.105 mmol, 0.5 eq.) was added and the mixture was stirred at room temperature overnight. HPLC indicated 13 % conversion toward compound **F-PSMA-MIC03**. The reaction was continued at 50 °C for 1 week. Alkyne **S4** (31 mg, 0.209 mmol, 1.0 eq.) and a sonicated suspension of copper(II) sulfate pentahydrate (2.6 mg, 0.010 mmol, 0.05 eq.) and L-ascorbic acid sodium salt (4.1 mg, 0.021 mmol, 0.10 eq.) in water (0.5 mL) was added and the mixture was stirred over the weekend. The reaction mixture was concentrated *in vacuo* using a dry-ice cooler to give 0.34 g brown oil. The crude product was purified by preparative HPLC to give product **F-PSMA-MIC03** as a white solid (56 mg, 0.090 mmol, 43 %). ^{1}H NMR (299 MHz, Methanol-d_4) δ 8.02 (s, 1H), 7.86 – 7.79 (d, J = 8.2 Hz, 2H), 7.41 (d, J = 8.2 Hz, 2H), 5.67 (s, 2H), 4.65 (s, 2H), 4.61 – 4.55 (m, 2H), 4.45 – 4.37 (m, 2H), 4.19 (t, J = 6.3 Hz, 2H), 3.78 – 3.72 (m, 2H), 3.70 – 3.62 (m, 2H), 3.38 (t, J = 6.8 Hz, 2H), 2.42 – 2.30 (m, 2H), 2.12 (dt, J = 14.4, 6.8 Hz, 2H), 1.95 (m, 2H), 1.68 (dt, J = 16.6, 7.0 Hz, 4H), 1.49 (m, 2H). ^{19}F NMR (282 MHz, Methanol-d_4). δ 5.29 (tt, J = 47.8, 30.0 Hz). ^{13}C NMR (75 MHz, Methanol-d_4) δ 177.44, 176.61, 175.71, 168.13, 158.64, 145.08, 138.69, 134.64, 127.68, 127.56, 123.89, 83.76, 81.54, 70.26, 70.15, 70.00, 69.37, 63.55, 52.98, 39.54, 32.61, 28.79, 28.63, 22.67, 20.66.ES-MS *m/z* 625.4 [M+1]. ESI-HR-MS: *m/z* 647.2438 [M+Na] (theoretical: m/z 647.2447 [M+Na])

2.4. Synthesis of F-PSMA-MIC04

2.4.1. 2-(2-(2-(prop-2-yn-1-yloxy)-ethoxy)-phenyl)ethan-1-ol (19)

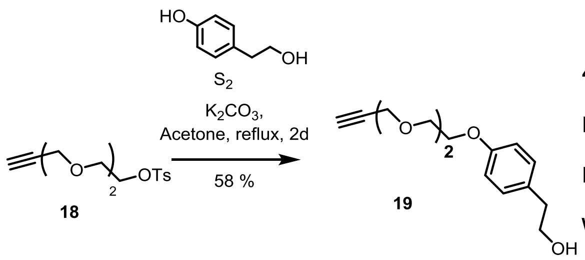

4-(2-Hydroxyethyl)phenol **S2** (0.24 g, 1.67 mmol), potassium carbonate (K_2CO_3, 714.91 mg, 5.16 mmol) and **18** (1.00 g, 3.35 mmol) were dissolved in 20 mL acetone and heated until reflux. The suspension was refluxed for 2 d. The purification was performed by column chromatography (silica gel, hexane: EtOAc, 50% - 100% EtOAc). Product **19** was obtained (0.287 g, 1.09 mmol, 58 %). ^{1}H NMR (500 MHz, Chloroform-*d*) δ = 7.09 (d, J = 8.5 Hz, 2H), 6.83 (d, J = 8.6 Hz, 2H), 4.18 (d, J = 2.4 Hz, 1H), 4.08 (dd, J = 5.2, 4.7 Hz, 1H), 3.86 – 3.79 (t, J= 3.7, 6.2Hz, 2H), 3.79 – 3.73 (t,J = 6.8, 6.7 Hz, 2 H), 3.73-3.66 (m, 6H), 2.76 (t, J = 6.7Hz, 1H), 2.43 (t, J = 2.4Hz, 1H), 1.95 (s, 1H). ^{13}C NMR (400 MHz, Chloroform-*d*) δ = 157.73, 130.62, 129.92, 144.78, 79.60, 74.54, 70.62, 69.79, 69.13, 67.45, 38.27, 29.68.

2.4.2. 4-(2-(prop-2-yn-1-yloxy)ethoxy)phenethyl 4-methoxylbenzensulfonate (20)

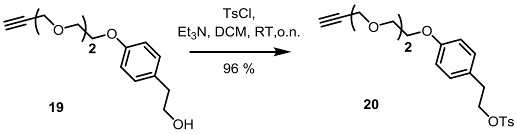

To the stirring solution of **19** with 0.6 mL anhydrous DCM, TsCl (195.8 mg, 1.03 mmol) and Et_3N (211 µL, 1.51 mmol) was added. The moisture was stirred overnight. Volatiles were removed *in vacuo*. The crude product **20** was purified by a column chromatography (silica gel, pentane : EtOAc, gradient 50-100% EtOAc). The product **20** was obtained in white crystals (0.275 g, 0.66 mmol, 96 %). ^{1}H NMR (500 MHz, Chloroform-*d*) δ = 7.73 – 7.66 (m, 2H), 7.28 (d, *J* = 8.1 Hz, 2H), 7.02 – 6.98 (m, 2H), 6.82 – 6.77 (m, 2H), 4.21 (d, *J* = 2.4 Hz, 2H), 4.16 (t, *J* = 7.1 Hz, 2H), 4.12 – 4.08 (m, 2H), 3.88 – 3.83 (m, 2H), 3.78 – 3.70 (m, 4H), 2.88 (t, *J* = 7.1 Hz, 2H), 2.43 (d, *J* = 2.3 Hz, 4H). ^{13}C NMR (101 MHz, Chloroform-*d*) δ = 157.73, 144.60, 129.85, 129.74, 114.72, 74.56, 70.81, 70.63, 69.76, 69.13, 67.42, 58.44, 34.49, 21.61.

2.4.3. 1-(2-fluoroethyl)-4-(2-(pro-2-yn-1-yloxy)ethoxy)benzene (21)

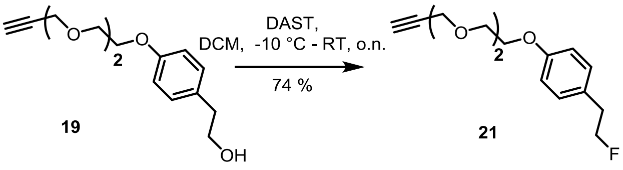

A solution of **19** (444 mg, 0.64 mmol) in DCM (5.0 mL) was cooled to -10 °C in an ice bath. Diethylaminosulfur trifluoride (DAST, 148 µL, diluted in 3.0 mL DCM) was added dropwise to the solution. After 30 min, the ice-bath was removed and the reaction was left overnight at room temperature. The reaction was quenched by adding 5 mL $NaHCO_3$ and left for 30 min. The reaction mixture was extracted with DCM, which was washed again with water, brine and dried over $MgSO_4$. Volatiles were removed in vacuo. The crude product **21** was purified by column chromatography (silica gel, Hexane: EtOAc, 20-50% EtOAc). Product **21** was obtained as white crystals (220.0 mg, 0.83 mmol, 74 %). ^{1}H NMR (500 MHz, Chloroform-*d*) δ = 7.13 (d, *J* = 8.5 Hz, 2H), 6.89 – 6.85 (m, 2H), 4.63 (t, *J* = 6.6 Hz, 1H), 4.53 (t, *J* = 6.6Hz, 1H), 4.21 (d, *J* = 2.4H, 2H), 4.13 – 4.10 (m, 2H), 3.90 – 3.82 (m, 2H), 3.80 – 3.66 (m, 4H), 2.97 (t, *J* = 6.6 Hz, 1H), 2.92 (t, *J* = 6.7 Hz, 1H), 2.43 (t, *J* = 2.4 Hz, 1H). ^{13}C NMR(400 MHz, Chloroform-*d*): δ 157.59, 129.89, 114.73, 84.28 (d, *J* = 167,9Hz), 79.61, 74.56, 70.61, 69.88, 69.13, 67.44, 58.43, 36.03 (d, *J* = 20,2 Hz).^{19}F NMR(400 MHz, CDCL3): δ -215.10.

2.4.4. (((*S*)-1-carboxy-5-(4-((4-((2-(2-(4-(2-fluoroethyl)phenoxy)ethoxy)ethoxy)methyl)-1H-1,2,3-triazol-1-yl)methyl)benzamido)pentyl)carbamoyl)-L-glutamic acid (F-PSMA-MIC04)

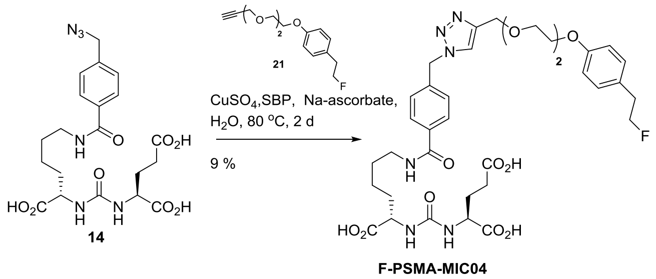

S14 (35.8 mg, 0.075 mmol) and **S18** (24mg, 0.090 mmol) were dissolved in 400 uL DMSO. $CuSO_4 \cdot 5\ H_2O$ (1.5 mg, 0.006 mmol), L-ascorbic acid sodium salt (2.58 mg, 0.013 mmol) and SBP (5.3 mg, 0.009 mmol) were dissolved in MilliQ water to obtain the click reagents in a green solution and added to the solution of **S14** and **S18** to obtain a reddisch reaction mixture, which was heated up to 60 ^{o}C. After 1 night, the temperature was increased until 80 ^{o}C and left stirring for another 2 d. The reaction mixture was diluted in MilliQ water and purified by preparative HPLC and freeze-dried by F-PSMA-MIC04 as a white, fluffy solid (5.2 mg, 0.007 mmol, 9 %). ^{1}H NMR (400 MHz, Methanol-d_4) δ 8.01 (s, 1H), 7.82 (m, 2H), 7.39 (m, 2H), 7.14 (m, 2H), 6.85 (d, *J* = 8.5 Hz, 1H), 5.74 – 5.62 (m, 2H), 4.65 (s, 2H), 4.55 (dq, *J* = 59.2, 8.0, 8.2, 8.3 Hz, 2 H) 4.35 – 4.21 (m, 2H), 4.14 (t, *J* = 5.7, 5.9 Hz, 1H), 4.07 (t, *J* = 5.6, 6.1 Hz, 1H), 3.84 (t, *J* = 5.9, 5.7 Hz, 1H), 3.79 (t, *J* = 6.0,5.7 Hz, 1H), 3.76 (m, 1H), 3.69 (m, 3H), 3.64 (m, 1H), 3.39 (t, *J* = 8.1 Hz, 6.4 Hz, 1H), 2.94 (t, *J* = 8.2 Hz, 8.1 Hz, 1H), 2.88 (t, *J* = 8.1 Hz, 8.8 Hz, 1H), 2.48 – 2.33 (m, 2H), 2.14 (m, 2H), 1.97 – 1.79 (m, 3H), 1.67 (m, i4H), 1.56 – 1.43 (m, 2H). Due to limited solubility we were unable to obtain ^{13}C NMR spectra in sufficient quality. ^{19}F NMR (400 MHz, Methanol-d_4) δ = -217.02 (m). ESI-HR-MS: m/z 767.3015 [M+Na] (theoretical: m/z 767.3023 [M+Na]).

3. Radiochemistry

All executed syntheses and experiments were performed in agreement with the local radiation safety regulations by well-trained / licensed radiochemists. This includes that all actions were performed in lead-shielded fumehoods and HPLC systems, reaction vials were kept in lead containers as much as possible and the radiochemists were working with long tweezers to increase the distance between the extremities of the radiochemist and radiation source. The radiation burden of the radiochemists were checked every month by the radiation safety manager. The FlowSafe synthesizer module was kept in a closed, lead-shielded HotCell to avoid any radiation burden for the radiochemists.

3.1. Fluorine-18 production and preparation.

Sep-Pak light Accel Plus QMA, pretreated with 10 mL 1.4% sodium hydrogen carbonate and 15 mL water and were dried under a helium flow. [^{18}F]Fluoride was produced by irradiation of [^{18}O]H_2O using the IBA Cyclone 18/9 Twin with a conical-5 target via the ^{18}O(p,n)^{18}F nuclear reaction. Subsequently, the [^{18}O]H_2O containing [^{18}F]fluoride was trapped on the pretreated Sep-Pak light Accel Plus QMA. [^{18}F]fluoride was eluted using mixture of 1 mg K_2CO_3 dissolved in 200 µL water and 15 mg Kryptofix K_{222} in 800 µL acetonitrile (MeCN). Solvents were evaporated at 130 ^{o}C using helium flow. One mL of anhydrous MeCN was added 3 times to remove residues of water.

3.2. Manual radiosynthesis of [^{18}F]9

Tosylate **8** (3.0 mg, 0.009 mmol) was azeotropically dried at 100 ^{o}C using anhydrous MeCN. After drying, **8** was dissolved in 300 µL anhydrous MeCN and added to the dried [^{18}F]fluoride (low amounts of radioactivity) and left to react for 10 min at 100 ^{o}C. After reaction, the product was cooled down and diluted into 100 mL 0.9 % NaCl solution to improve the removal of fluoride. The solution was passed over an Oasis HLB Plus LG Extraction cartridge and washed with 20 mL water. The product [^{18}F]**9** was eluted with 1.5 mL DMSO. Radiochemical yield (RCY)[11] was 21%.

3.3. Manual radiosynthesis of [^{18}F]PSMA-MIC01

An aqueous solution of click reagents containing $CuSO_4 \cdot 5\ H_2O$, (2.27 mg, 0.009 mmol), L-ascorbic acid sodium salt (3.61 mg, 0.018 mmol) and SBP (7.34 mg, 0.014 mmol) was prepared. Alkyne-Glu-urea-Lys **7** (5.0 mg, 0.01 mmol) was dissolved in 50 µL DMSO and diluted with 1.5 mL H_2O and added to the click reagent solution and mixed. This solution was added to the purified [^{18}F]**9** in DMSO and heated up until 80 ^{o}C for 20 min. After cooling down, the reaction mixture was diluted with 1.5 mL H_2O and is purified by HPLC (30% MeOH in H_2O with 0.1 % formic acid, with a flow of 5 mL/min). The peak eluting at approximately 20 min was collected and diluted with 60 mL H_2O and transferred over an Oasis HLB Plus LG Extraction cartridge, washed with 40 mL H_2O and eluted with 0.5 mL EtOH and 4.5 mL phosphate buffered saline (PBS).

Prior to every *in vivo* injection [^{18}F]PSMA-MIC01 underwent quality control performed by an independent person, to ensure that no radiochemical impurities influence the PET image and biodistribution. The quality control chromatogram is shown below:

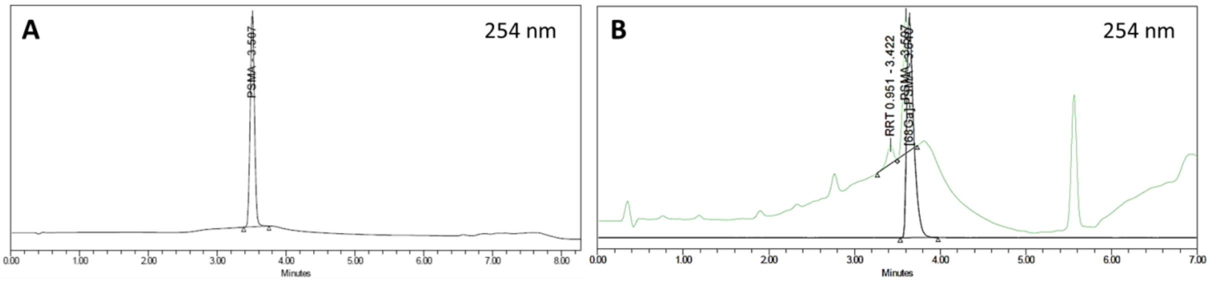

Figure S1. (A) UPLC chromatogram (at 254 nm) of the referene compound F-PSMA-MIC01. The obtained retention time was 3.5 min. (B) UPLC chromatogram of the [^{18}F]PSMA-MIC01 production for *in vivo* studies performed by Quality control. In green the UV chromatogram (254 nm) is shown and in black the radiodetector.

3.4. Manual radiosynthesis of [^{18}F]12

Tosylate **11** (3.0 mg, 0.007 mmol) was azeotropically dried at 100°C using anhydrous MeCN. After drying, **11** was dissolved in 300 uL anhydrous MeCN and added to the dried [^{18}F]fluoride and left to react for 10 min at 100°C. After reaction, the product was cooled down and diluted into 100 mL 0.9 % NaCl solution to improve the removal of fluoride. The solution was passed over an Oasis HLB Plus LG Extraction cartridge and washed with 20 mL water. The product [^{18}F]**12** was eluted with 1.5 mL DMSO. Radiochemical yield (RCY) of 66 %.

3.5. Radiosynthesis of [^{18}F]PSMA-MIC02.

An aqueous solution of click reagents containing $CuSO_4 \cdot 5\ H_2O$, (1.65 mg, 0.01 mmol), L-ascorbic acid sodium salt (2.65 mg, 0.013 mmol) and SBP (5.91 mg, 0.01 mmol) was prepared. **7** (3.0 mg, 0.07 mmol) was dissolved in 50 μL DMSO and diluted with 1.5 mL H_2O and added to the click reagent solution and mixed. This solution was added to the purified [^{18}F]**12** in DMSO and heated up until 90°C for 20 min. After cooling down, the reaction mixture was diluted with 1.5 mL H_2O and purified by HPLC (40% MeCN in H_2O with 0.1 % formic acid, with a flow of 5 mL/min). and the peak eluting at approximately 20 min was collected.

3.6. Automation with FlowSafe Click Synthesis Module.

After successful manual synthesis, the ^{18}F- radiolabeling was automated for scaling-up purposes using the FlowSafe continuous-flow micro-reactor platform for [^{18}F]PSMA-MIC01 and [^{18}F]PSMA-MIC02. Both the azide-tosylate (**8** or **11**) and [^{18}F]fluoride were azeotropically dried, dissolved in anhydrous MeCN and transferred through a 100 µL micro-reactor with a total flow speed of 80 µL/min, resulting in an effective reaction time of 75 s and an overall time of 17 min for complete transfer of both solutions through the micro-reactor. ^{18}F-fluorinated synthons were purified using a Solid Phase Extraction cartridge and eluted with DMSO into a vial containing the pre-dissolved acetylene-PSMA-binding ligand and click reagents in H_2O.

Table S1: A representative radio-TLCs of the fluorination step of the azide-tosylate 8, here indicated as [^{18}F]-S1 for synthon. It shows the higher radiochemical conversion and radiochemical purity of the synthon obtained by the Automized synthesizer FlowSafe Click.

Manual synthesis of [^{18}F]9	Manual purification of [^{18}F]9	Automized synthesis of [^{18}F]9	Automized purification of [^{18}F]9

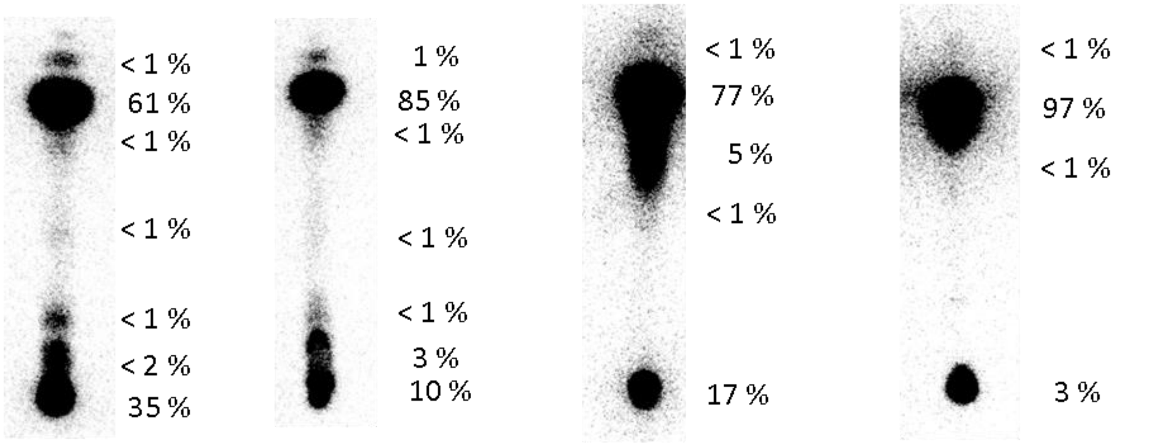

4. Radiotracer stability of [^{18}F]PSMA-MIC01 and [^{18}F]PSMA-MIC02

The stabilities of [^{18}F]PSMA-MIC01 and [^{18}F]PSMA-MIC02 were tested, to ensure the integrity of the tracer in solution. The reference compounds for both, F-PSMA-MIC01 and F-PSMA-MIC02, gave a retention time of 20 min. Since HPLC was used for purification, the first step is to collect the radioactive peak eluting at 20 min (A). After purification and formulation into an injectable solution of 10 % EtOH in PBS, the radiotracer was analysed by HPLC again (B),which was repeated after 2 h (C) and 4 h (D).

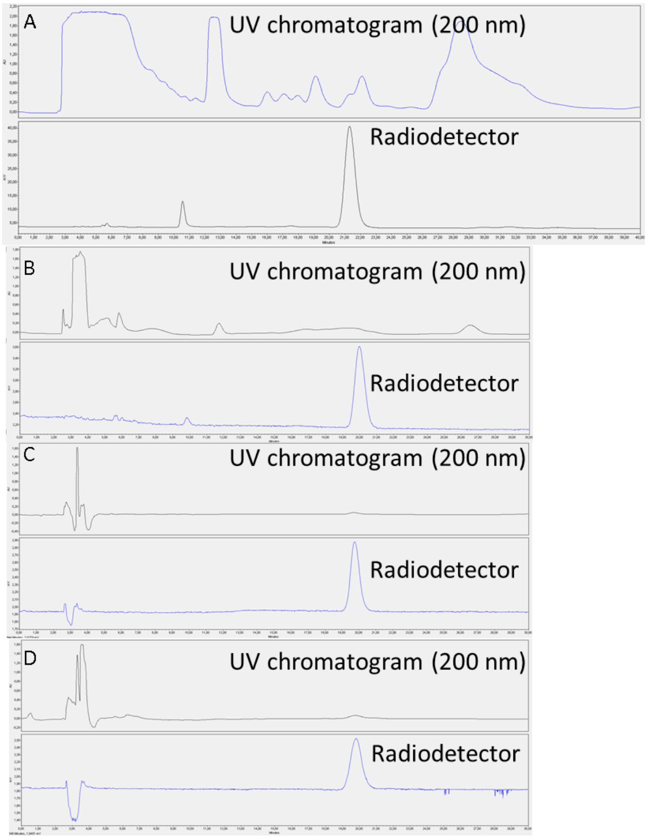

Figure S2: HPLC purification of [^{18}F]PSMA-MIC01 (A) and its stability test after formulation (0h), after 2 h (C) and after 4 h (D).

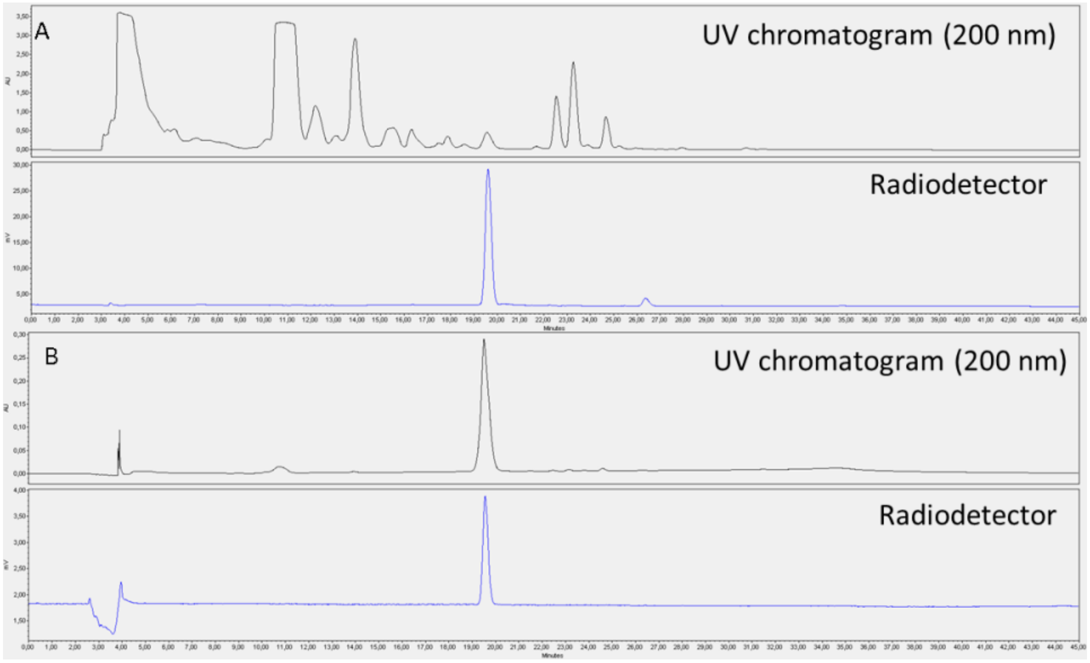

Figure S3: HPLC purification of [^{18}F]PSMA-MIC02 (A) and after 4 h (B).

5. Distribution coefficient Log*D*.

n-Octanol (0.49 mL) and PBS (0.41 mL, pH = 7.4) were pipetted into a 1.5 mL Eppendorf cup. 100 μL of the formulated final solution of [^{18}F]PSMA-MIC01 or [^{18}F]PSMA-MIC02 was added and vortexed for 1 minute and centrifuged for 5 min at 75000 rpm. The different layers were separated and 100 μL of each layer were measured on a γ-counter. Based on the counts per minute (CPM) of each fraction, the partition coefficient was measured with the following formula: log($CPM_{octanol}$/CPM_{PBS}). The obtained data are as followed.

Table S2. Counts per minute (CPM) of the 3 indidicually measured triplicates of [^{18}F]PSMA-MIC01 of n-octanol and PBS.

	Octanol (CPM)	PBS (CPM)	BLK (CPM)	LogD	Mean	SD
No 1.1	2673.07	2817174.28	93.59	-3.03	-3.02	
No 1.2	2823.43	2715328.33	93.59	-2.99		
No 1.3	2851.73	2947477.89	93.59	-3.02		
No 2.1	345.49	642597.17	93.59	-3.27	-3.28	
No 2.2	408	646465.72	93.59	-3.20		
No 2.3	394.49	640463.84	93.59	-3.21		
No 3.1	4534.46	2354084.13	50.84	-2.72	-2.79	
No 3.2	3690.69	2357291.58	50.84	-2.81		
No 3.3	5848.38	3956671.48	52.31	-2.83		
					-3.01	0.22

Table S3. Counts per minute (CPM) of the 3 indidicually measured triplicates of [^{18}F]PSMA-MIC01 of n-octanol and PBS

	Octanol (CPM)	PBS (CPM)	BLK (CPM)	LogD	Mean	SD
No 1.1	2940.13	4190486.12	58.39	-3.16	-3.16	
No 1.2	2995.66	4176463.25	58.39	-3.15		
No 1.3	2963.00	4259088.37	58.39	-3.17		
No 2.1	939.12	1893352.98	30.41	-3.32	-3.29	
No 2.2	1093.69	1913929.14	30.41	-3.26		
No 2.3	1020.97	1916376.47	30.41	-3.29		
					-3.22	0.09

6. Cell culture.

Prostate cancer cell lines PC-3 and LNCaP were obtained from the American Type Culture Collection (ATCC, Manassas, VA). Cells were cultured in RPMI-1640 (Lonza, Swiss), supplemented with 10 % fetal calf serum (FCS, Thermo Scientific Waltham, MA) at 37°C in a humidified 5 % CO_2 atmosphere. To enhance adherence of LNCaP cells tissue culture flasks and/or well plates were pre-coated with poly-*D*-lysine (Merck) according manufacture protocol. Cells were regularly checked for mycoplasma infection.

6.1. Cell binding studies.

For the determination of the binding affinity, a competitive binding radioassay was performed. Two 24 well plates were incubated with 50.000 cells 3 to 4 days prior to the cell experiments. After washing the cells twice with warm PBS, new medium was added. For the binding affinity, 50 µL of 14 different concentrations in triplicate ranging from 0.2 to 10000 nM of the cold reference compound F-PSMA-MIC01 were added to the wells shortly before 50 µL of the radioligand [^{68}Ga]PSMA-HBED-CC or [^{18}F]PSMA-1007 to reach a final volume of 500 µL in each well. After incubation of 90 min at 37°C under humidified conditions the cells were washed twice with ice-cold PBS to remove unbound tracer. Cells were detached from the wells using Trypsin supplemented with 25 % EDTA and incubated until cells were completely detached. 900 µL of medium was added and cells were transferred into tubes. The remaining activity in the cells were measured in a γ-counter. Afterwards, the cells were counted in a 1:1 solution of cell suspension and Trypan Blue. The tracer uptake was calculated using Microsoft Excel and corrected for the average number of cells and averaged. The $logIC_{50}$ value was calculated using the non-linear regression algorithm for a one-site FITlogIC50 using PrismGraphPad 7.2. The graphs represented show the average of the three individual experiments, while the mentioned $logIC_{50}$ was calculated from the mean of the three experiments;

Table S4. The $logIC_{50}$ values for the binding affinity study of F-PSMA-MIC01 against $[^{68}Ga]$PSMA-11.

	PSMA-11 precursor	F-PSMA-MIC01
No 1	-7.12	-7.24
No 2	-7.03	-6.86
No 3	-7.53	-6.59
Mean	-7.23	-6.89
SD	0.27	0.32

Table S5. The $logIC_{50}$ values for the binding affinity study of F-PSMA-MIC compounds against $[^{18}F]$PSMA-1007.

	F-PSMA-MIC01	F-PSMA-MIC02	F-PSMA-MIC03	F-PSMA-MIC04
No 1	-6.44	-7.35	-6.07	-6.61
No 2	-6.07	-7.34	-6.58	-6.82
No 3	-6.66	-7.51	-6.37	-6.31
			-7.22	
Mean	-6.39	-7.40	-6.56	-6.58
SD	0.30	0.09	0.49	0.26

7. Animal study.

The animal experiments were all performed according to the ethical guidelines and approved by the local animal welfare committee of the University of Groningen (IvD number 15166-06-001. All animals were caged separately in individually ventilated cages.

7.1. *In vivo* study.

7-12 week old immune deficient Balb/c nude mice were inoculated with 200 µL of a 1:1 suspension of medium containing approximately 4 x10^6 LNCaP cells (PSMA positive cells) or 5 x 10^6 PC3 cells (PSMA-negative cells) in RPMI-1640, and Matrigel Basement Membrane Matrix High concentration. The cells were subcutaneously inoculated on the right shoulder. After 3 to 5 weeks for LNCaP-xenografts, 2-3 weeks for PC3-xenografts or when a tumor size of $1cm^3$ was reached, the animals were transported to the PET imaging facility. For the blocking study, a 40 nM solution of 2-(phosphonomethyl)pentanedioic acid (2-PMPA) was prepared and 100 µL were injected via penile vein injection 30 min prior to the tracer injection. Animals were anesthetized at 5 % isoflurane and maintained under 2 % isoflurane enriched with O_2. The dynamic PET scan was started within 5 min after tracer injection with an acquisition time of 90 min. The static scan was performed 60 min after tracer injection with an acquisition time of 30 min.

7.2. Organ distribution and Metabolite analysis.

After sacrificing the animals, the organs were dissected and measured in a γ-counter. The obtained CPM's were normalized to %ID/g (Table shown in main text). For the metabolite analysis, urine and plasma samples were pipetted onto a TLC plate and run in a solution of 10 % MeCN in H_2O and read out in an Amersham Typhoon (GE).Additionally to the *ex vivo* biodistribution data shown in Table 1 in the main article, the image quantification based on the standardized uptake value (SUV_{meanBW}) was performed. The obtained data are represented in the following Table S6. Here, we show the results obtained by image quantification of the 4 different groups. Comparison of tumor uptake in PSMA-expressing LNCaP xenografts of (1) [^{68}Ga]PSMA-11 and (2) [^{18}F]PSMA-MIC01 (the same animals). (3) the PSMA-negative PC3 xenograft. (4) Confirmation of binding specificity of radiotracer [^{18}F]PSMA-MIC01, by blocking PSMA in LNCaP-xenografts prior to radiotracer injection] using the potent PSMA-inhibitor 2-PMPA.

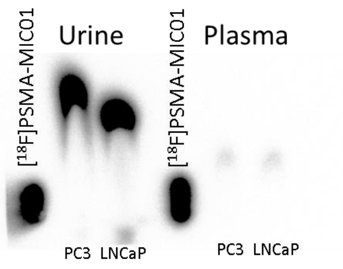

Figure S4. Representative metabolite analysis of two different animals bearing a PC3- or a LNCaP-xenograft. While in Plasma, no metabolite were formed, the urine samples were not conclusive.

In order to check for the significant differences, we also checked for the Cohen's d, a measurement to determine the effect size. It is calculated on the following formula:

$$Cohen'sD = \frac{Mean_1 - Mean_2}{average\ (Standard\ Deviations)}.$$

Table S6: Image quantification of the organ distribution calculated by SUV_{meanBW}. The values are represented as Mean ± SD %D/g. (n=6 mice for [^{18}F]PSMA-MIC01 on LNCaP-xenografts, n=5 mice for [^{68}Ga]PSMA-11 and [^{18}F]PSMA-MIC01 on PC3-xenograft).

	LNCaP (PSMA+) [^{68}Ga]PSMA-11	LNCaP (PSMA+) [^{18}F]PSMA-MIC01	PC3 (PSMA-) [^{18}F]PSMA-MIC01	LNCaP-blocked (PSMA+) [^{18}F]PSMA-MIC01
**Tumor**	0.4 ± 0.3	0.6 ± 0.4	0.4 ± 0.2	0.3 ± 0.1
**Kidney**	2.8 ± 1.0	4.4 ± 1.4	5.1 ± 1.9	2.6 ± 1.7
**Bladder**	1.9 ± 1.6	10.2 ± 6.7	3.9 ± 0.4	20.7 ± 12.4
**Brain**	0.7 ± 1.3	0.2 ± 0.1	0.2 ± 0.1	0.1 ± 0.0
**Heart**	0.3 ± 0.1	0.4 ± 0.2	0.7 ± 0.2	0.4 ± 0.1
**Muscle**	0.1 ± 0.2	0.2 ± 0.1	0.3 ± 0.1	0.2 ± 0.1
**Liver**	0.4 ± 0.2	0.8 ± 0.2	1.5 ± 0.5	0.8 ± 0.4

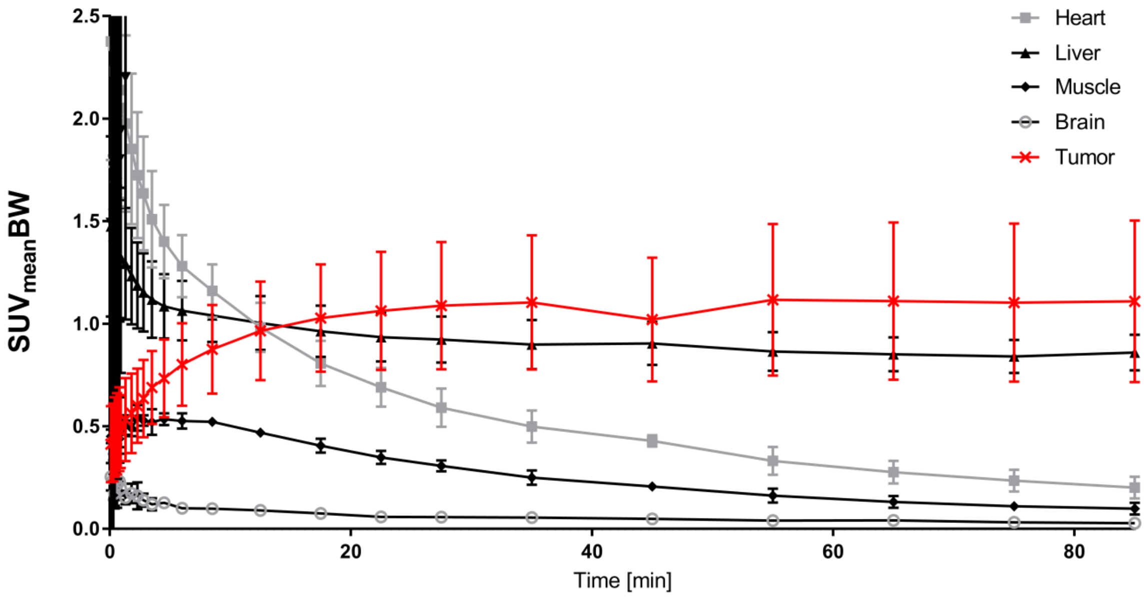

Figure S5: Time-activity-curves with standard deviations. Herein, the Kidney are removed, due to the high uptake due to the renal clearance. The values are represented as Mean ± SD (n=6).

8. Computational Details

8.1. Molecular docking

The proteins were prepared through the Protein Preparation Wizard in Maestro, performing the assignment of bond orders, hydrogens addition, hydrogen bonds definition and optimization, waters removal and restrained minimization with the OPLS3 force field.[12] The grid was created through the Receptor Grid Generation, picking the ligand to define the centroid of the receptor box, and rotation of the hydroxyl groups of Ser501, Ser513, Tyr552,

Tyr700 were allowed. LigPrep was used to prepare the ligands and to generate possible states at pH 7.0 ± 2.0 with Epik. The ligands were docked with Glide XP,[13] flexible, performing post-docking minimization on 30 poses and writing out at most 20 poses per ligand. The top-ranked poses were selected for all the ligands, except for redocking of **MeO-P4** and docking of **F-PSMA-MIC02.** For **MeO-P4**, the 6th-ranked pose was selected because it showed the lowest Root Mean Square Deviation (RMSD) value (vide infra). For **F-PSMA-MIC02**, the 2nd-ranked pose was selected because the additional aromatic ring engaged in the target π-π interaction.

Two protein-ligand complexes were considered for this docking study, PSMA complexed with **MeO-P4** [14] (PDB: 2XEJ) and **ARM-P2** [14] (PDB: 2XEI), so that two distinct conformation of Trp541 were included (see main text). In those PDB complexes, electron density was absent for the PEG chain (due to its flexibility and lack of specific interactions) and, in 2XEI, for the nitro groups (because the ring is in more different conformations).

Redocking of the co-crystallized ligands were carried out on 2XEJ and 2XEI (Figures S5 and S6), with RMSD values of 2.914 Å and 1.580 Å, respectively. Regarding 2XEJ, the highest-ranked pose with the lowest RMSD value was the 6th-ranked pose: these high RMSD values for 2XEJ are consistent with the large flexibility of the PEG linker of the ligands.

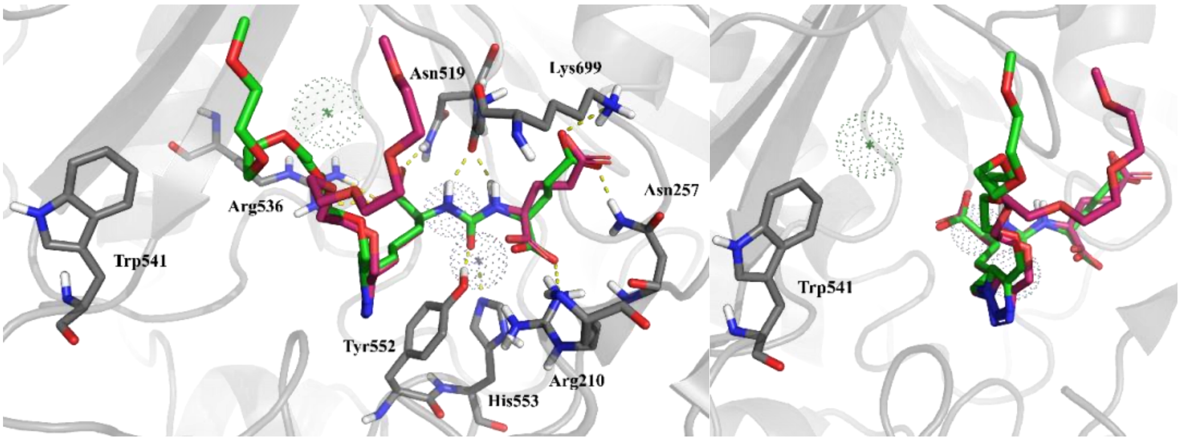

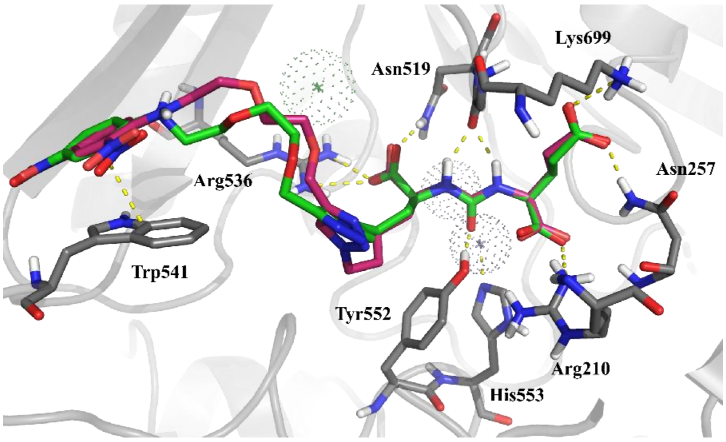

Figure S6. (left) Redocking (red) of MeO-P4 (green) into 2XEJ. (right) Rotated detail of the PEG chain conformations. Hydrogen bonds are depicted as yellow dashed lines.

Figure S7. Redocking (red) of ARM-P2 (green) into 2XEI. Hydrogen bonds and π-π stacking are depicted as yellow dashed lines.

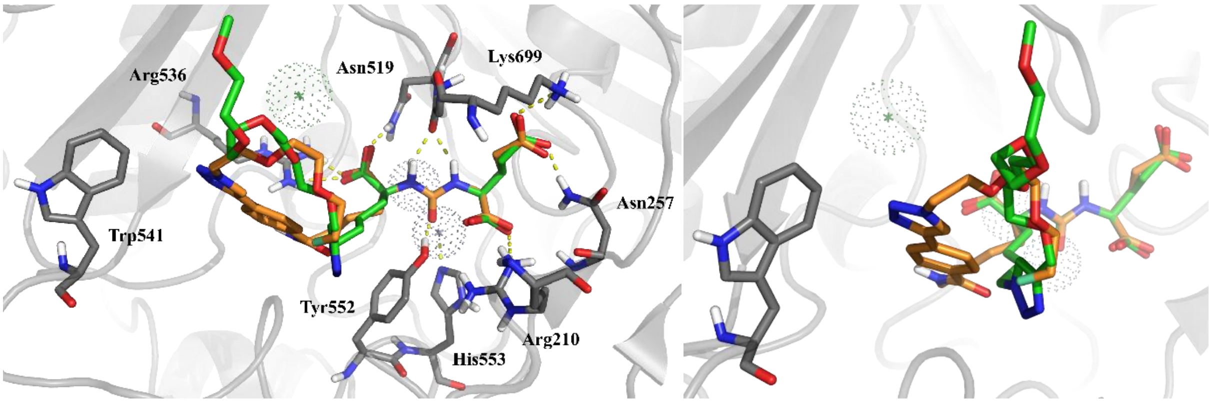

Figure S8. (left) Docking pose of F-PSMA-MIC01 (orange) into 2XEJ, superimposed with MeO-P4 (green) co-crystallized with the enzyme. (right) Rotated detail of the PEG chain conformations. Hydrogen bonds are depicted as yellow dashed lines.

F-PSMA-MIC01 and **F-PSMA-MIC03** (Figure S7 and S8) show similar docking poses to the parent compound **MeO-P4**, especially in the Glu-urea-Lys motif. The flexible diethylene glycol chain was not involved in any specific interaction, in line with the absent electron density of this portion of the ligand in the complex.

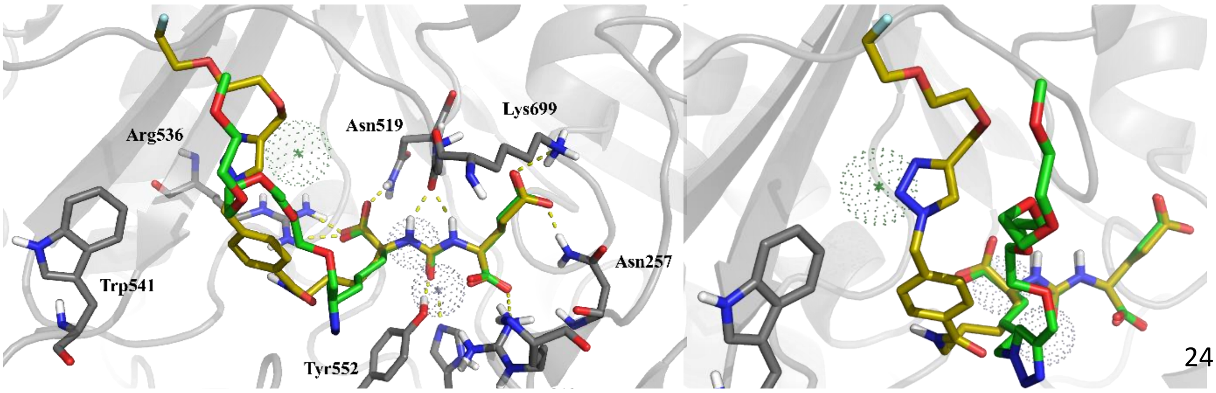

Figure S9 (left) Docking pose of F-PSMA-MIC03 (yellow) into 2XEJ, superimposed with MeO-P4 (green) co-crystallized with the enzyme. (right) Rotated detail of the PEG chain conformations. Hydrogen bonds are depicted as yellow dashed lines.

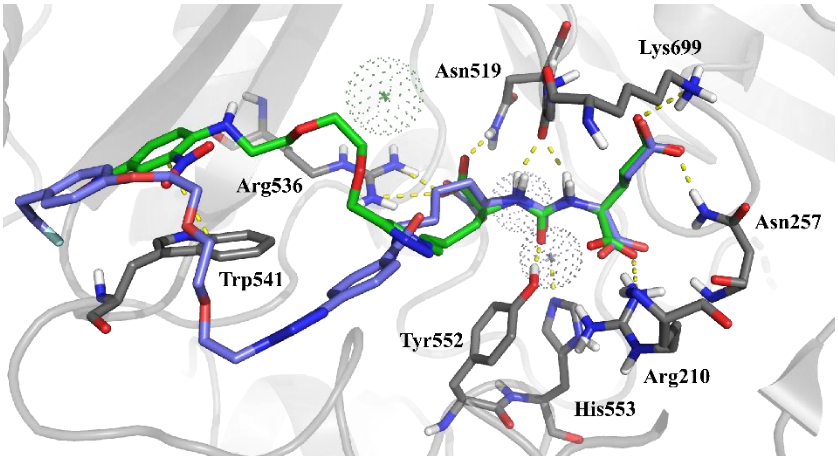

Figure S10. Docking pose of F-PSMA-MIC02 (violet) into 2XEI, superimposed with ARM-P2 (green) co-crystallized with the enzyme. Hydrogen bonds and π-π stacking are depicted as yellow dashed lines.

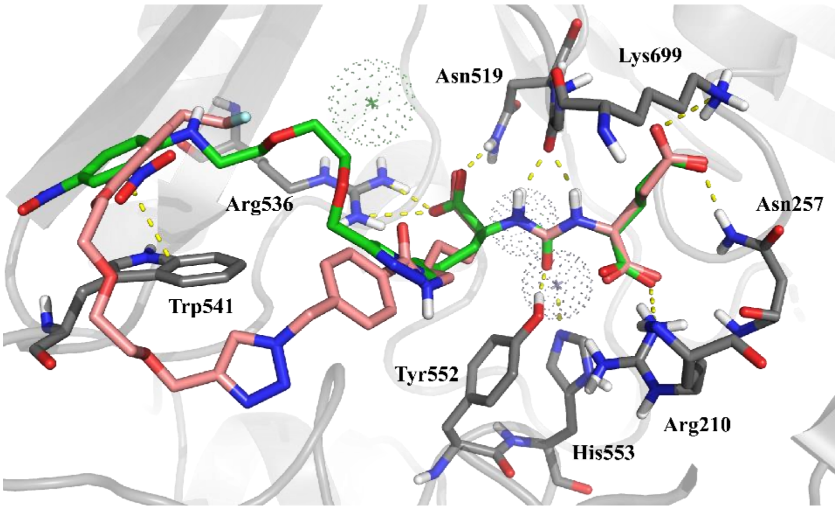

Figure S11. Docking pose of F-PSMA-MIC04 (pink) into 2XEI, superimposed with ARM-P2 (green) co-crystallized with the enzyme. Hydrogen bonds and π-π stacking are depicted as yellow dashed lines.

F-PSMA-MIC02 and **F-PSMA-MIC04** (Figure S10 and S11) showed similar docking poses to the parent compound **ARM-P2**, especially in the Glu-urea-Lys motif, and the aromatic rings are able to reach Trp541 in the arene-binding site (Figure S13). However, they have a suboptimal orientation for π-π interactions (Table S7) compared to the cutoffs of the Ligand Interaction Diagram.

In Maestro's User Manual, a π-π interaction is defined as an interaction between two aromatic rings in which either (a) the angle between the ring planes is less than 30° and the distance between the ring centroids is less than 4.4 Å (face-to-face), or (b) the angle between the ring planes is between 60° and 120° and the distance between the ring

centroids is less than 5.5 Å (edge-to-face). These criteria are the adaptation of literature cutoffs.[15]

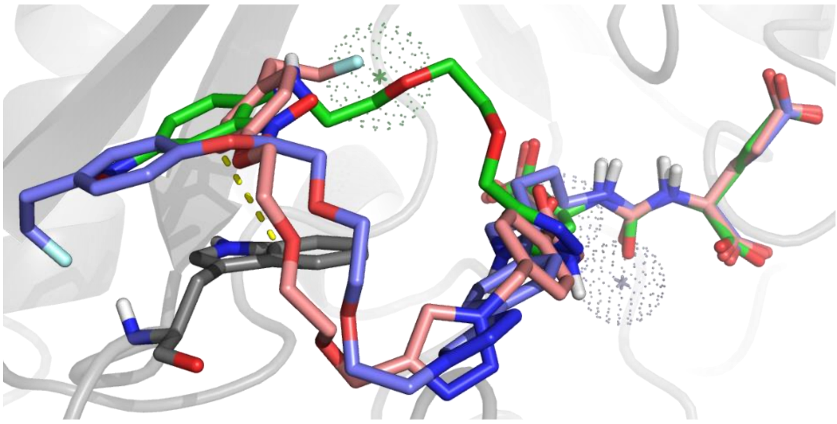

Figure S13. Detail of the π-π interactions for the co-crystallized ARM-P2 (green), the docked F-PSMA-MIC02 (violet) and F-PSMA-MIC04 (pink) into 2XEI.

Table S7. Geometry measurements for the aromatic rings compared to the cutoffs for a face-to-face π-π interaction.

**Entry**	**Ring distance (Å)**	**Ring angle (°)**
ARM-P2 (crystal structure)	4.8	23
F-PSMA-MIC02 (docking)	6.6	18
F-PSMA-MIC04 (docking)	4.5	42
face-to-face interaction cutoff	*< 4.4*	*< 30*

The π-π interactions behavior of these three compounds was further evaluated with an MD study.

8.2. Molecular Dynamics

The protocol was adapted from a previous MD study on the same system.[13] The crystal structure of **ARM-P2** in complex with PSMA (PDB: 2XEI), the second-best docking pose of **F-PSMA-MIC02** into 2XEI and the top-ranked docking pose of **F-PSMA-MIC04** into 2XEI were used to setup the MD calculations. The structures were embedded in a orthorhombic box of circa 20600 TIP3P [16] water molecules, the dimension of the box was circa 106x86x83 Å. The net charge of the system was neutralized by addition of five sodium ions to the solvent box. The total number of atoms was circa 73,000 atoms. The simulations were performed with the Desmond molecular dynamics package,[17] with default settings for bond-constrains, Van der Waals and electrostatic interactions cutoffs, PME method for long range electrostatic interactions.

Each system was subjected to the following relaxation and equilibration protocol: 100 ps of Brownian dynamics at 10 K in the NVT ensemble with harmonic restraints (50 kcal/mol/A)[14] on the solutes heavy atoms, followed by 12 ps in the NVT ensemble (Berendsen thermostat)[20] at 10 K and retaining harmonic restraints on the solutes heavy atoms, followed by 12 ps in the NPT ensemble (Berendsen thermostat and barostat) at 10 K and retaining harmonic restraints on the solutes heavy atoms, followed by 24 ps in the NPT ensemble (Berendsen thermostat and barostat) at 300 K and retaining harmonic restraints on the solutes heavy atoms, followed by 24 ps in the NPT ensemble (Berendsen thermostat and barostat) at 300 K without harmonic restraints on the solutes heavy atoms. The production simulations were run for 100 ns in the NPT (300 K, 1 bar, Martyna-Tobias-Klein barostat and Nose-Hoover thermostat),[21,22]in three replicas. Coordinates were saved every 100 ps and analyzed in Maestro.

Ring distances and ring angles between the aromatic ring of the ligands and the six-membered ring of Trp541 were measured in Maestro, through the Plot>Measurements tool. Following Maestro's User Manual, π-π interaction cutoffs were defined as follows: (a) the angle between the ring planes is less than 30° and the distance between the ring centroids is less than 4.4 Å (face-to-face), or (b) the angle between the ring planes is between 60° and 120° and the distance between the ring centroids is less than 5.5 Å (edge-to-face). These criteria were the adaptation of literature cutoffs[15]. The choice of the six-membered ring in the indole of Trp541 as the ring for the distances and angles measurements was supported by QM calculations (see next section), because the negative electron potential was localized on top of the six-membered ring.

The following Figures (12 -17) depict the time traces of ring distances (left) and ring angles (right) between the aromatic ring of the ligands and the six-membered ring of Trp54, over the course of the three MD replicas for each of the protein-ligand complex. Replica 1 is red, Replica 2 is green and Replica 3 is green. The areas that correspond to the geometry cutoffs for face-to-face and edge-to-face π-π interactions are highlighted in pink and yellow, respectively.

PDB complex of ARM-P2 (2XEI)

**Ring distance (Å)**	
Average	St dev
4.1	0.3

**Ring angle (°)**	
Average	St dev
15	6

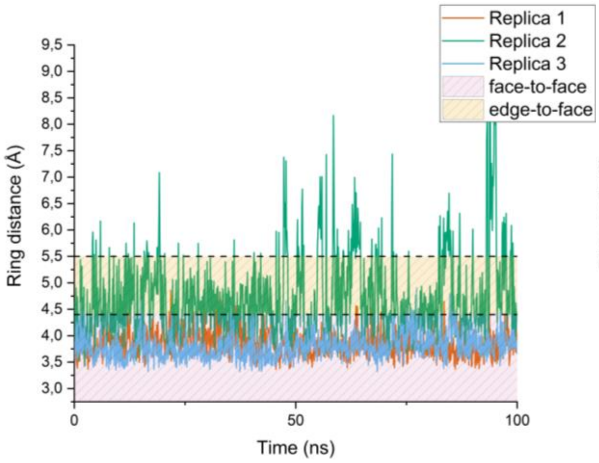

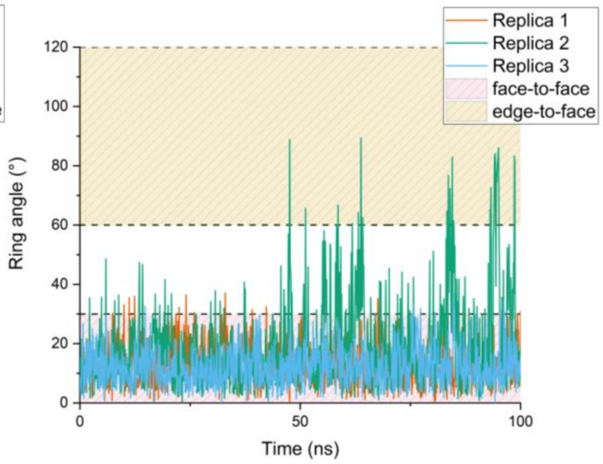

Figure S14 and S15. See the description above.

F-PSMA-MIC02

Ring distance (Å)	
Average	St dev
6.1	0.8

Ring angle (°)	
Average	St dev
34	12

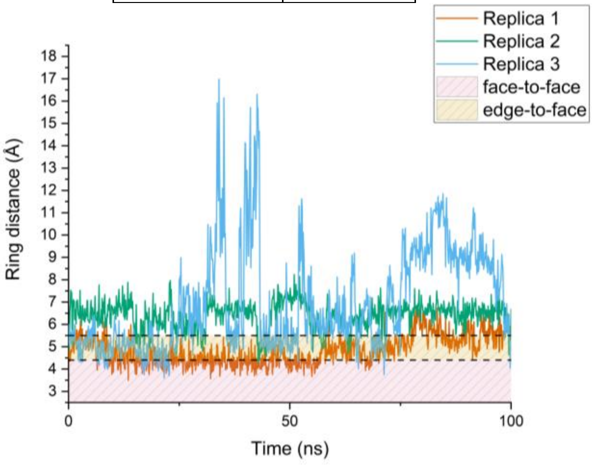

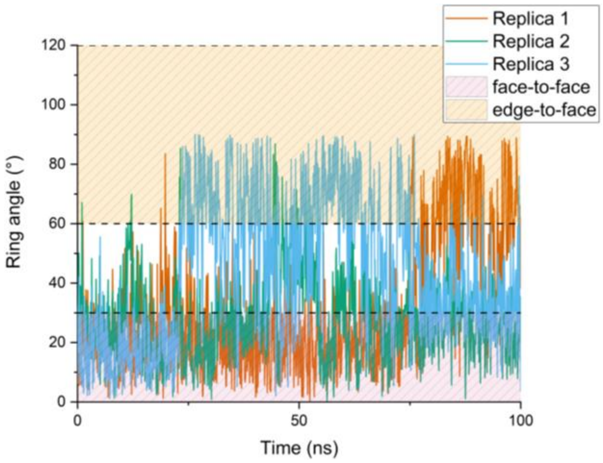

Figure S16 and S17. See the description above.

F-PSMA-MIC04

Ring distance (Å)	
Average	St dev
5.7	0.6

Ring angle (°)	
Average	St dev
43	13

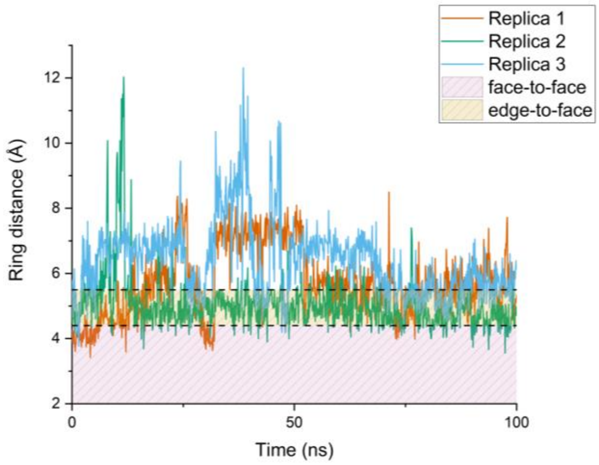

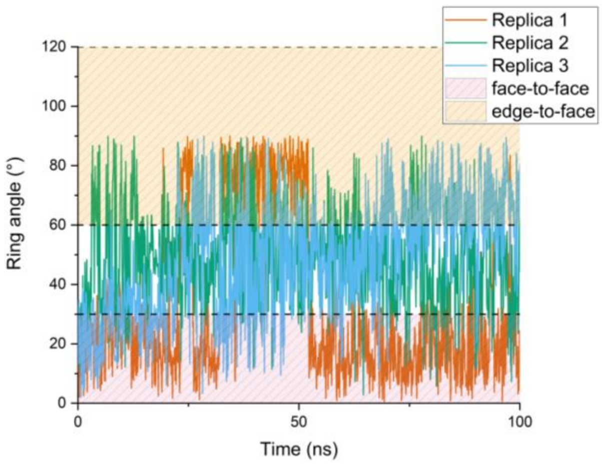

Figure S18 and S19. See the description above.

In the following pages, extracts from the Simulation Interactions Diagram Reports for each replica are reported with short qualitative description of the trajectory. In the top figures, RMSD evolutions of the protein (left Y-axis). Ligand RMSD (right Y-axis) is an indication of how stable the ligand is with respect to the protein and its binding pocket. In the bottom figures, monitoring of protein-ligand interactions, divided into four types: Hydrogen Bonds, Hydrophobic (π-cation, π- π, non-specific interactions), Ionic and Water bridges.

2XEI_1-replica

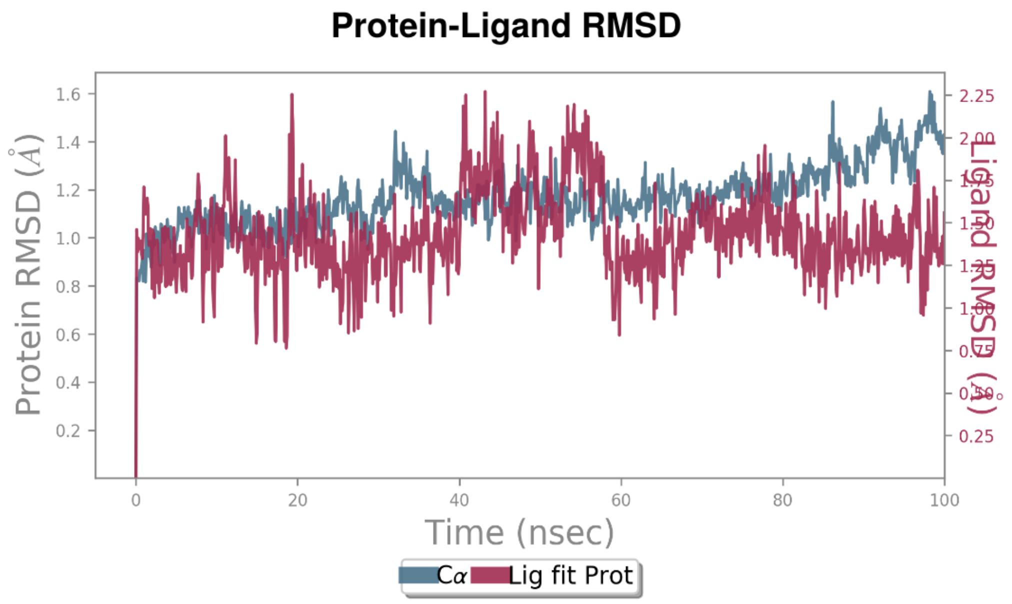

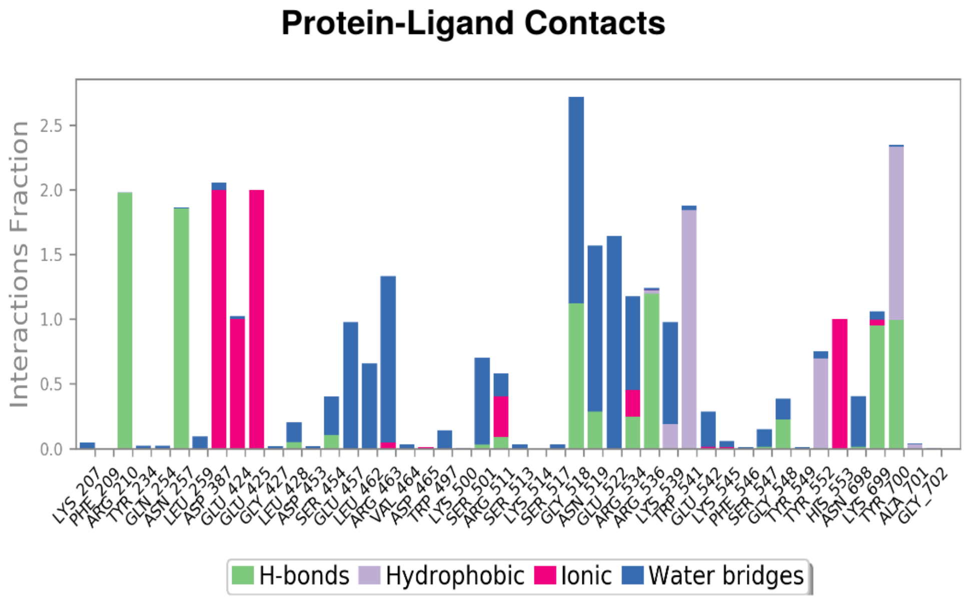

The face-to-face π-π stacking of the dinitrophenyl ring with Trp541 was very stable. 1,2,3-triazole engaged in cation-π interactions with Arg463 and Lys539, and in π-π interactions with Tyr700.

2XEI_2-replica

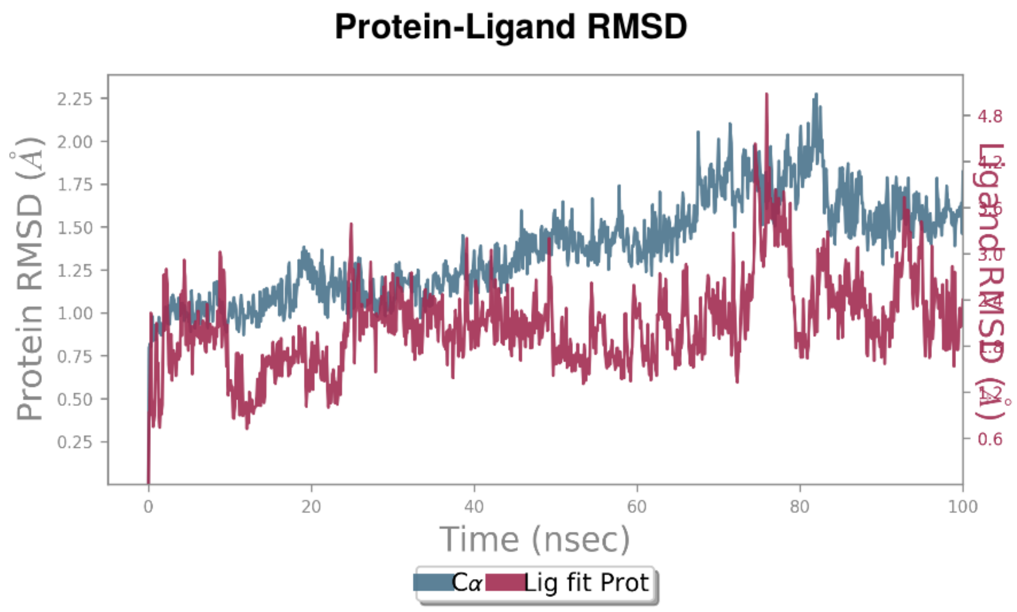

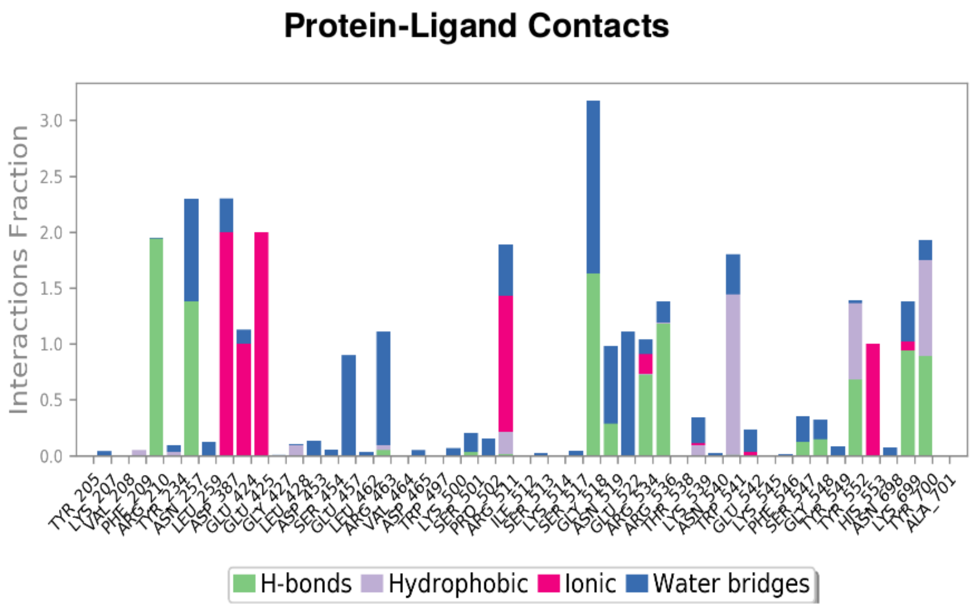

The face-to-face π-π stacking was less stable because of the rotated conformation of Trp541 (Figure S12), while 1,2,3-triazole forms π-π interactions with Tyr 700.

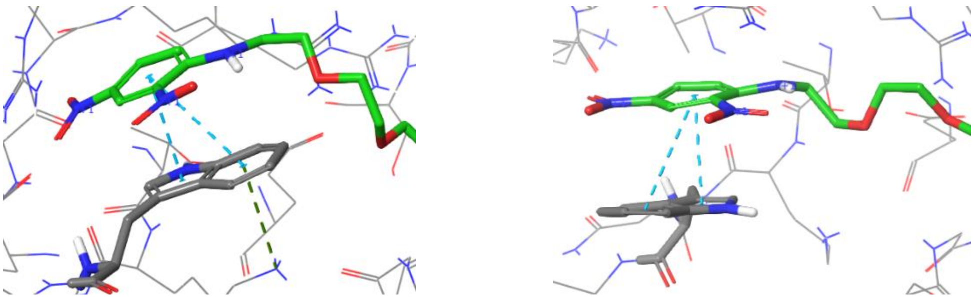

Figure S18. First frame of the first replica (left) and the second replica (right) of the 100 ns MD runs of 2XEI. The conformation of Trp 541 in the second replica is rotated compared to the usual orientation.

2XEI_3-replica

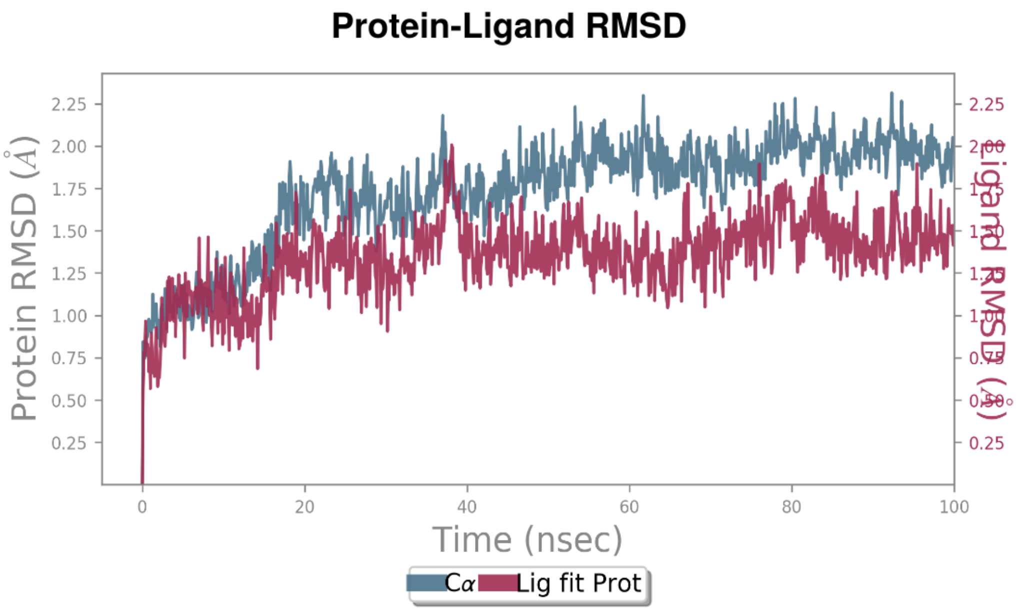

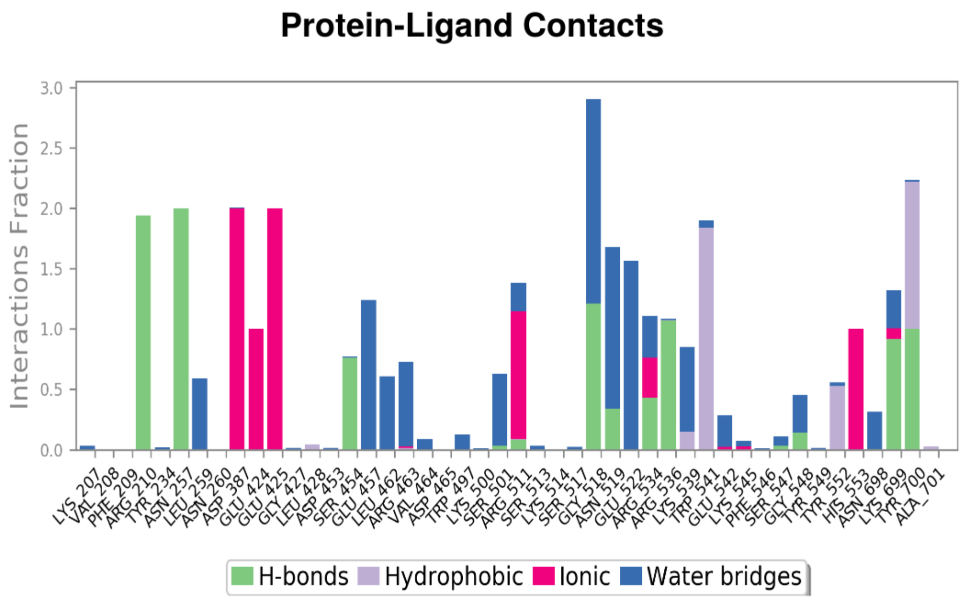

The trajectory was very similar to the first replica. The face-to-face π-π stacking was remarkably stable and 1,2,3-triazole forms cation-π interactions with Arg463 and Lys539, and π-π interactions with Tyr700.

F-PSMA-MIC02_1-replica

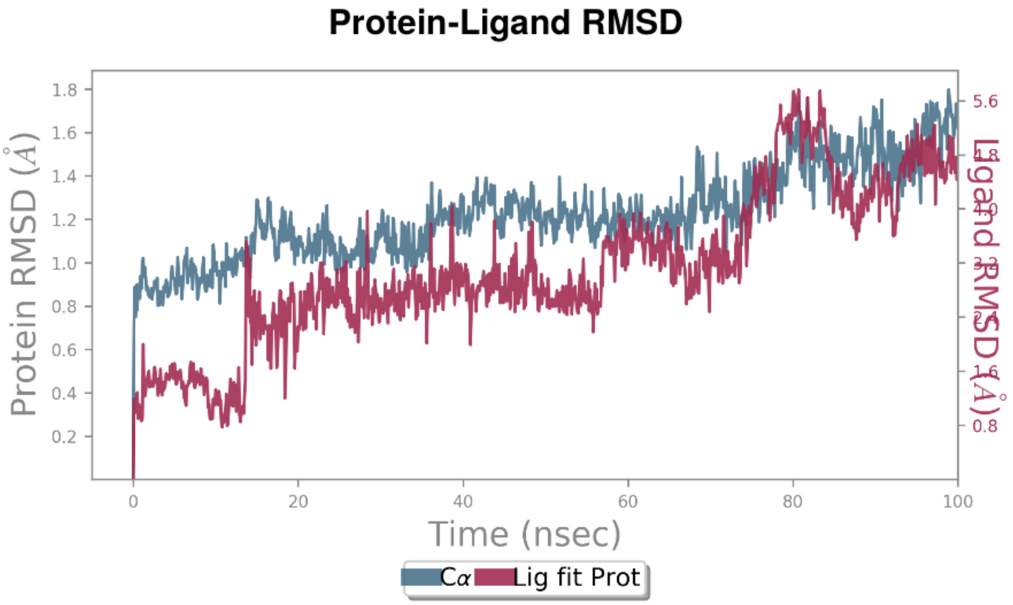

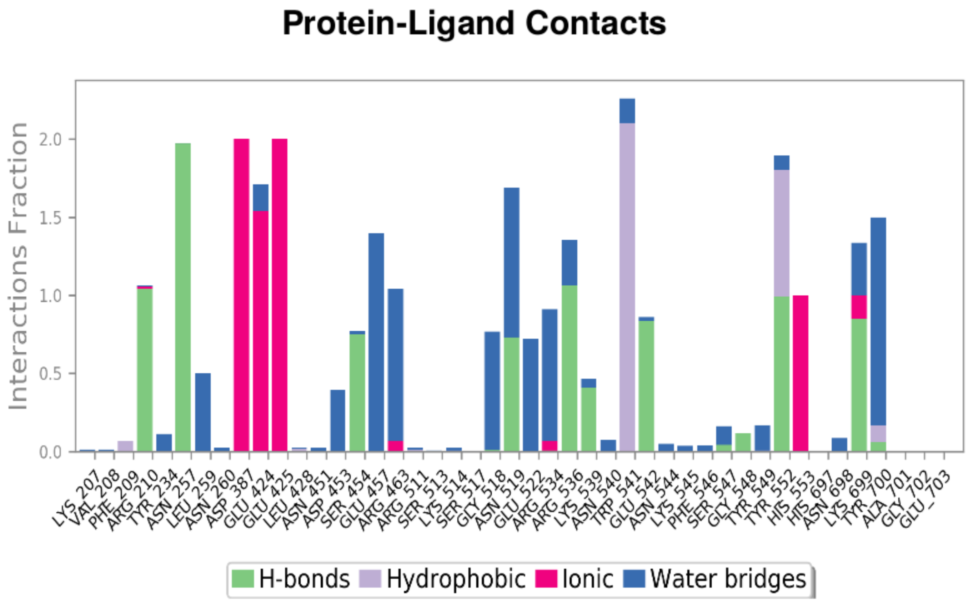

The face-to-face π-π stacking was stable for the first 60 ns, then it was broken and the PEG chains folds on itself, leading the aromatic ring to interact with the benzamide linker. 1,2,3-triazole engaged in a face-to-face π-π stacking with Trp541 for 70% of the run.

F-PSMA-MIC02_2-replica

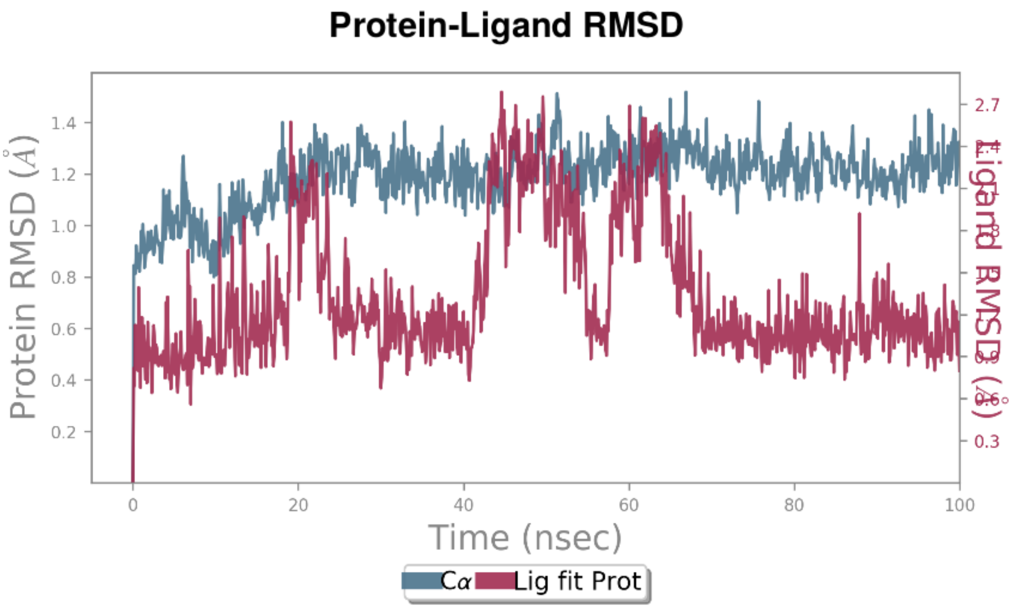

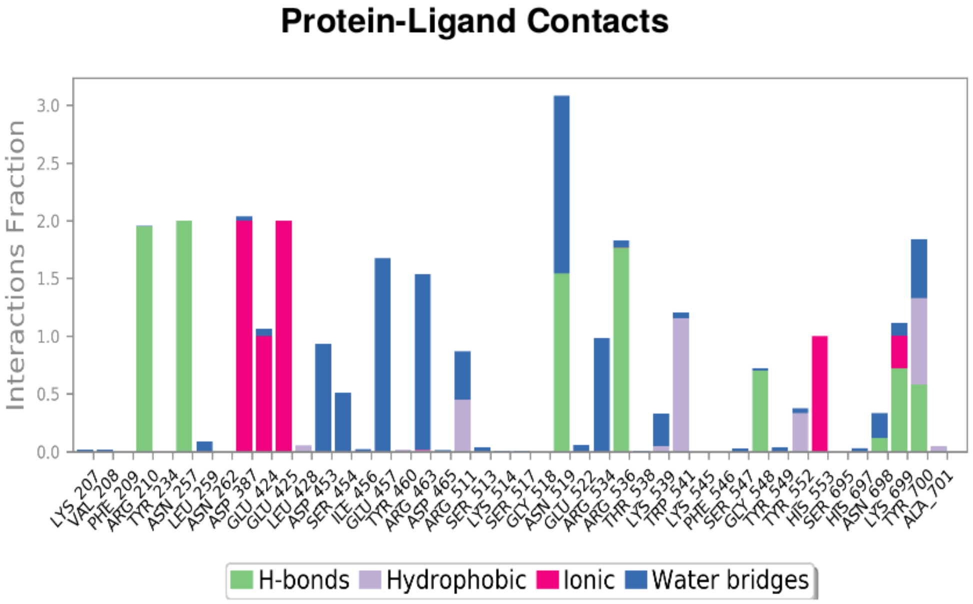

The aromatic ring engaged in cation-π interactions with **11**, while the benzamide linker forms edge-to-face π-π interactions with Trp541 and Tyr700. The latter contact hindered the interaction between the aromatic ring and Trp 541.

F-PSMA-MIC02_3-replica

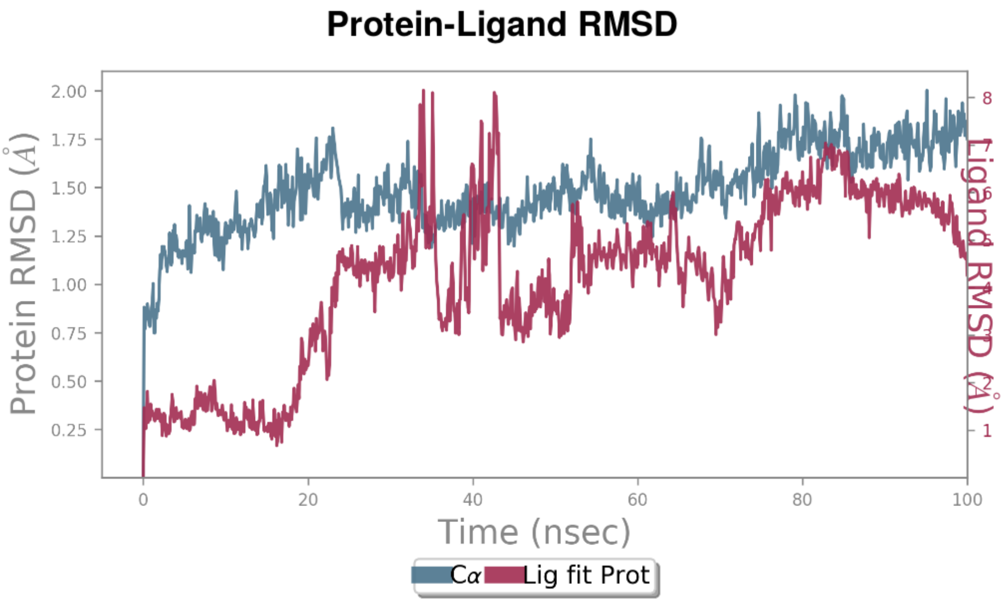

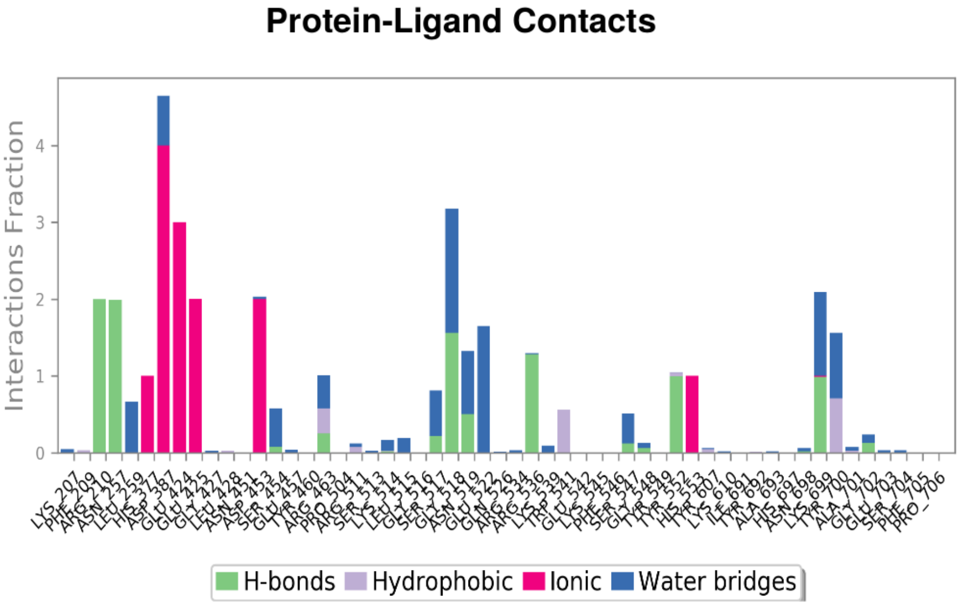

The aromatic ring engaged in cation-π interactions with Arg511 and in π-π interactions with Trp541. The benzamide linker gave cation-π interactions with Arg463 and π-π interactions with Trp541. Around 30 ns, Trp541 moved into the flipped conformation and the π-π stacking is not formed anymore. Towards the end of the simulation, the PEG chain folded on itself and the aromatic ring forms π-π interactions with 1,2,3-triazole.

F-PSMA-MIC04_1-replica

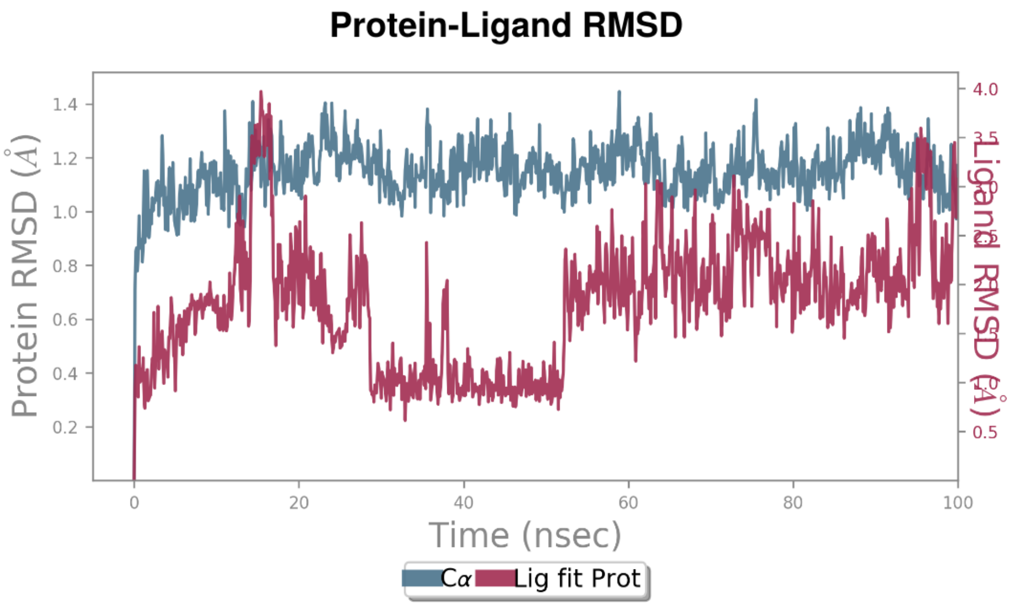

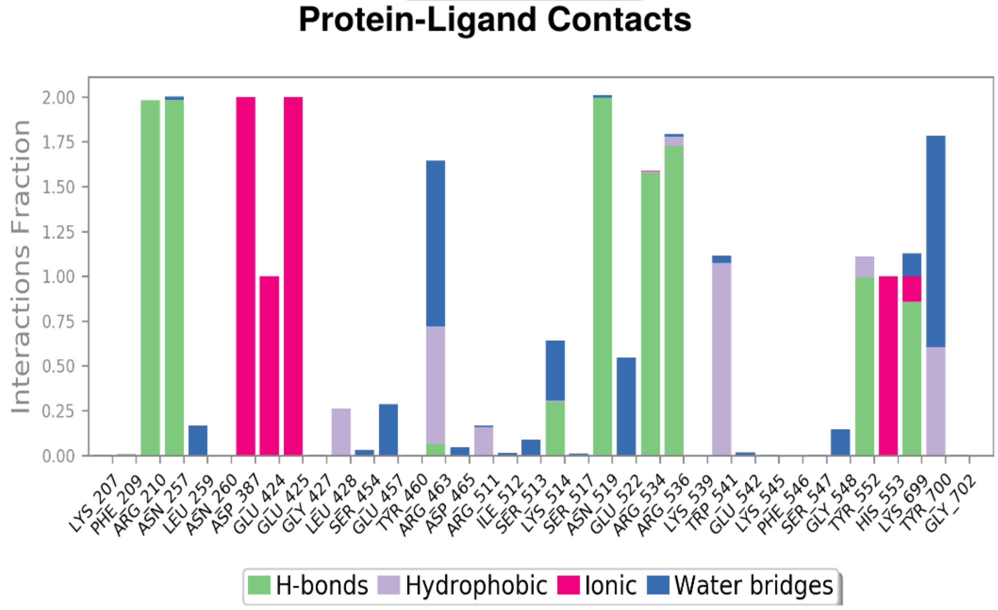

The face-to-face π-π stacking was stable for the first 10 ns. After 15 ns, Trp541 flips down (in contrast with the usual flipped-up or flat conformations) and engaged in π-π interactions with 1,2,3-triazole. The aromatic ring engaged in cation-π interactions with Arg511. The benzamide linker gave cation-π interactions with Arg463.

F-PSMA-MIC04_2-replica

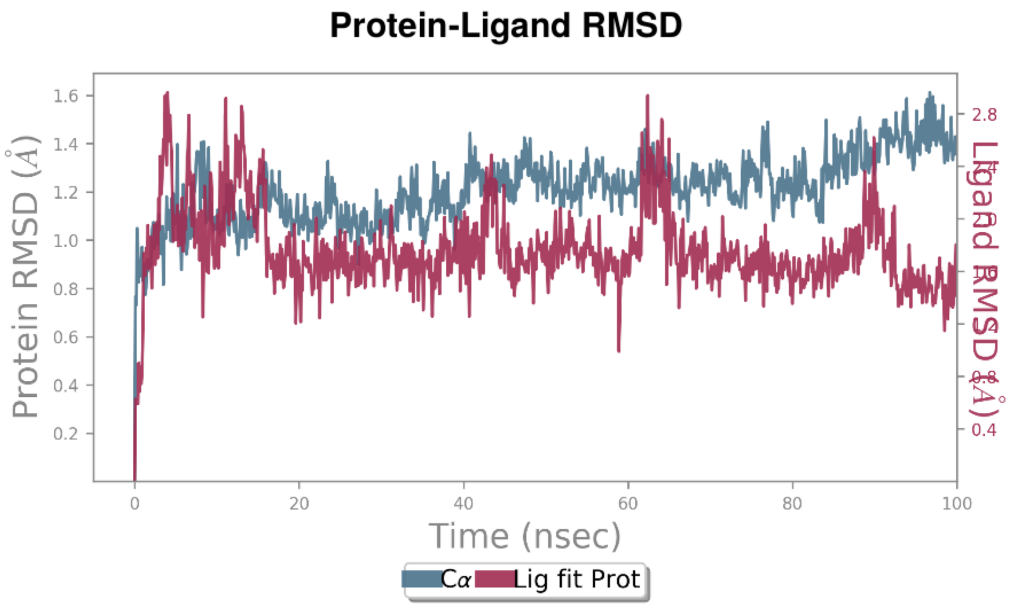

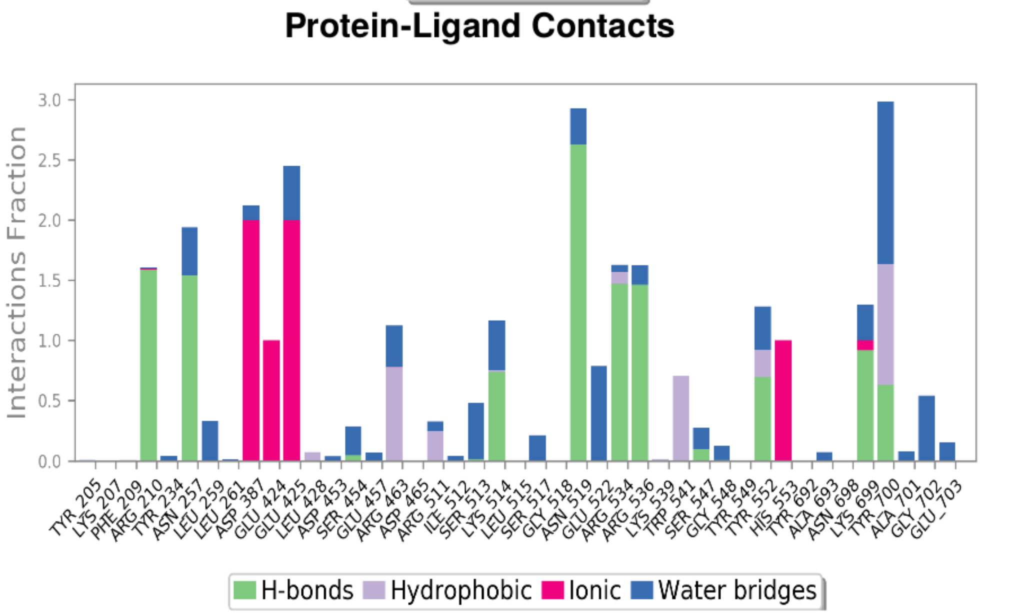

The aromatic ring formed edge-to-face π-π interactions with Trp541 and cation-π interactions with Arg511. The benzamide linker gave cation-π interactions with Arg463 and π-π interactions with Tyr700.

F-PSMA-MIC04_3-replica

Protein-Ligand RMSD

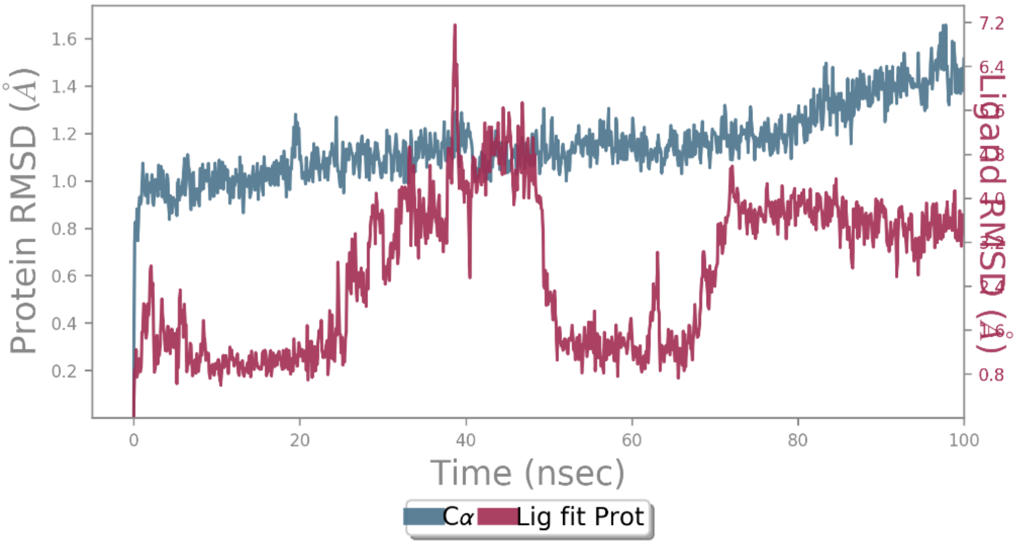

Protein-Ligand Contacts

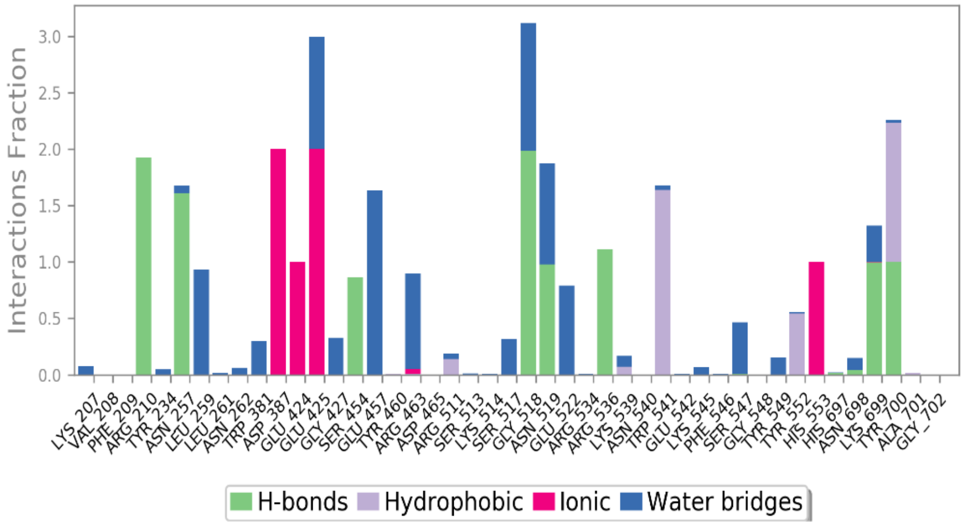

Trp541 changed orientation after 7 ns and does not go back to the flat conformation. The aromatic ring was stabilized by cation-π interactions with Arg511 in the first 25 ns, then it is exposed to the solvent without specific interactions. In the last 25 ns, the diethylene glycol chain folded and the aromatic ring formed π-π interactions with 1,2,3-triazole.

Ab initio calculations

The models were constructed using the software Maestro.[1] Geometries were initially optimized with MacroModel (Force Field: OPLS3,[14] vacuum, Method: PRCG). Afterwards, the geometries were further optimized at the M06-2X-D3/6-311G**++ level, in vacuo, with Ultrafine accuracy, 100 max iterations, tight convergence criteria for SCF (1e-6 energy change, 1e-7 density matrix change), tight convergence criteria (iaccg=5 in the input file) and the option “Switch to analytic integrals near convergence” on. Single point energies were calculated at the same level of theory and with the same options. Frequency analysis showed zero imaginary frequencies for all the optimized structures. Electrostatic potential surfaces of the fragments were generated by mapping the electrostatic potentials onto surfaces of molecular electron density (0.001 electron/Å) and rainbow color-coding, using the software Maestro.[1] The potential energy values range from +25 kcal/mol to -25 kcal/mol, where red signifies the maximum in negative potential and violet signifies the maximum in positive potential.

The dinitrophenyl (DNP) ring featured in ARM-P2 is electron-deficient, therefore face-centered stacking was favored with an electron-rich aromatic as indole,[23] whose negative electron potential was localized on top of the six-membered ring. For the electron-rich aromatic ring of **F-PSMA-MIC02** and **F-PSMA-MIC04** (Figure S19 B), face-centered stacking was disfavored.

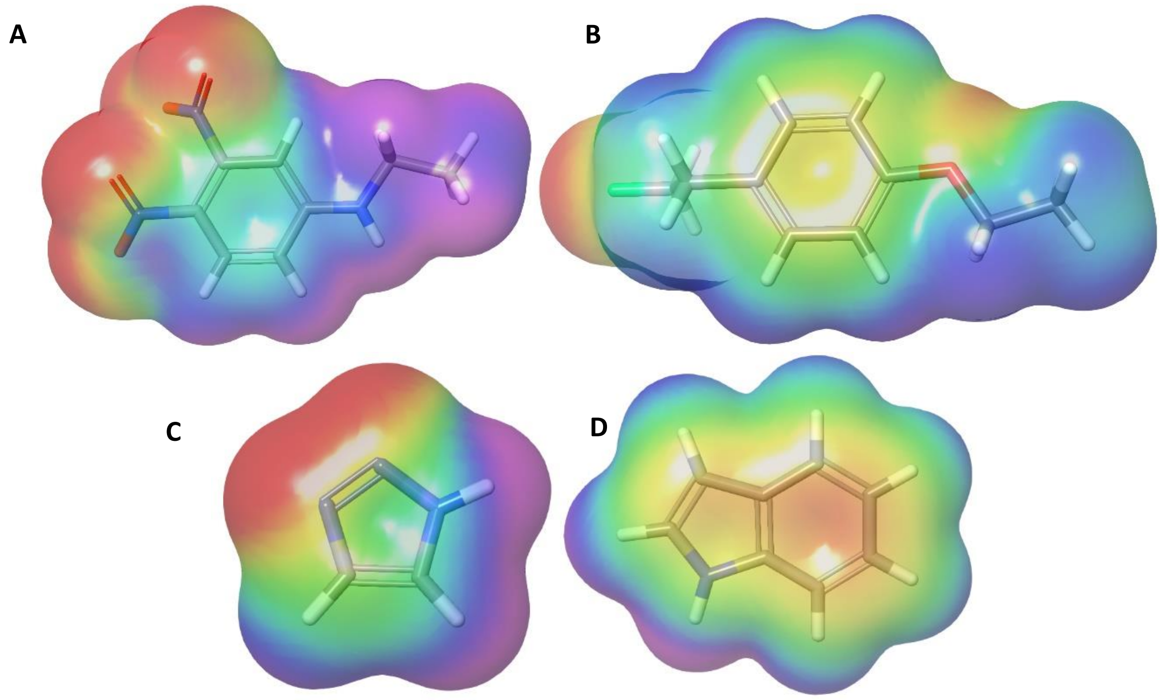

Figure S20. Electrostatic potential (ESP) surfaces for the fragments involved in the π-π interactions: (A) dinitrophenyl (DNP), (B) the aromatic ring featured in F-PSMA-

MIC02 and F-PSMA-MIC04, (C) [1,2,3]triazole and (D) indole. From negative to positive ESP values: red, yellow, green, blue, violet.

On the contrary, edge-to-face interactions were more favorable between two electron-rich aromatics, as predicted by our MD simulations for the aromatic ring and Trp541 (Figure 5 in the main text and Figures S14-S17).

1,2,3-triazole also showed a slightly positive electrostatic potential on top of the ring, which is in agreement with the occasional face-to-face π-π stacking that is observed with Trp541.

9. References

[1] Schrödinger Release 2019-4: Maestro, Schrödinger, LLC, New York, NY, 2019

[2] M. Felber, M. Bauwens, J. M. Mateos, S. Imstepf, F. M. Mottaghy, R. Alberto, *Chemistry* **2015**, *21*, 6090–6099.

[3] J. Rokka, A. Snellman, C. Zona, B. La Ferla, F. Nicotra, M. Salmona, G. Forloni, M. Haaparanta-Solin, J. O. Rinne, O. Solin, *Bioorganic Med. Chem.* **2014**, *22*, 2753–2762.

[4] M. Wang, C. D. McNitt, H. Wang, X. Ma, S. M. Scarry, Z. Wu, V. V Popik, Z. Li, *Chem. Commun.* **2018**, *54*, 7810–7813.

[5] K. Sakurai, T. M. Snyder, D. R. Liu, *J. Am. Chem. Soc.* **2005**, *127*, 1660–1661.

[6] R. Yan, E. El-Emir, V. Rajkumar, M. Robson, A. P. Jathoul, R. B. Pedley, E. Årstad, *Angew. Chemie Int. Ed.* **2011**, *50*, 6793–6795.

[7] D. J. Leaver, R. M. Dawson, J. M. White, A. Polyzos, A. B. Hughes, *Org. Biomol. Chem.* **2011**, *9*, 8465.

[8] V. Percec, P. Leowanawat, H.-J. Sun, O. Kulikov, C. D. Nusbaum, T. M. Tran, A. Bertin, D. A. Wilson, M. Peterca, S. Zhang, et al., *J. Am. Chem. Soc.* **2013**, *135*, 9055–9077.

[9] J. Diot, M. I. García-Moreno, S. G. Gouin, C. Ortiz Mellet, K. Haupt, J. Kovensky, *Org. Biomol. Chem.* **2009**, *7*, 357–363.

[10] U. Sirion, H. J. Kim, J. H. Lee, J. W. Seo, B. S. Lee, S. J. Lee, S. J. Oh, D. Y. Chi, *Tetrahedron Lett.* **2007**, *48*, 3953–3957.

[11] H. H. Coenen, A. D. Gee, M. Adam, G. Antoni, C. S. Cutler, Y. Fujibayashi, J. M. Jeong, R. H. Mach, T. L. Mindt, V. W. Pike, A. D. Windhorst, *Nucl. Med. Biol.* **2017**, *55*, v–xi.

[12] E. Harder, W. Damm, J. Maple, C. Wu, M. Reboul, J. Y. Xiang, L. Wang, D. Lupyan, M. K. Dahlgren, J. L. Knight, J.W. Kaus, D. S. Cerutti, G. Krilov, W. Jorgensen, R. Abel, R. A. Friesner, *J. Chem. Theory Comput.* **2016**, *12*, 281–296.

[13] A. X. Zhang, R. P. Murelli, C. Barinka, J. Michel, A. Cocleaza, W. L. Jorgensen, J. Lubkowski, D. A. Spiegel, *J. Am. Chem. Soc.* **2010**, *132*, 12711–12716.

[14] R. A. Friesner, R. B. Murphy, M. P. Repasky, L. L. Frye, J. R. Greenwood, T. A. Halgren, P. C. Sanschagrin, D. T. Mainz, *J. Med. Chem.* **2006**, *49*, 6177–6196.

[15] G. Marcou, D. Rognan, *J. Chem. Inf. Model.* **2007**, *47*, 195–207.

[16] W. L. Jorgensen, J. Chandrasekhar, J. D. Madura, R. W. Impey, M. L. Klein, *J. Chem. Phys.* **1983**, *79*, 926–935.

[17] K. J. Bowers, E. Chow, H. Xu, R. O. Dror, M. P. Eastwood, B. A. Gregersen, J. L. Klepeis, I. Kolossvary, M. A. Moraes, F. D. Sacerdoti, J. K. Salomon, Y. Shan, D.E. Shaw, in *Proc. 2006 ACM/IEEE Conf. Supercomput.*, Association For Computing Machinery, New York, NY, USA, **2006**, pp. 84–es.

[20] H. J. C. Berendsen, J. P. M. Postma, W. F. van Gunsteren, A. DiNola, J. R. Haak, *J. Chem. Phys.* **1984**, *81*, 3684–3690.

[21] G. J. Martyna, D. J. Tobias, M. L. Klein, *J. Chem. Phys.* **1994**, *101*, 4177–4189.

[22] J. C. Evans, M. Malhotra, J. F. Cryan, C. M. O'Driscoll, *Br. J. Pharmacol.* **2016**, DOI 10.1111/bph.13576.

[23] C.R. Martinez, B. L. Iverson *Chem. Sci.* **2012**, *3*, 2191-2201

NMR and Mass spectra

10. Synthesis of F-PSMA-MIC01

10.1. (9*S*,13*S*)-Tri-tert-butyl 3,11-dioxo-1-phenyl-2-oxa-4,10,12-triazapentadecane-9,13,15-tricarboxylate (2).

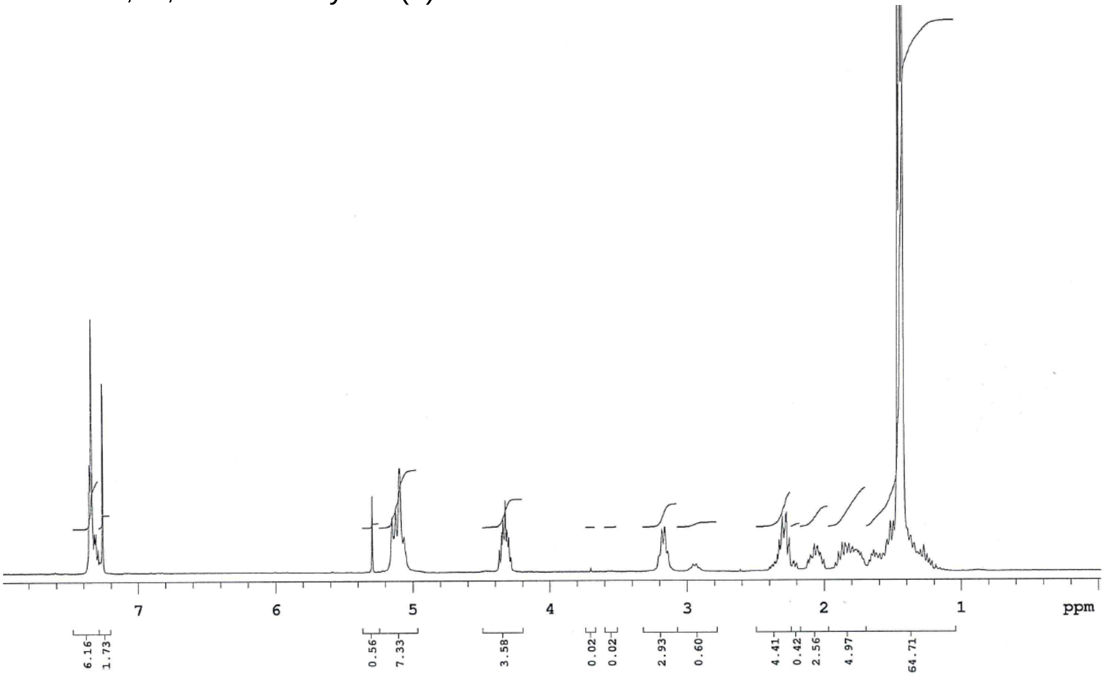

Figure S21: ^{1}H NMR

10.2. Di-*tert*-butyl (((*S*)-6-amino-1-(*tert*-butoxy)-1-oxohexan-2-yl)carbamoyl)-*L*-glutamate (3).

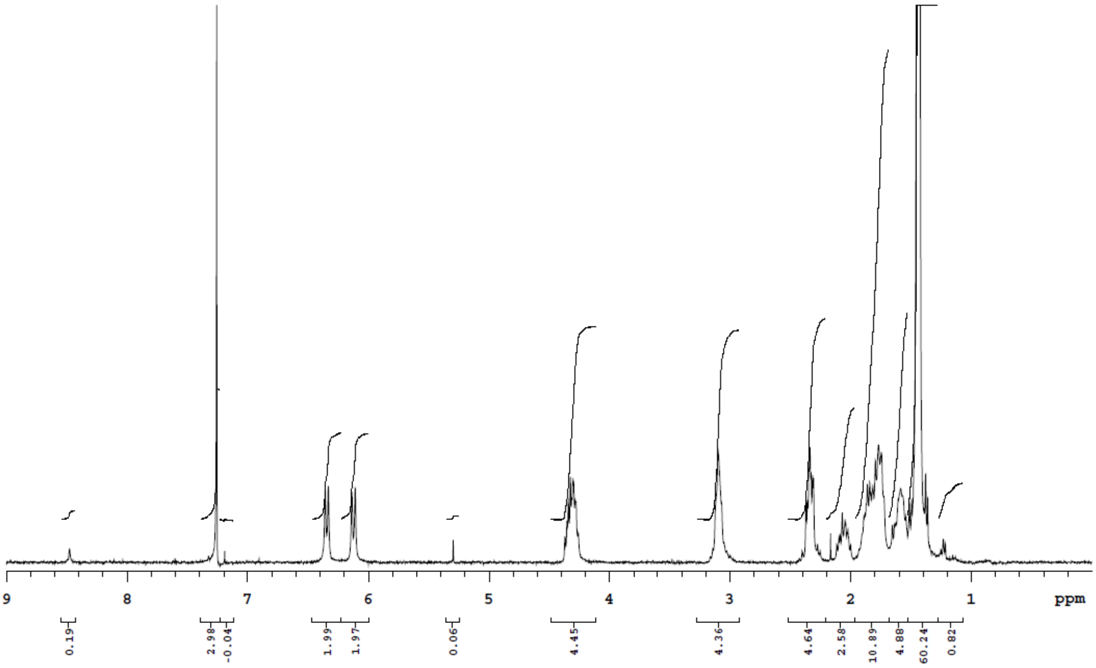

Figure S22. ^{1}H NMR

10.3. 2,5-Dioxopyrrolidin-1-yl 4-((trimethylsilyl)ethynyl)benzoate (5).

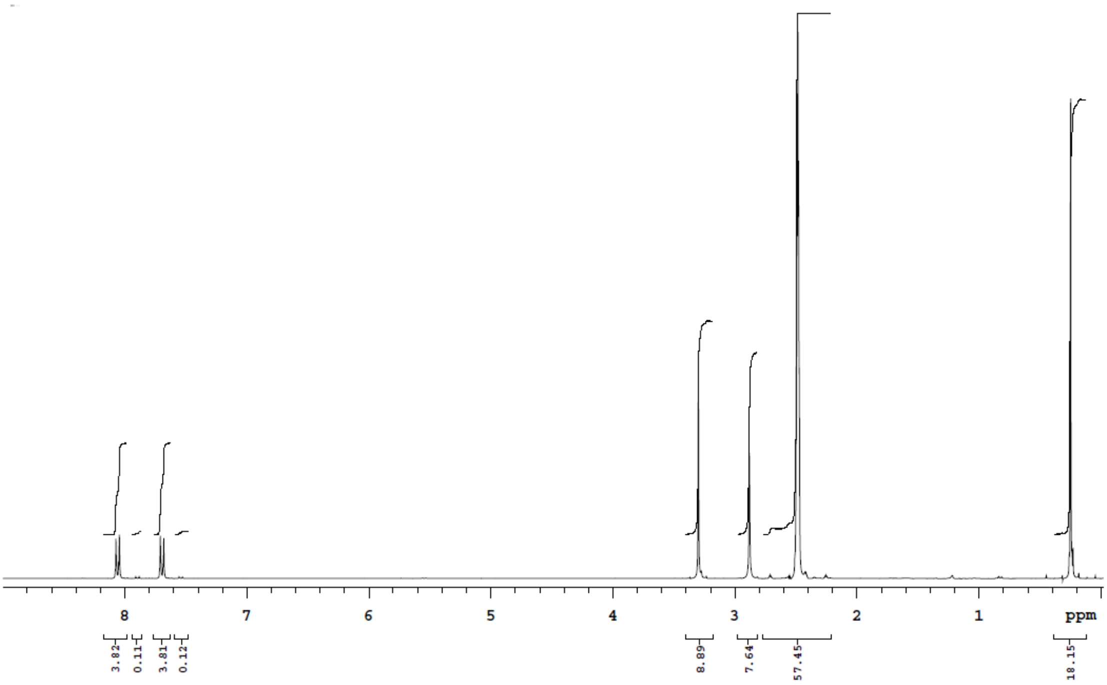

Figure S23. ^{1}**H NMR**

10.4. Di-*tert*-butyl (((S)-1-(tert-butoxy)-1-oxo-6-(4-((trimethylsilyl)ethynyl)benzamido)hexan-2-yl)carbamoyl)-l-glutamate (6).

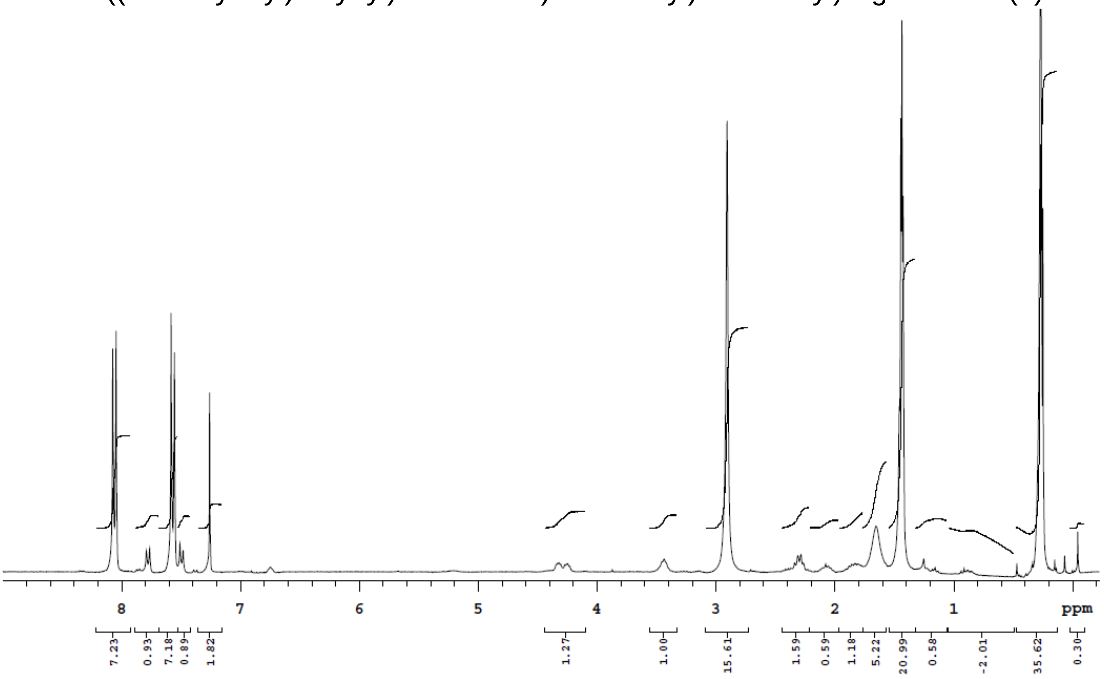

Figure S24. ^{1}**H NMR**

10.5. (((*S*)-1-Carboxy-5-(4-ethynylbenzamido)pentyl)carbamoyl)-*L*-glutamic acid (7).

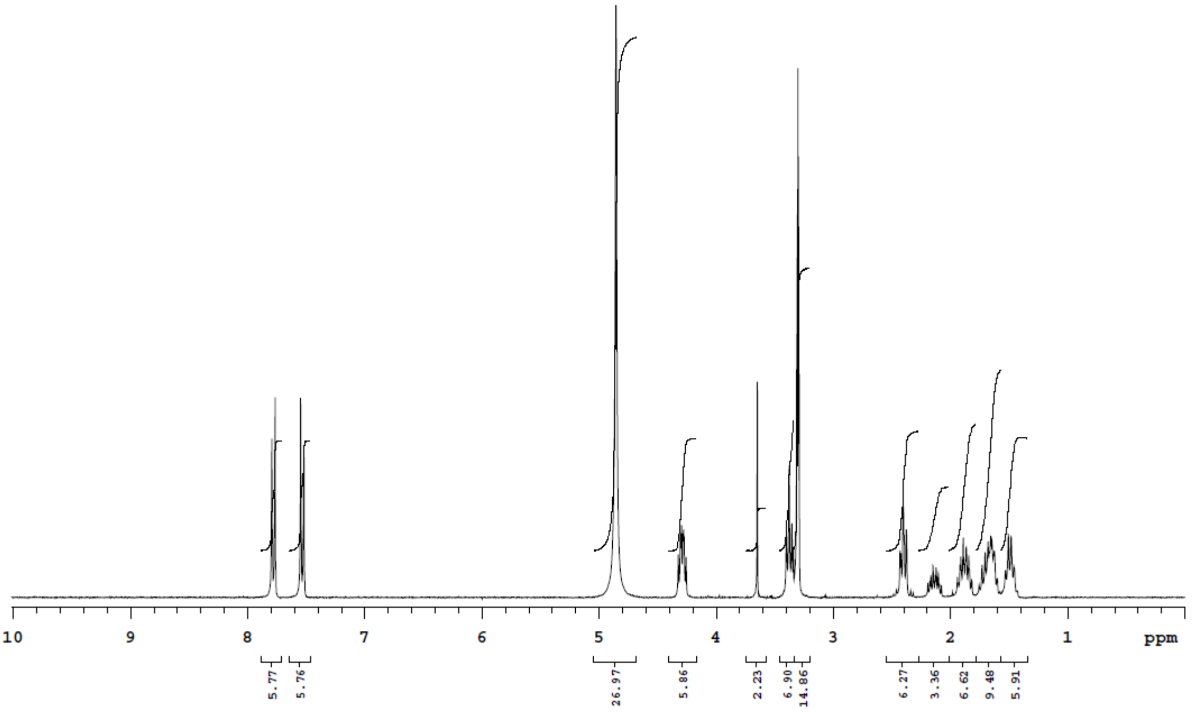

FigureS25. ^{1}H NMR

10.6. 2-(2-(2-azidoethoxy)ethoxy)ethoxy)ethyl-4-methylbenzene-1-sulfonate (8).

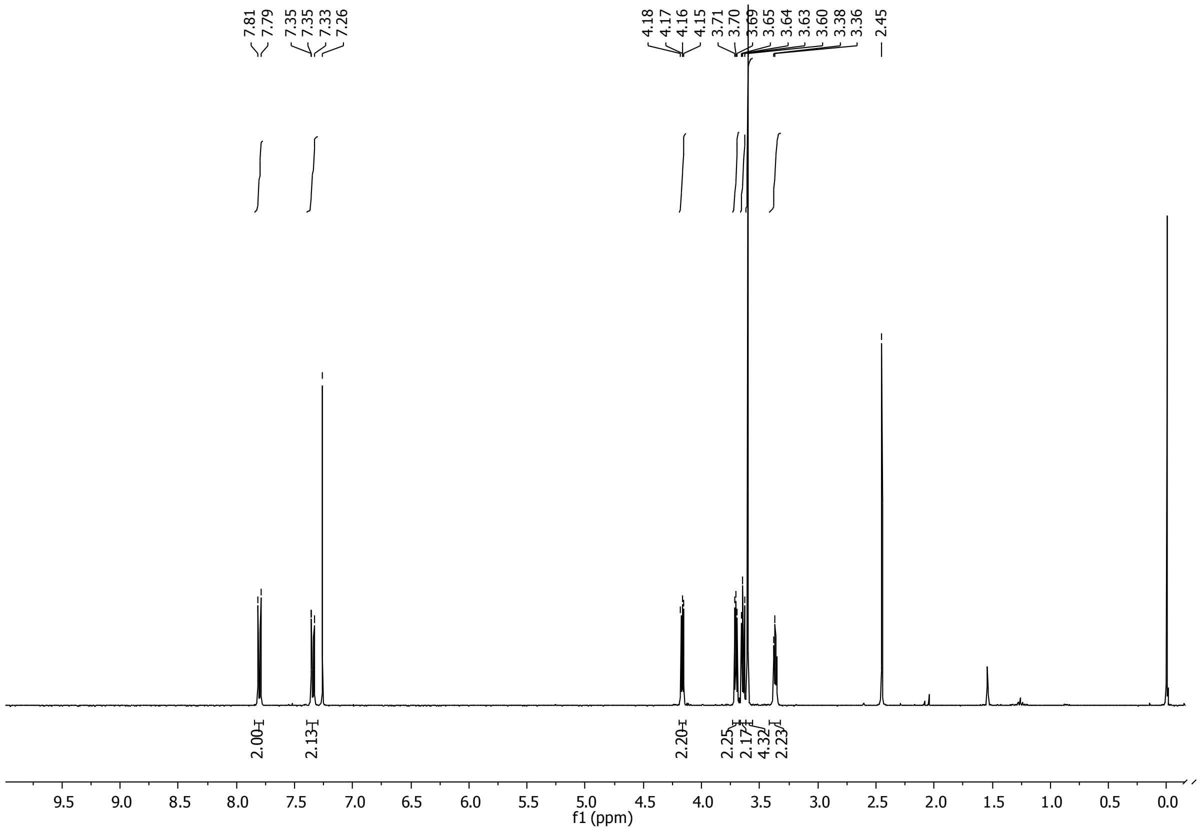

Figure S26. ^{1}H NMR

10.7. 1-Azido-2-(2-(2-fluoroethoxy)ethoxy)ethane (9).

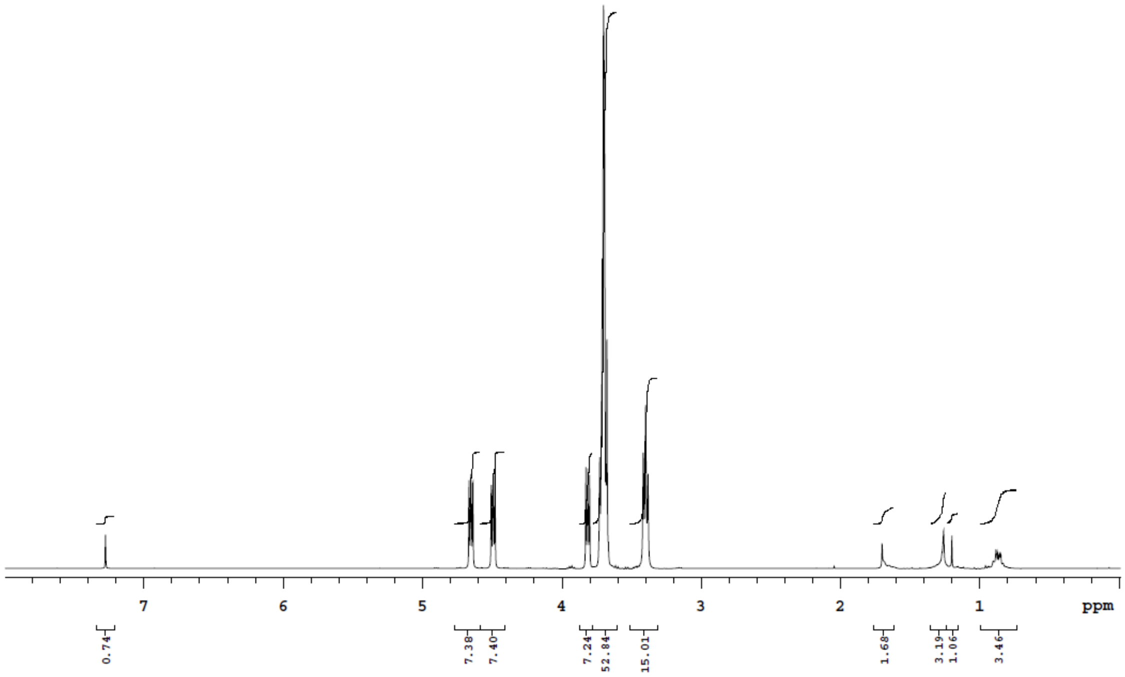

Figure S27. [1]**H NMR**

10.8. (((S)-1-Carboxy-5-(4-(1-(2-(2-(2-fluoroethoxy)ethoxy)ethyl)-1*H*-1,2,3-triazol-4-yl)benzamido)pentyl)carbamoyl)-*L*-glutamic acid (F-PSMA-MIC01).

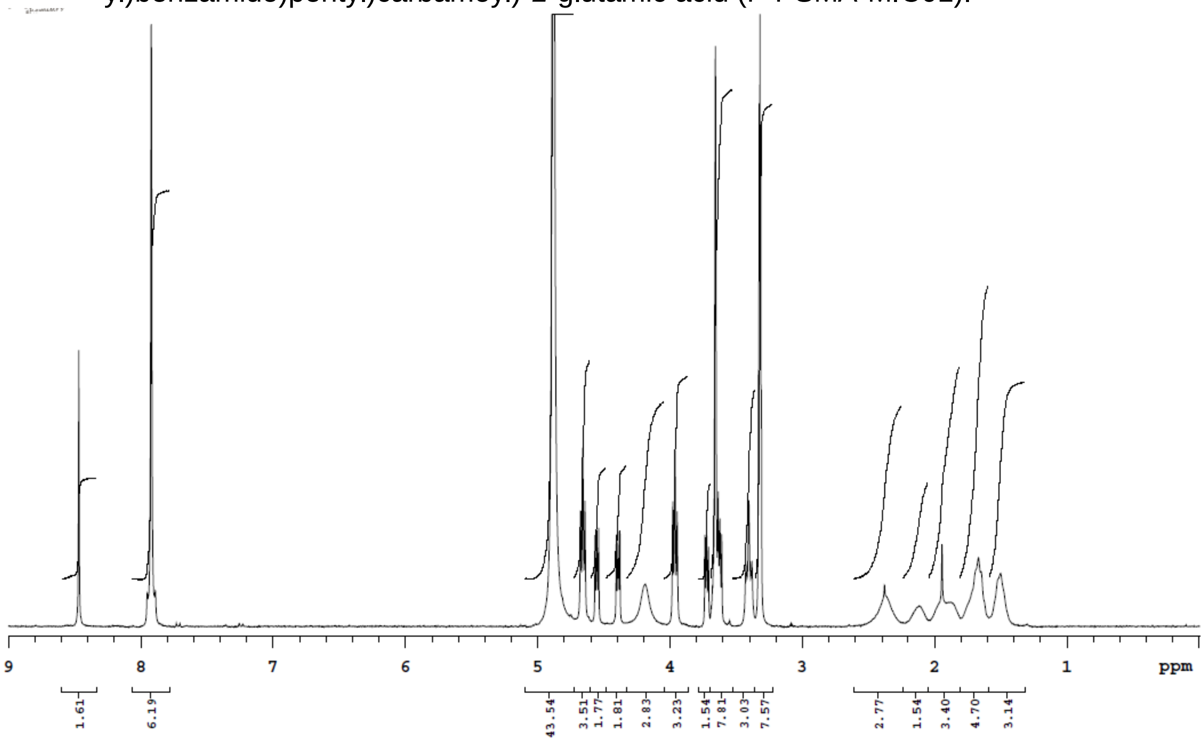

Figure S28. [1]**H NMR**

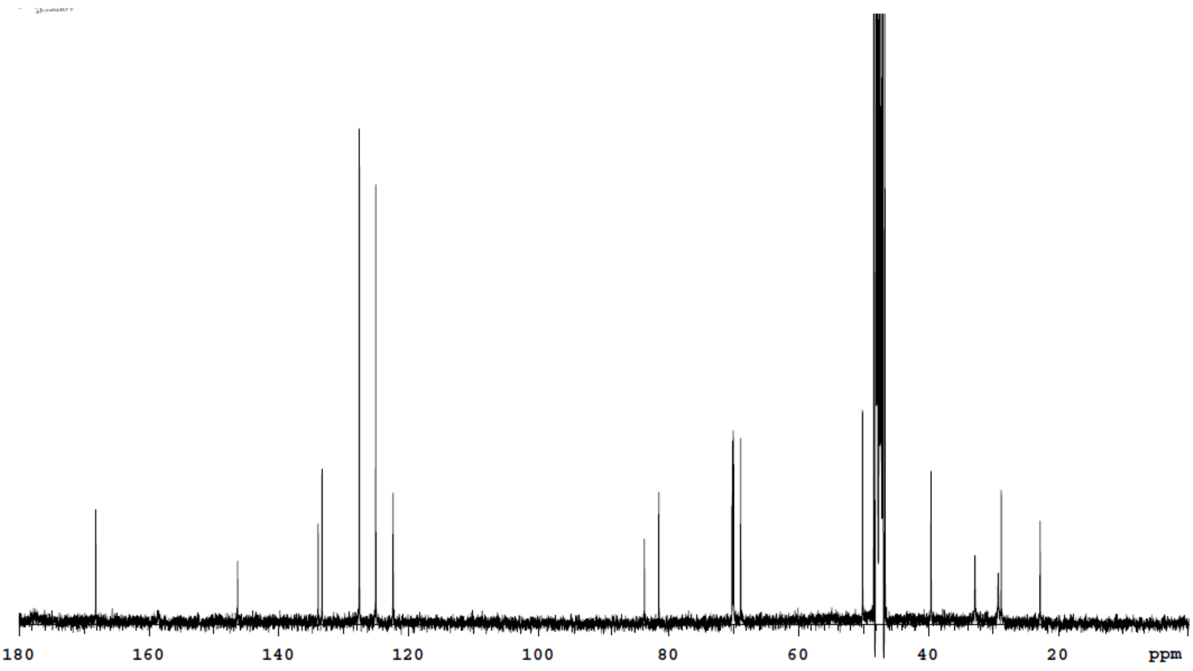

Figure S29. ^{13}C NMR

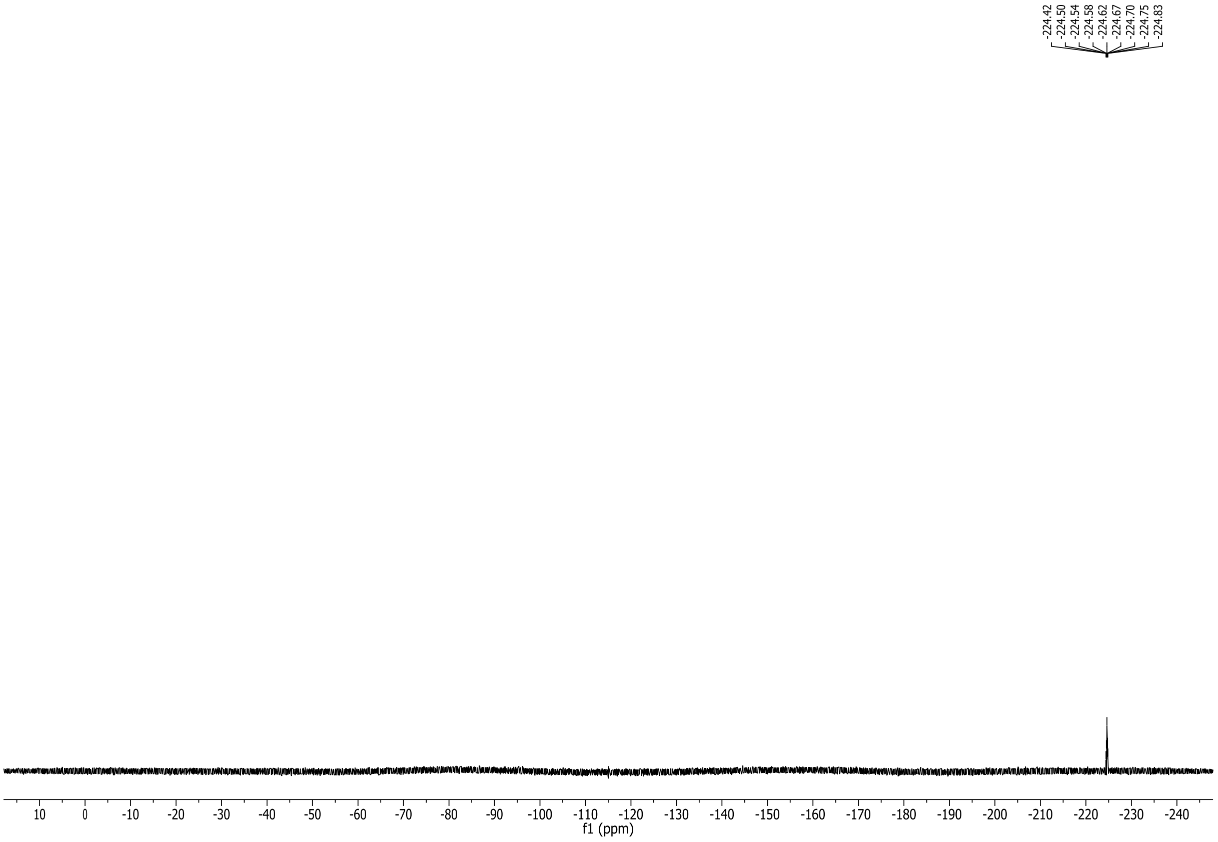

Figure S30. ^{19}F NMR

11. Synthesis of F-PSMA-MIC02

11.1. 2-(4-(2-(2-(2-azidoethoxy)ethoxy)ethoxy)phenyl)-ethan-1-ol (15).

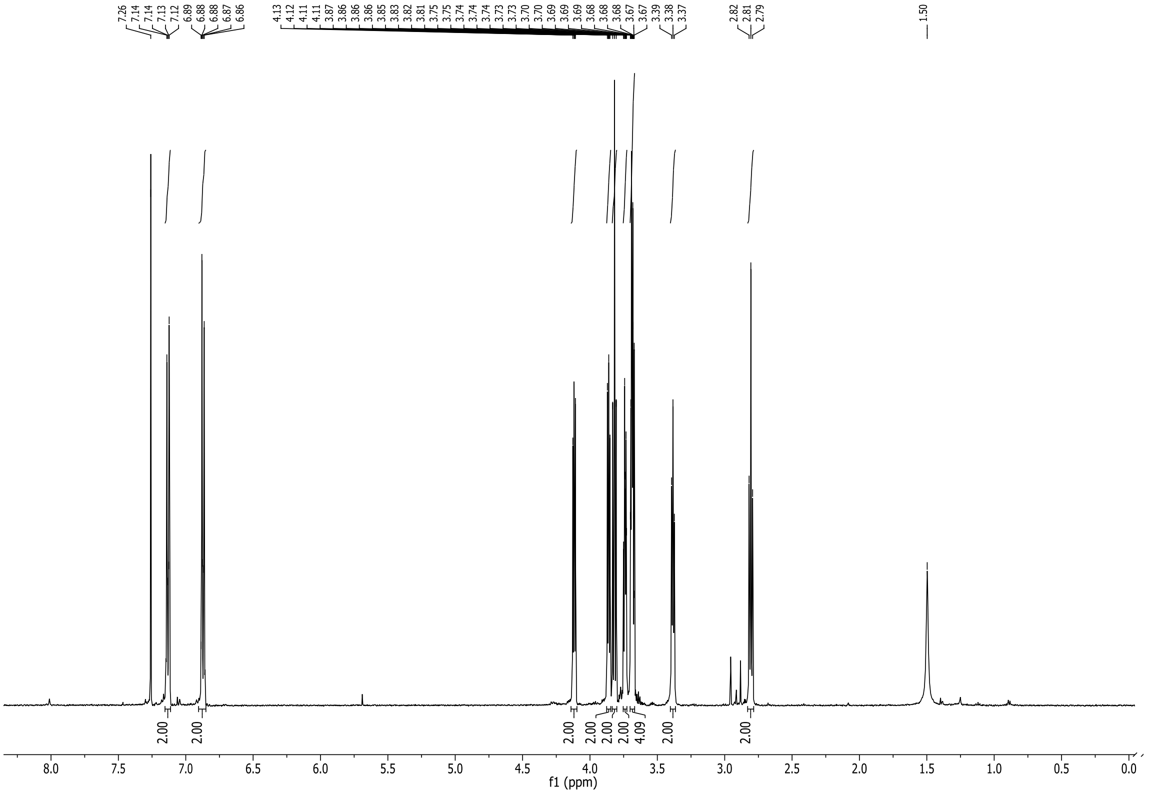

Figure S31. ^{1}**H NMR**

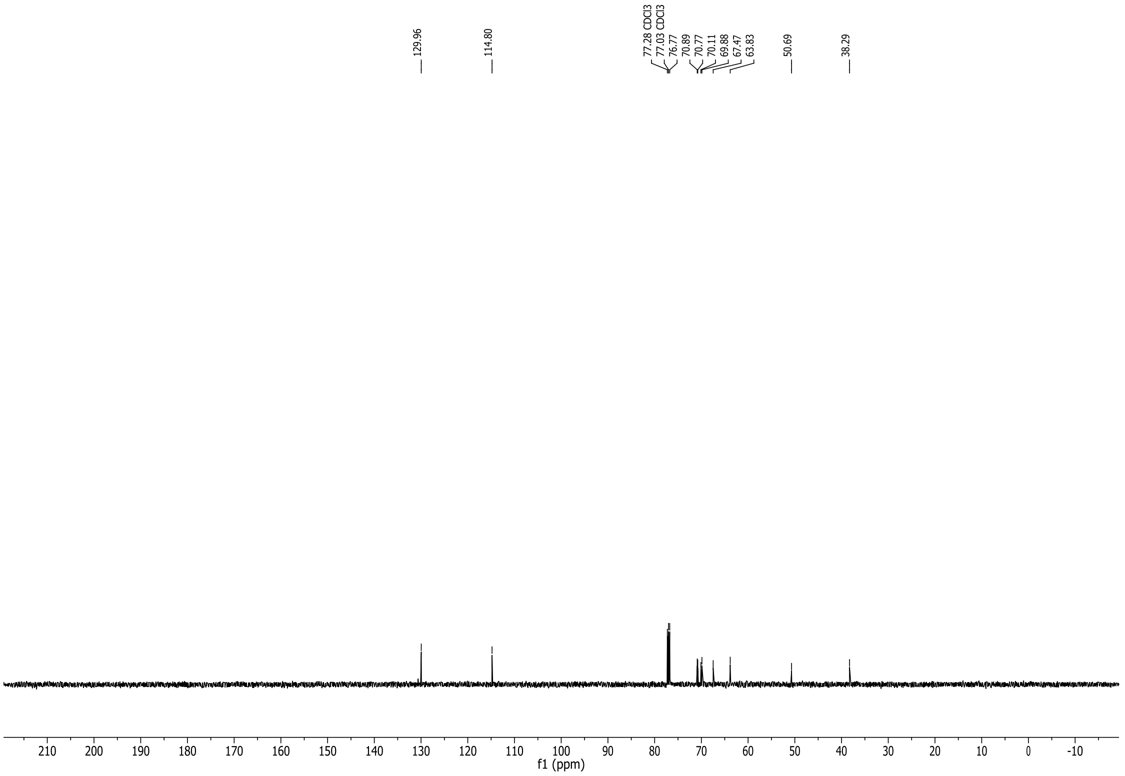

FigureS32. ^{11}**C NMR**

12. 2-(4-(2-(2-(2-azidoethoxy)ethoxy)ethoxy)phenyl)ethyl-4-methylbenzene-1-sulfonate (16).

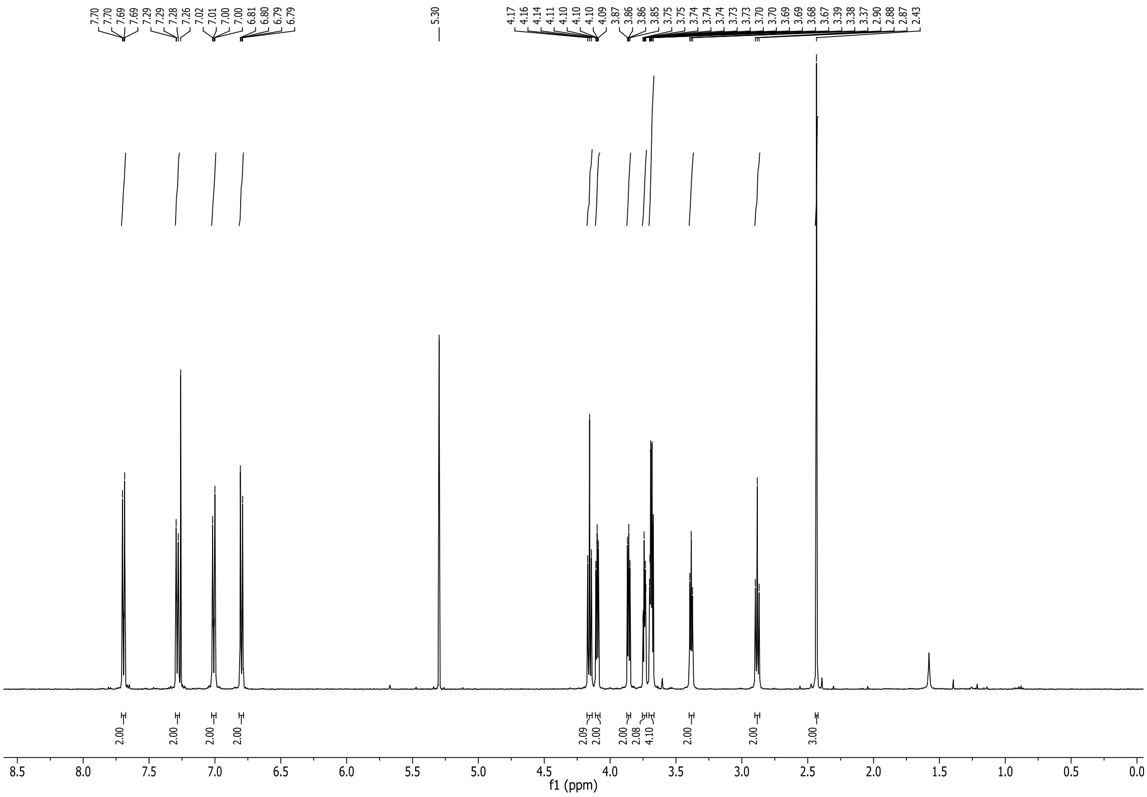

FigureS33. ^{1}H NMR

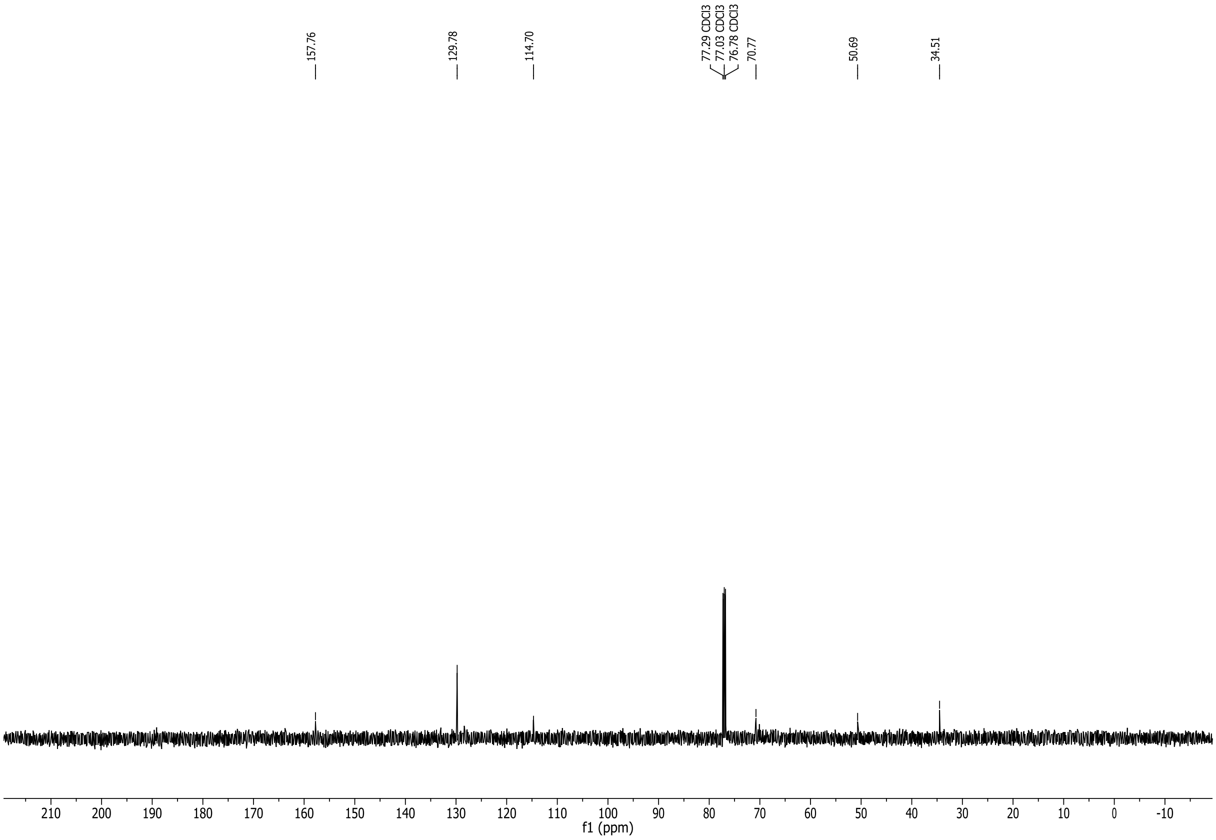

FigureS34. ^{11}C NMR

12.1. 1-(2-(2-(2-azidoethoxy)ethoxy)ethoxy)-4-(2-fluoroethyl)benzene (17).

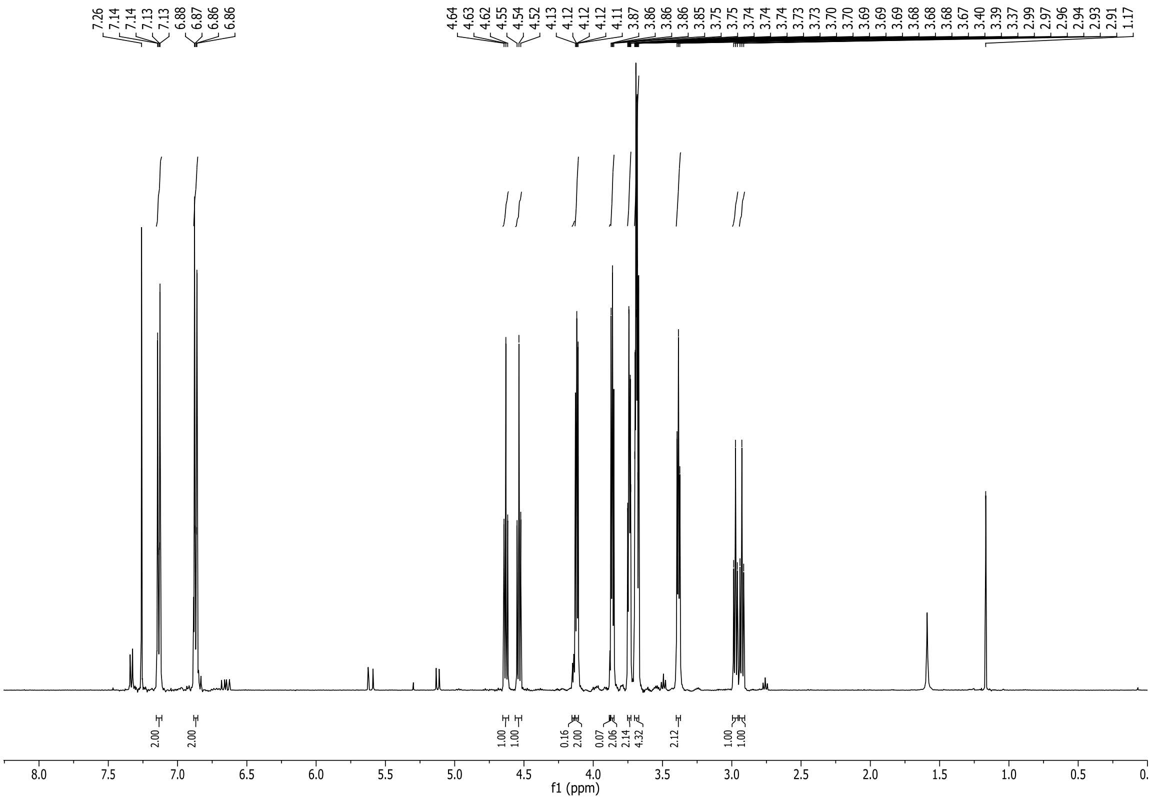

FigureS35. ^{1}H NMR

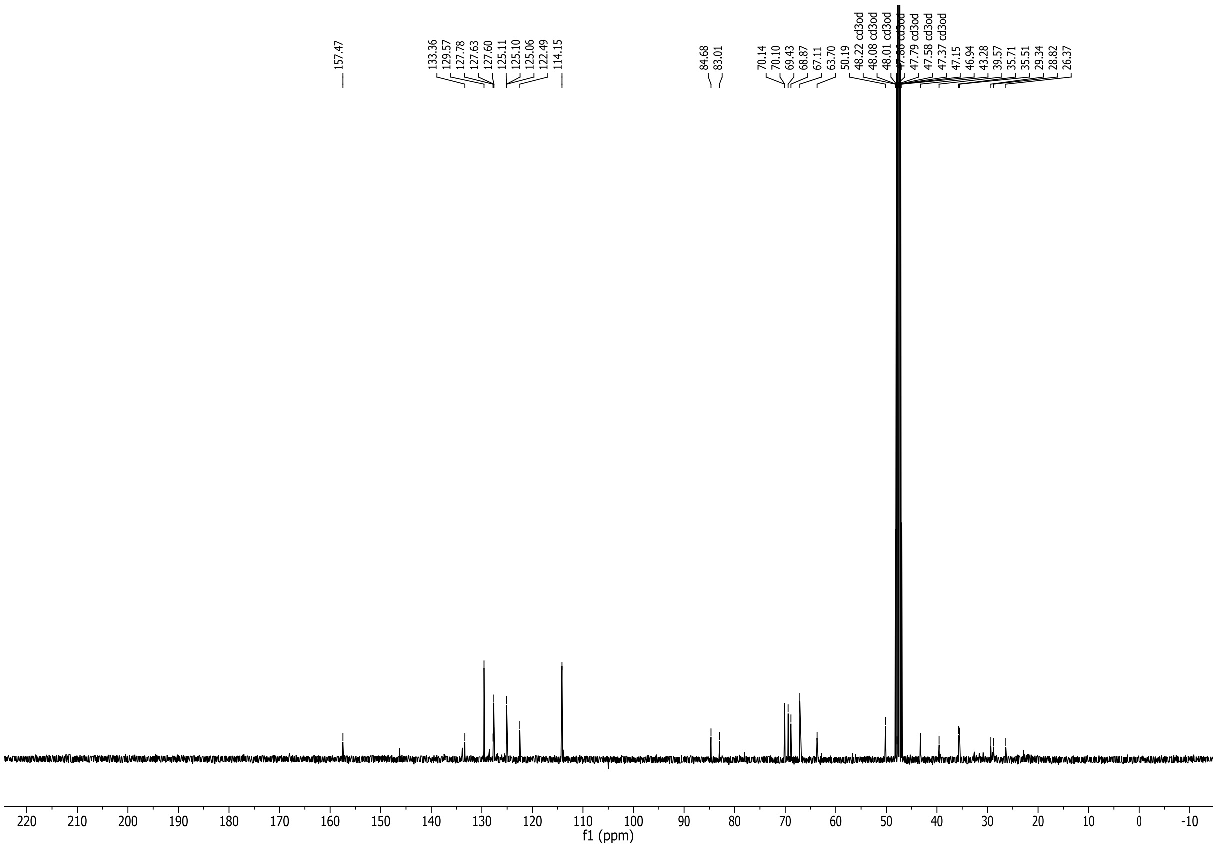

FigureS36. ^{11}C NMR

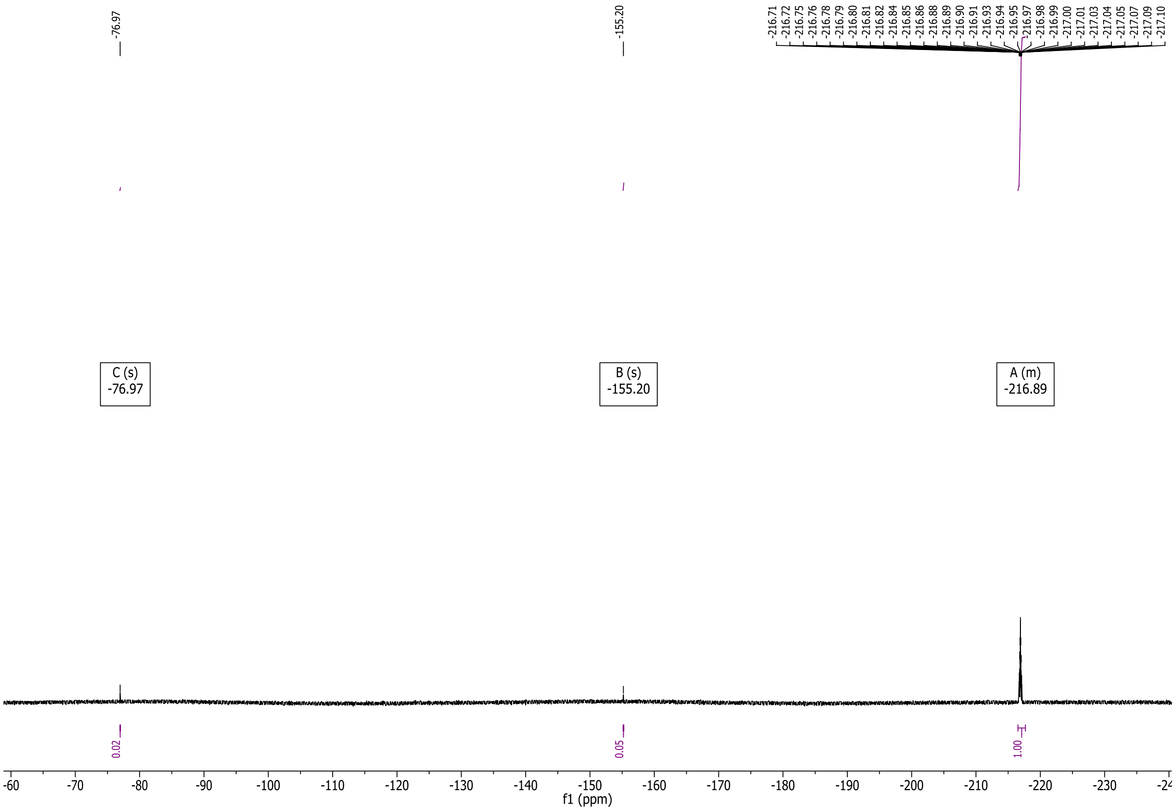

Figure S37. ^{19}**F NMR**

12.2. 2-(4-(2-(2-(2-azidoethoxy)ethoxy)ethoxy)phenyl)ethyl-4-methylbenzene-1-sulfonate (F-PSMA-MIC02).

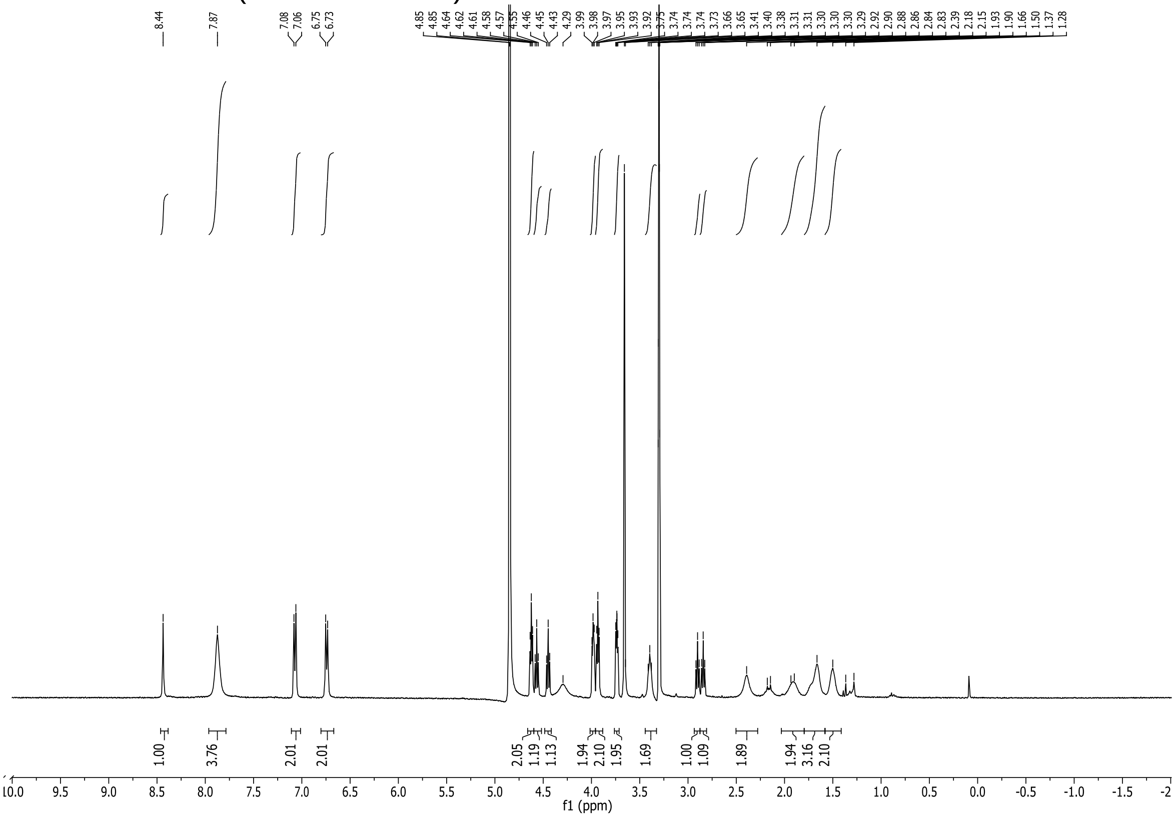

Figure S38. ^{1}**H NMR**

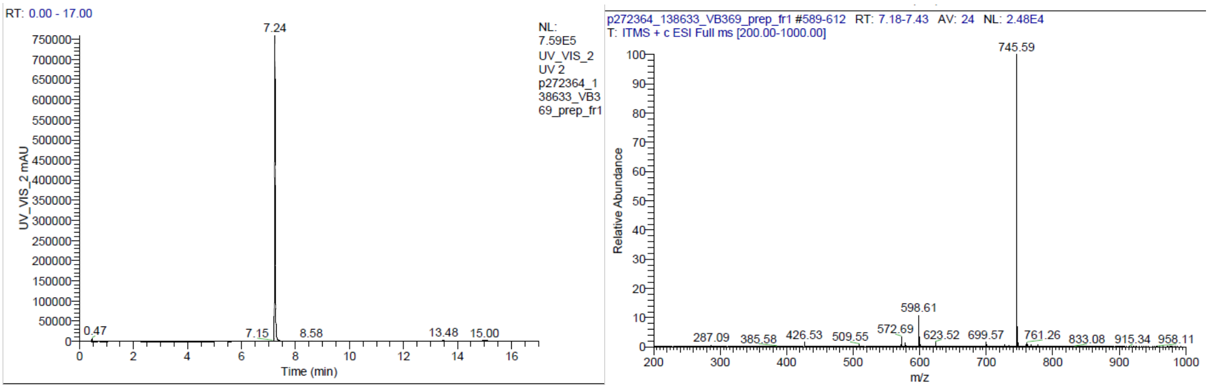

UPLC measurements were performed using the following setup: **Column:** ACQUITY UPLC® HSS T3 1.8µm, 2.1 x 150 mm; **Detection:** λ = 254 nm; **Flow:** 0.3 mL/min; **Eluent A:** 0.1% formic acid in water; **Eluent B:** 0.1% formic acid in acetonitrile; **Program:** (0-1 min) 5% B; (1-8 min) linear gradient to 90% B; (8-11 min) 90% B; (11-12 min) linear gradient to 5% B; (12-17 min) 5% B.

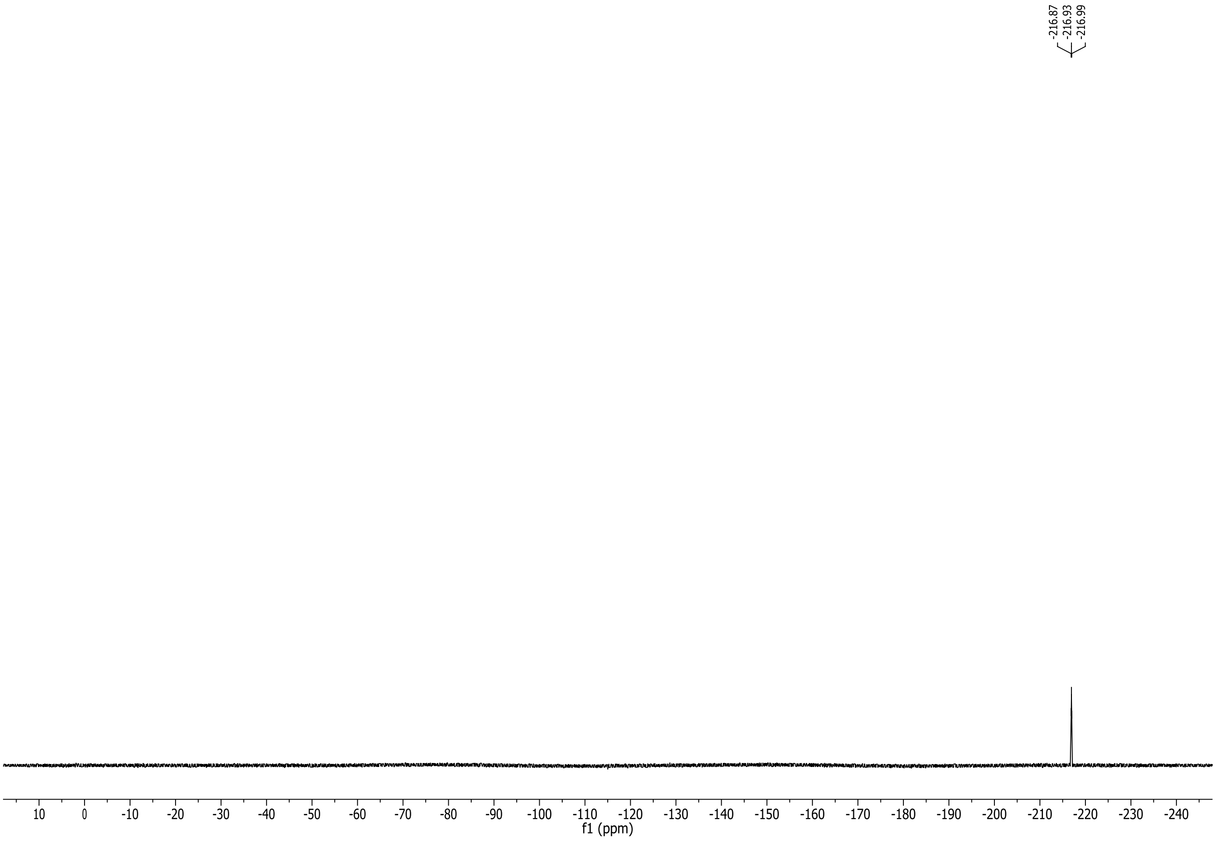

Figure S39. ^{19}**F NMR**

13. Synthesis of F-PSMA-MIC03

13.1. (((*S*)-5-Amino-1-carboxypentyl)carbamoyl)-*L*-glutamic acid (10).

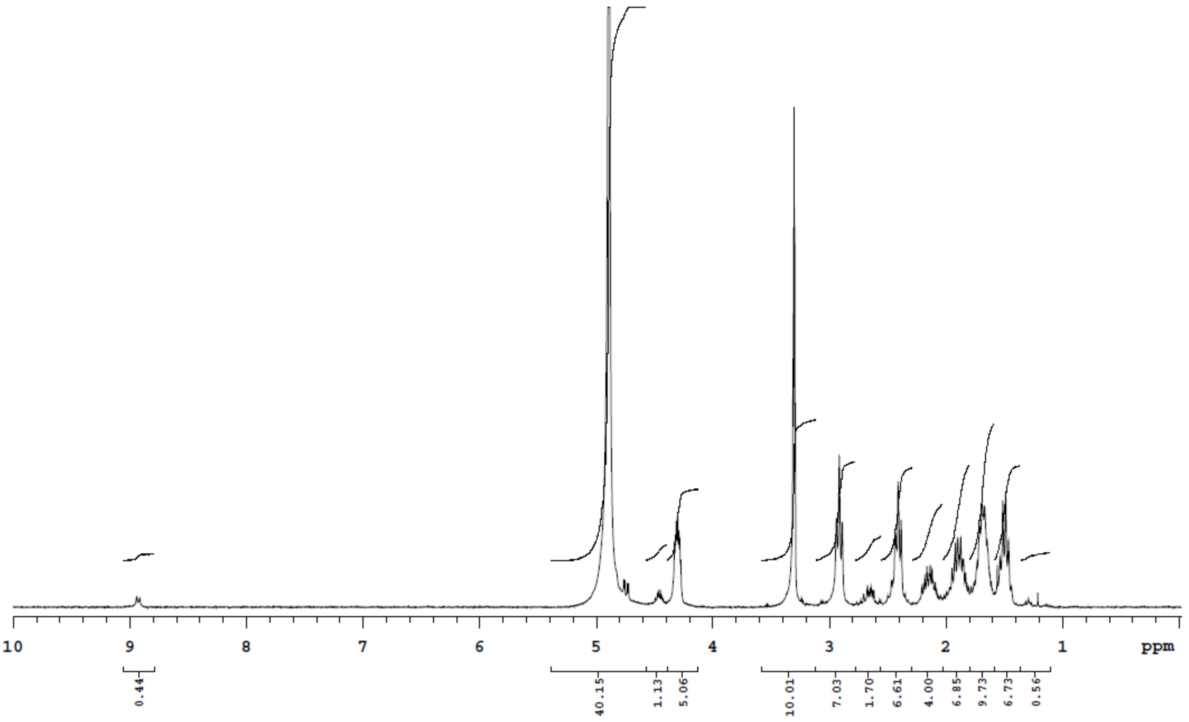

FigureS40. ^{1}H NMR

13.2. 4-Azidomethyl benzoic acid (12).

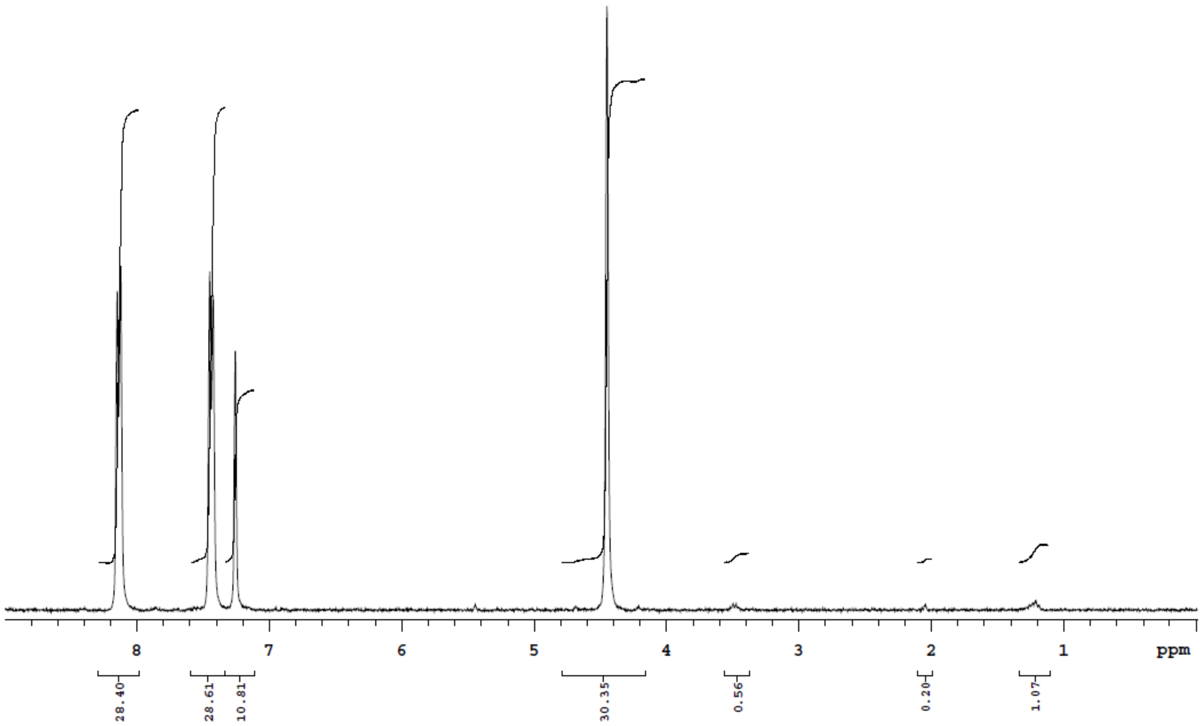

Figure S41. ^{1}H NMR

13.3. 2,5-Dioxopyrrolidin-1-yl 4-(azidomethyl)benzoate (13).

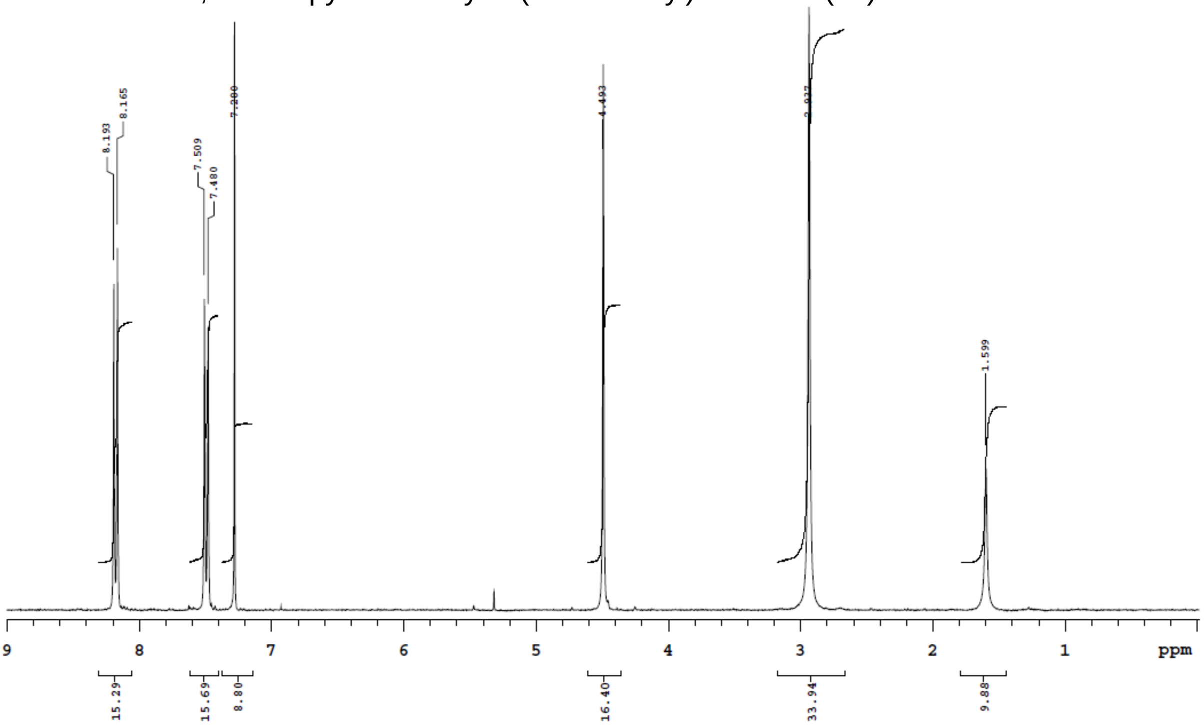

FigureS42. ^{1}H NMR

13.4. (((*S*)-5-(4-(azidomethyl)benzamido)-1-carboxypentyl)carbamoyl)-*L*-glutamic acid (14).

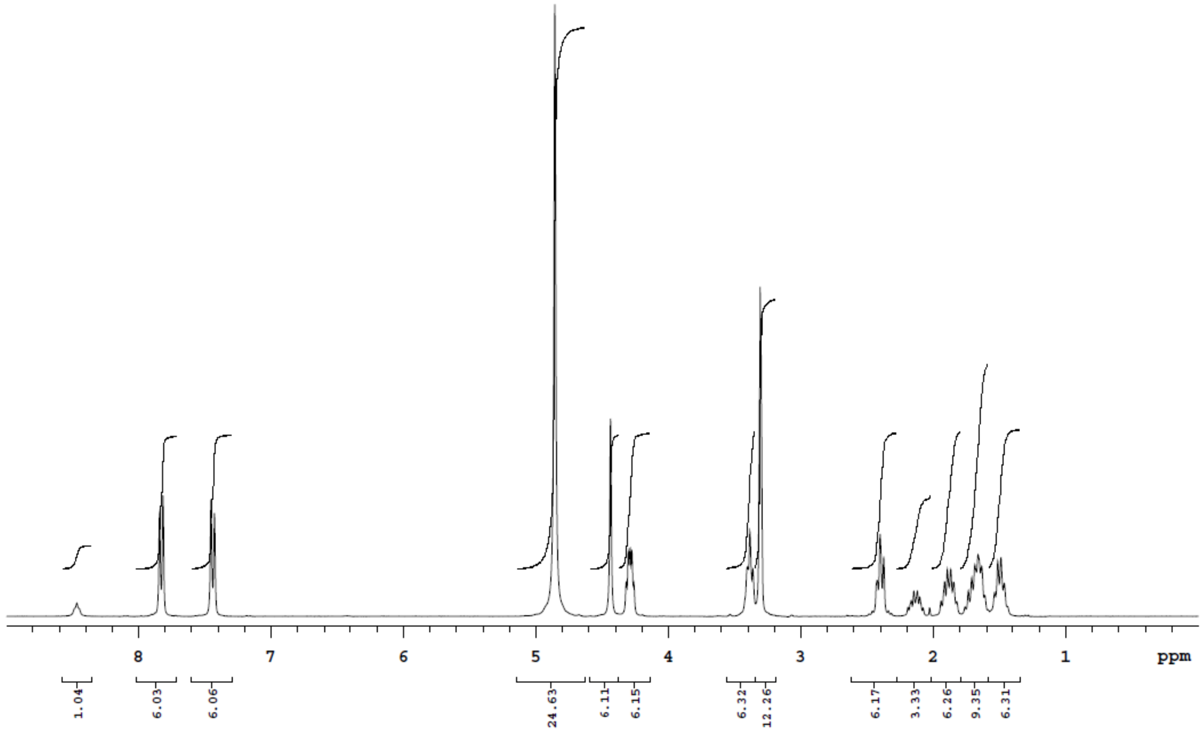

Figure S43. ^{1}H NMR

13.5. 2-(2-(prop-2-yn-1-yloxy)ethoxy)ethan-1-ol (S3)

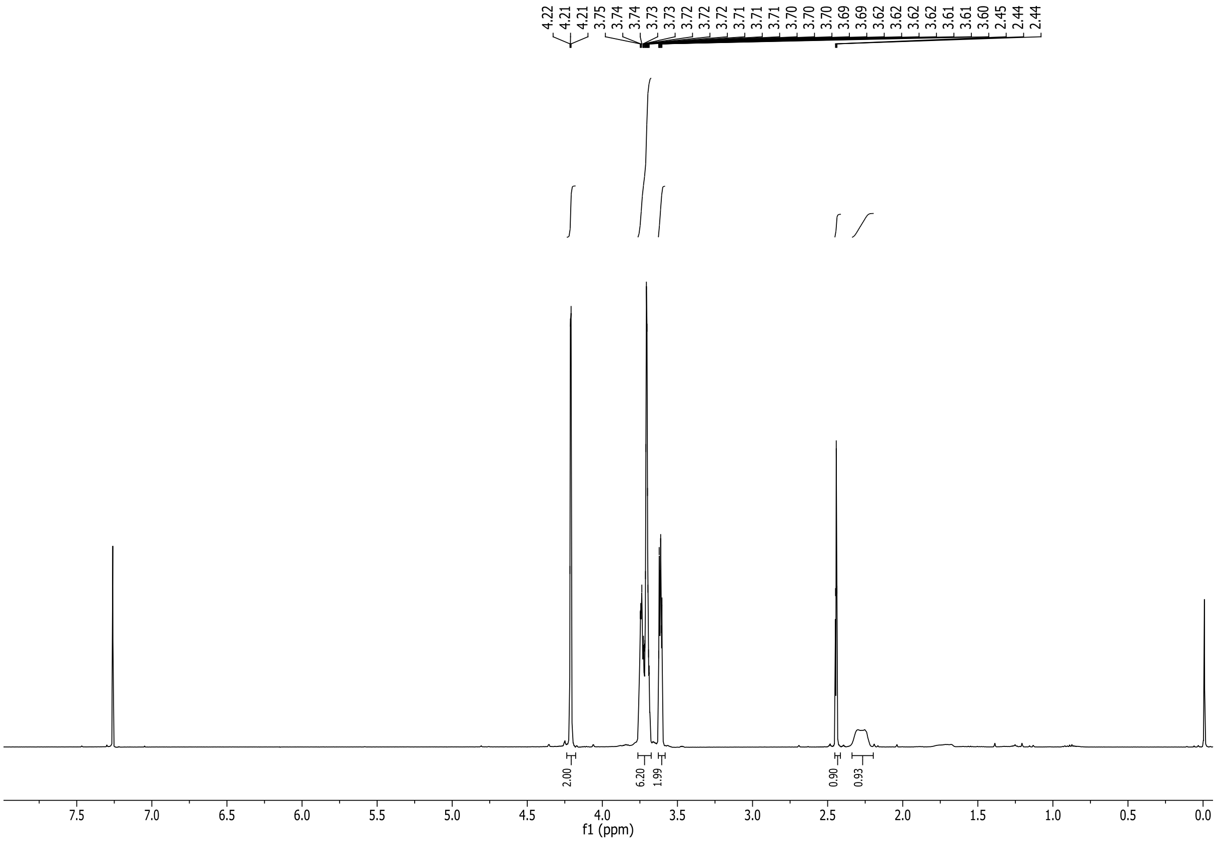

Figure S44. ^{1}**H NMR**

13.6. 2-(2-(prop-2-yn-1-yloxy)ethoxy)ethyl 4-methylbenzenesulfonate (18)

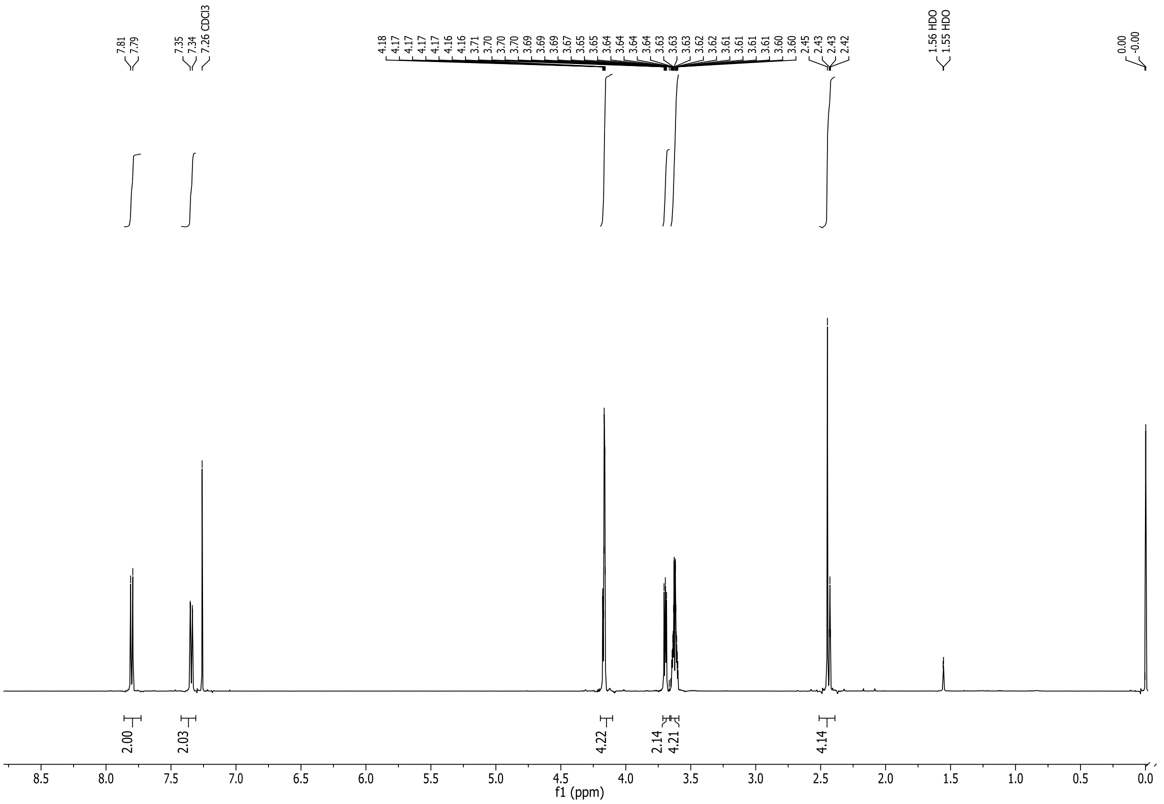

Figure S45. ^{1}**H NMR**

13.7. (((*S*)-1-carboxy-5-(4-((4-((2-(2-fluoroethoxy)ethoxy)methyl)-1*H*-1,2,3-triazol-1-yl)methyl)benzamido)pentyl)carbamoyl)-*L*-glutamic acid (F-PSMA-MIC03)

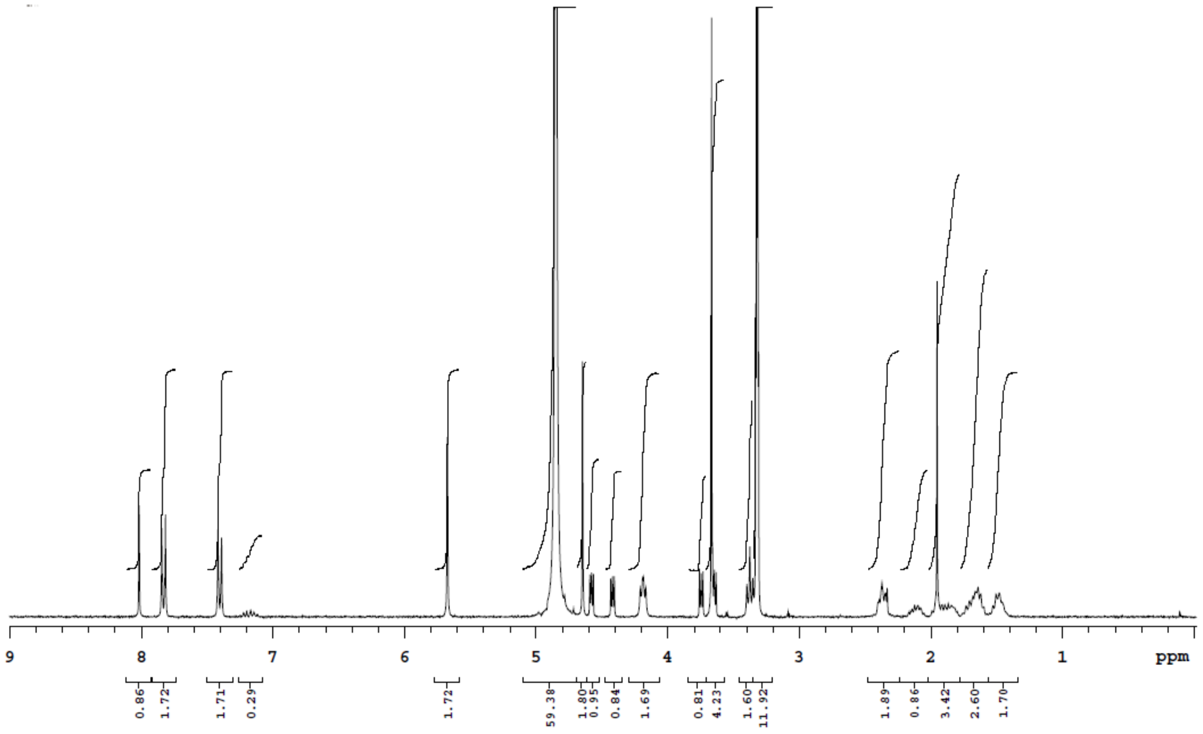

Figure S46. ^{1}**H NMR**

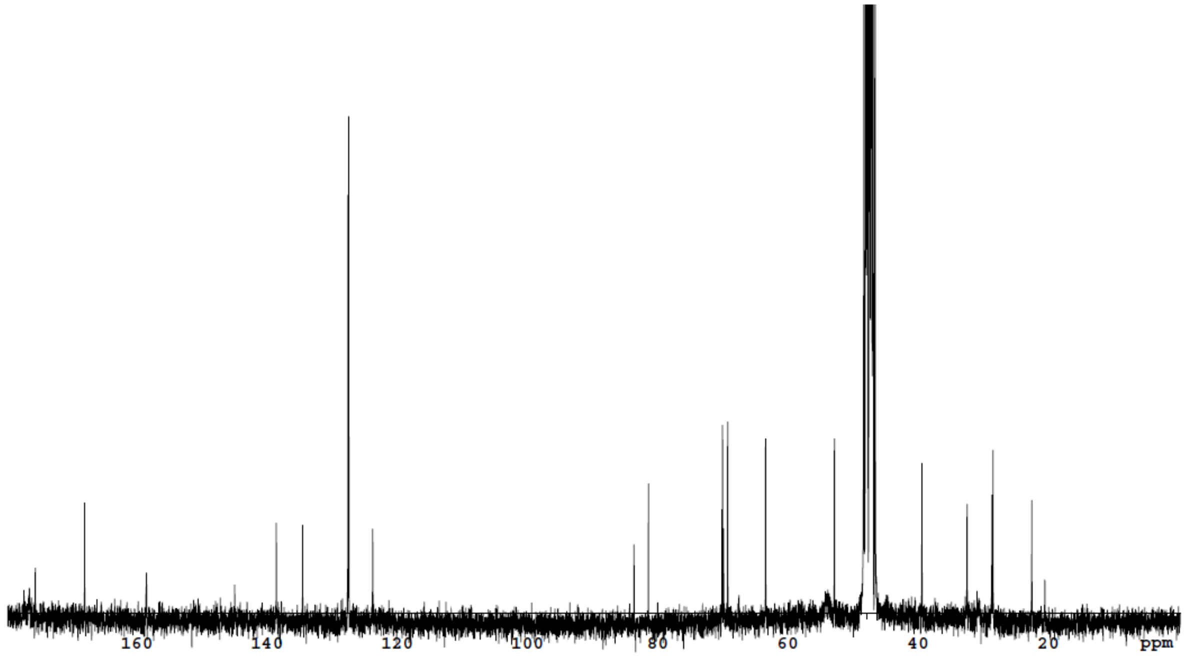

FigureS47. ^{11}**C NMR**

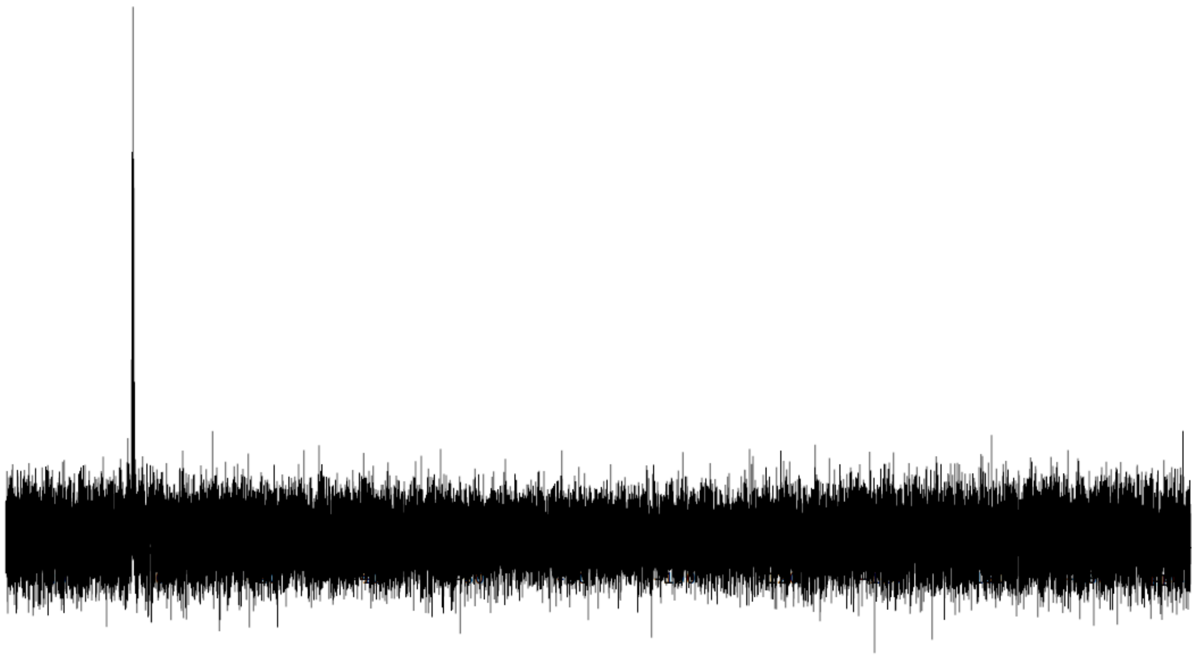

Figure S48. ^{19}**F NMR**

14. Synthesis of F-PSMA-MIC04

14.1. 2-(2-(2-(prop-2-yn-1-yloxy)-ethoxy)-phenyl)ethan-1-ol (19)

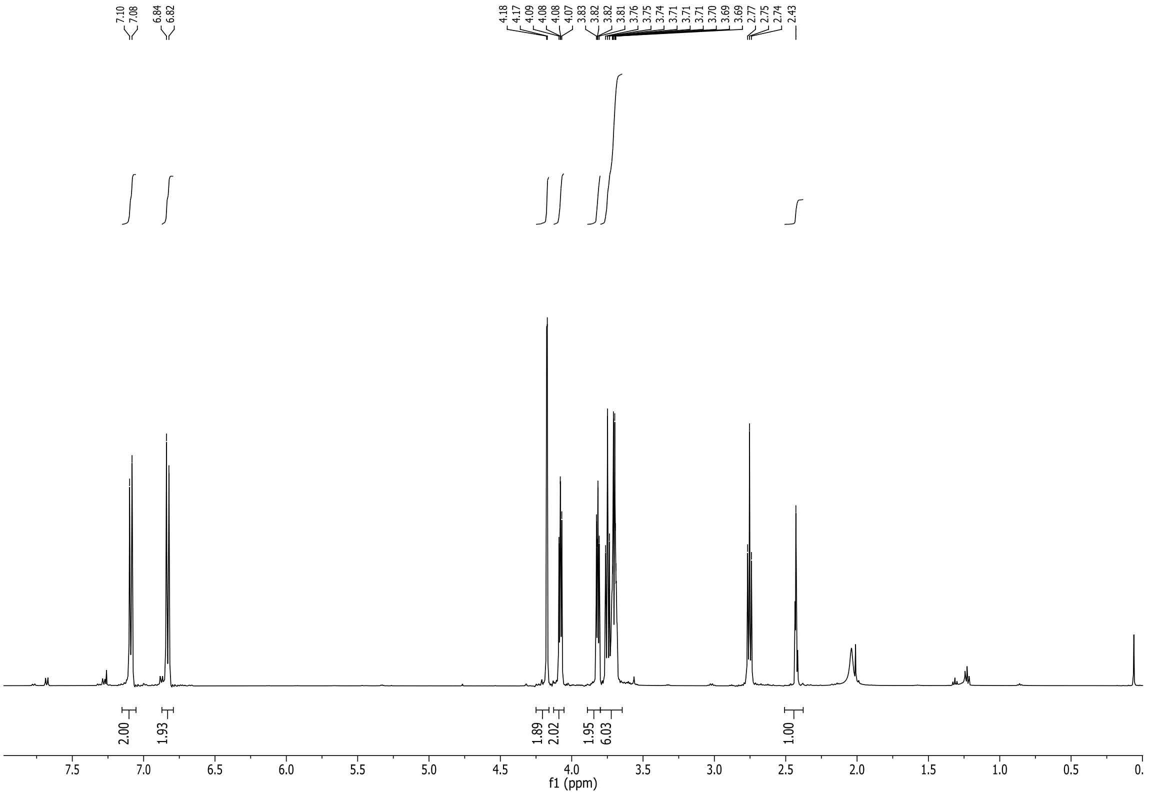

Figure S49. ^{1}**H NMR**

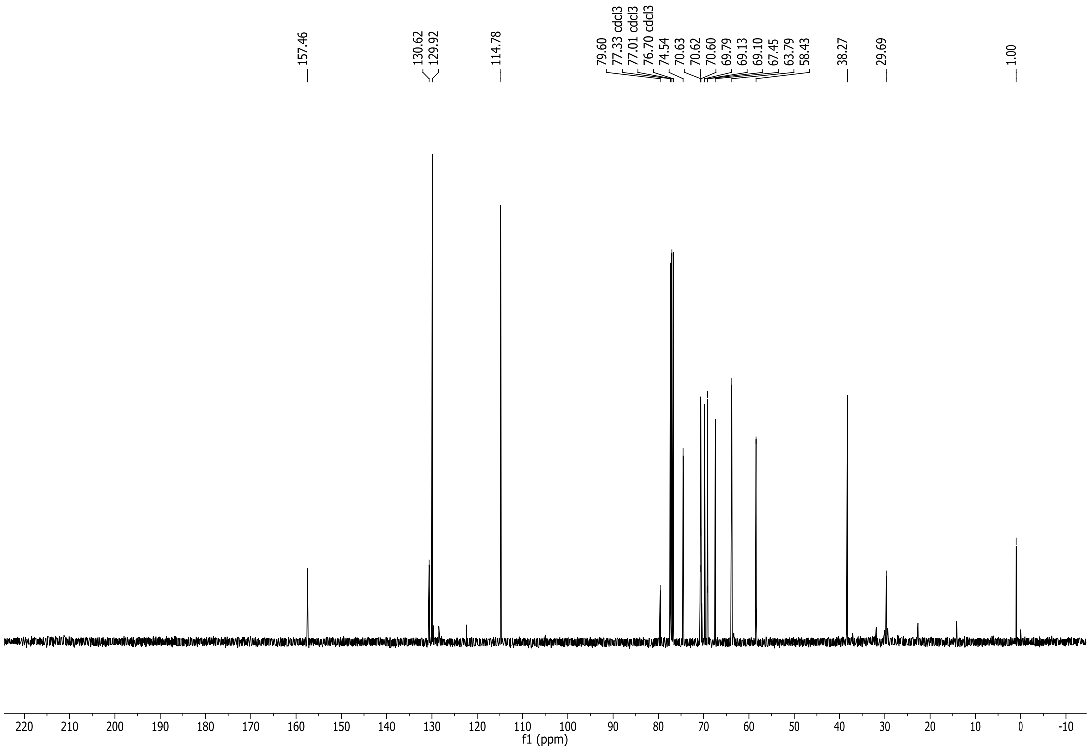

Figure S50. ^{13}C NMR

14.2. 4-(2-(prop-2-yn-1-yloxy)ethoxy)phenethyl 4-methoxylbenzensulfonate (20)

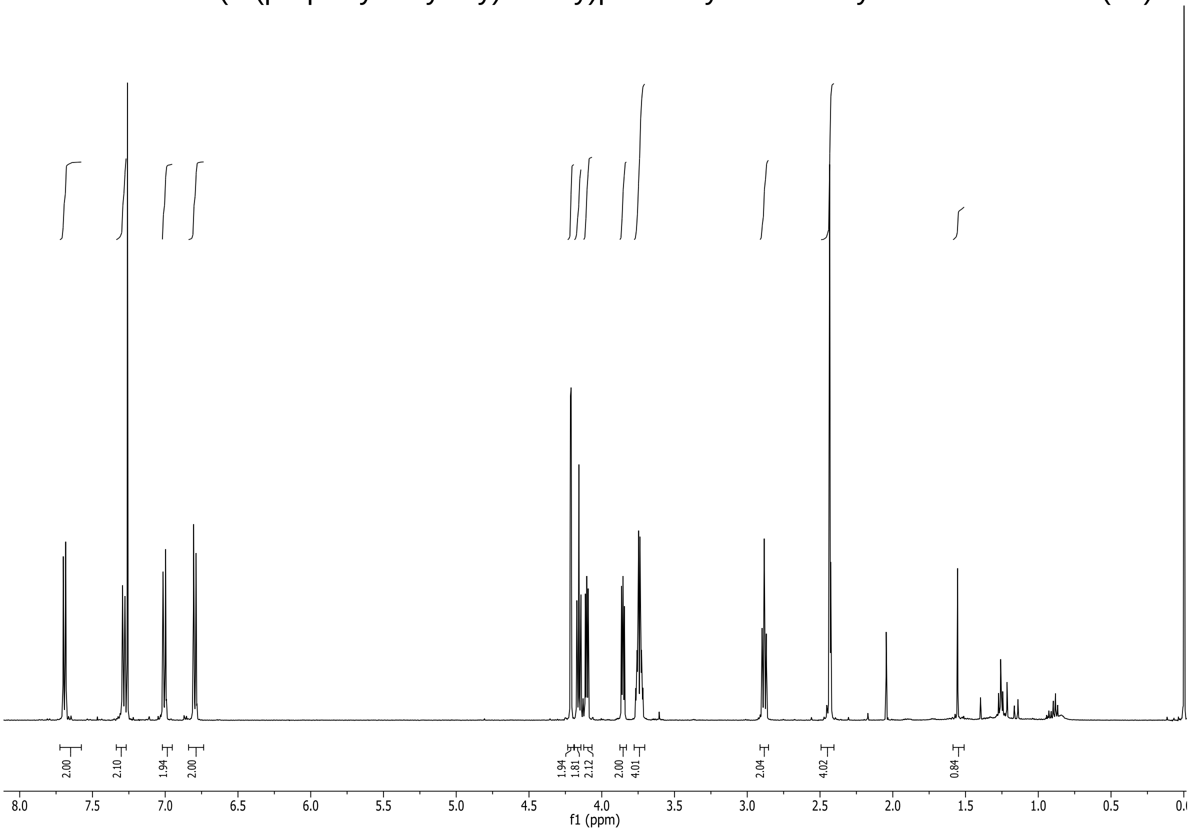

Figure S51. ^{1}H NMR

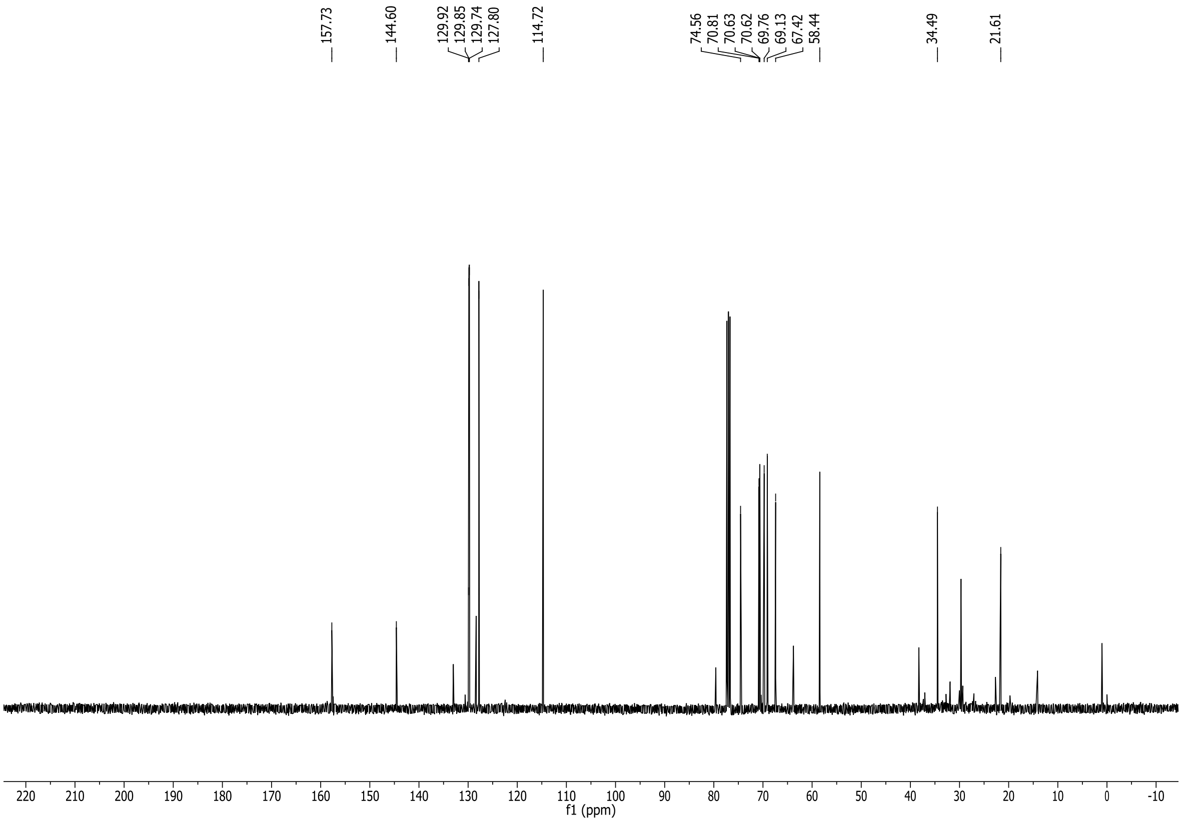

Figure S52. ^{13}**C NMR**

14.3. 1-(2-fluoroethyl)-4-(2-(pro-2-yn-1-yloxy)ethoxy)benzene (21)

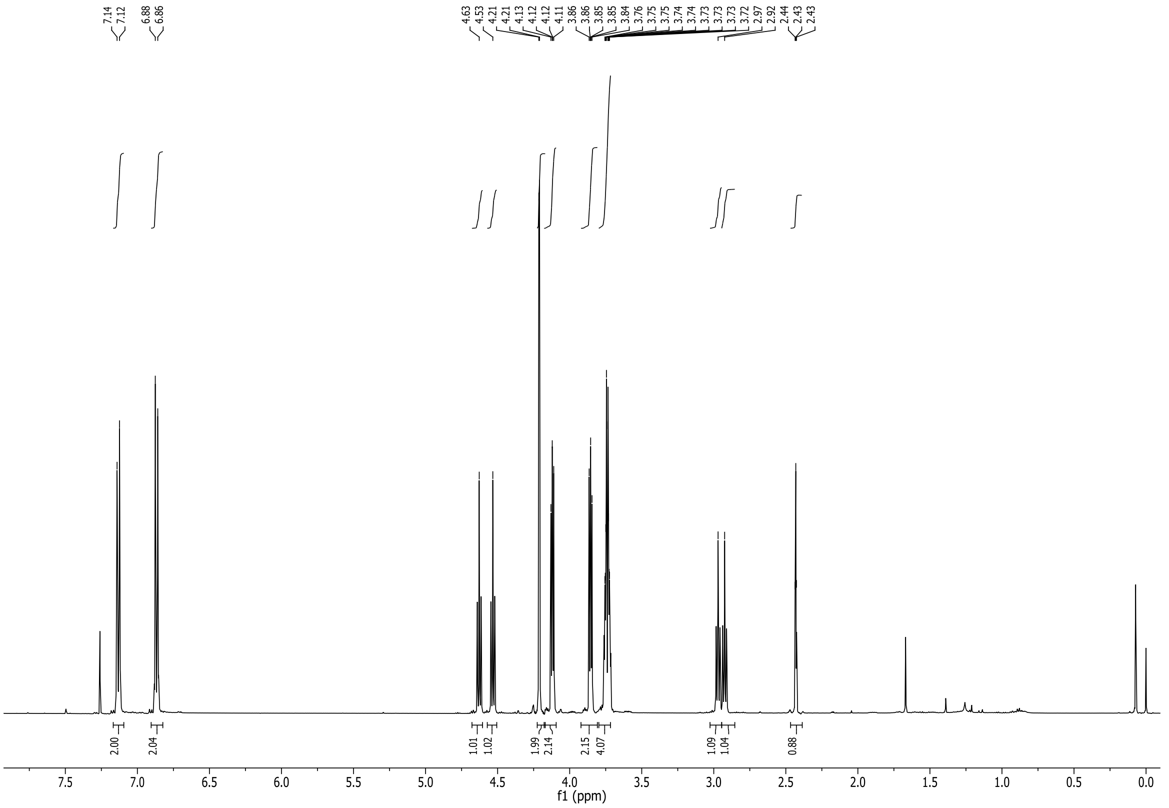

FigureS53. ^{1}**H NMR**

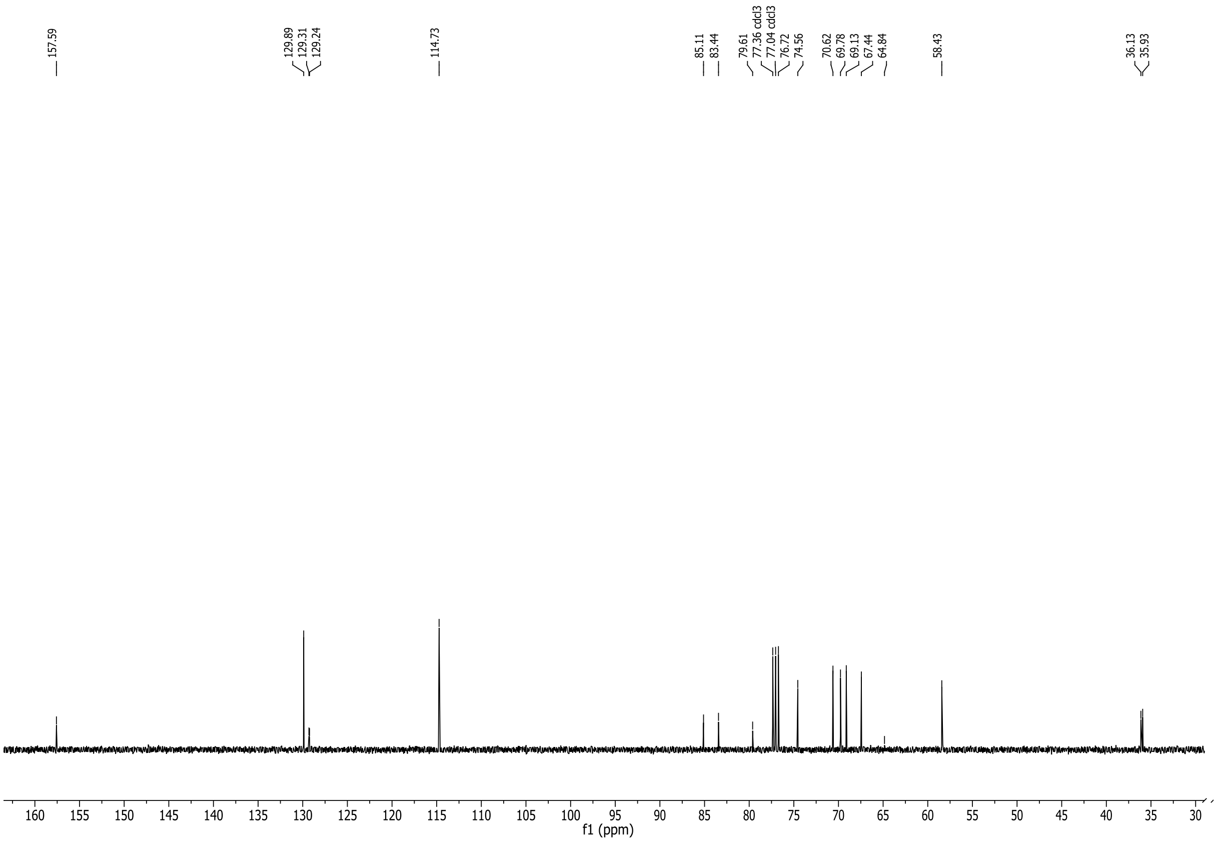

Figure S54. ^{11}**C NMR**

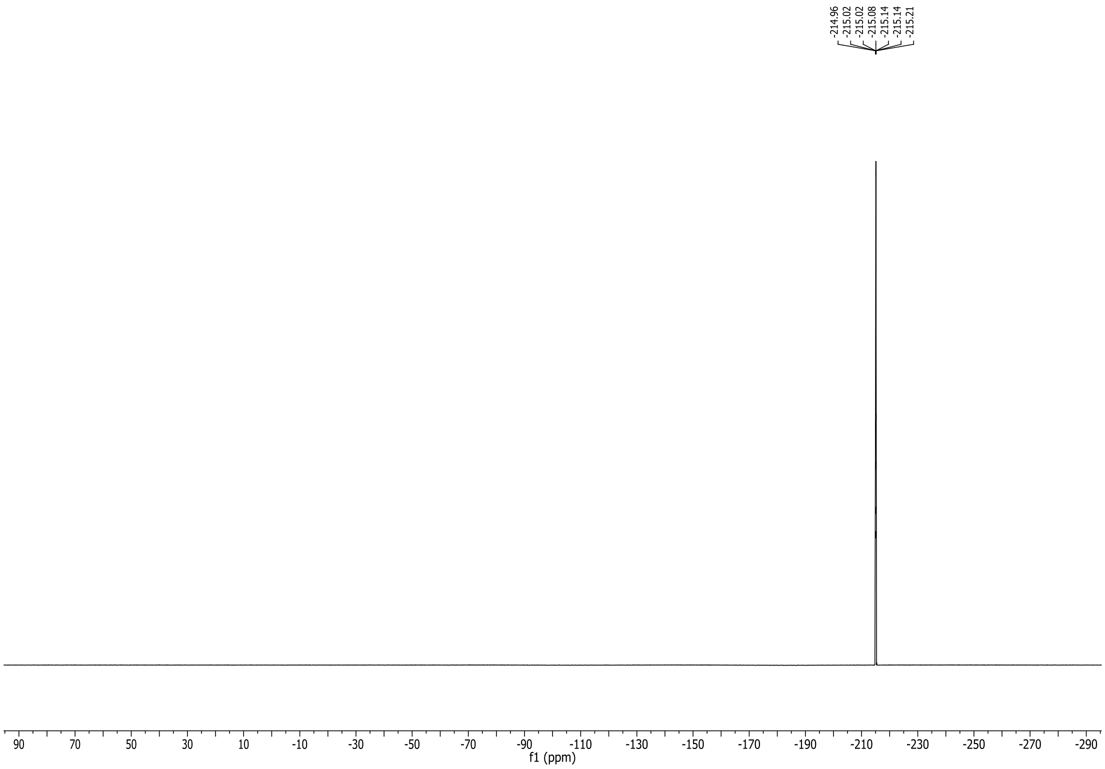

FigureS55. ^{19}**F NMR**

14.4. (((S)-1-carboxy-5-(4-((4-((2-(2-(4-(2-fluoroethyl)phenoxy)ethoxy)ethoxy)methyl)-1H-1,2,3-triazol-1-yl)methyl)benzamido)pentyl)carbamoyl)-L-glutamic acid (F-PSMA-MIC04)

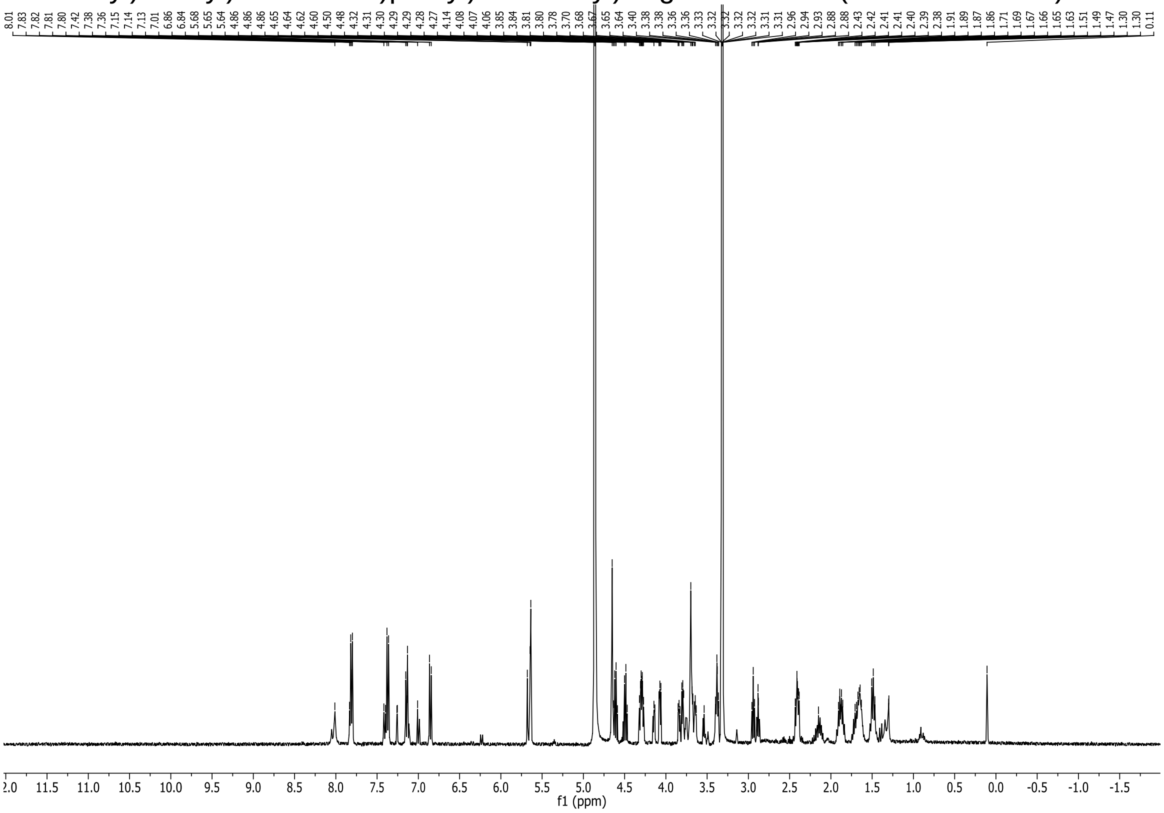

FigureS56. ^{1}H NMR

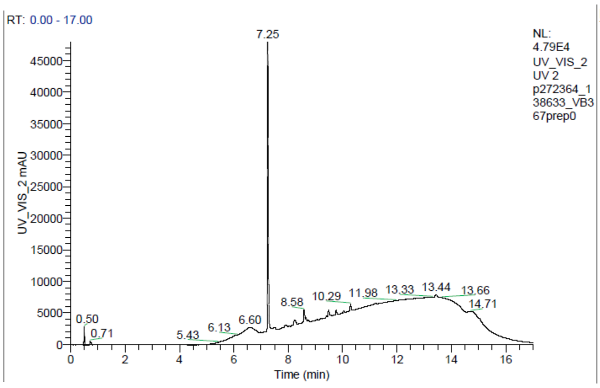

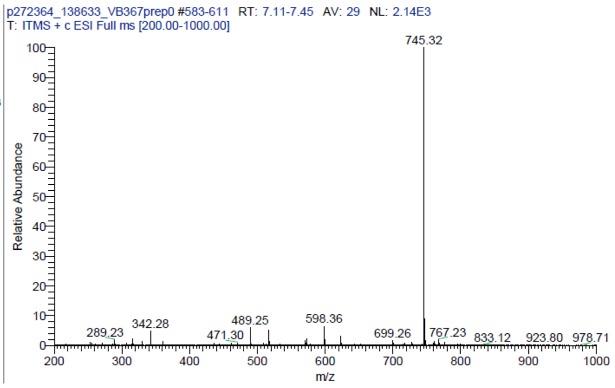

UPLC measurements were performed using the following setup: **Column:** ACQUITY UPLC® HSS T3 1.8µm, 2.1 x 150 mm; **Detection:** l = 254 nm; **Flow:** 0.3 mL/min; **Eluent A:** 0.1% formic acid in water; **Eluent B:** 0.1% formic acid in acetonitrile; **Program:** (0-1 min) 5% B; (1-8 min) linear gradient to 90% B; (8-11 min) 90% B; (11-12 min) linear gradient to 5% B; (12-17 min) 5% B.

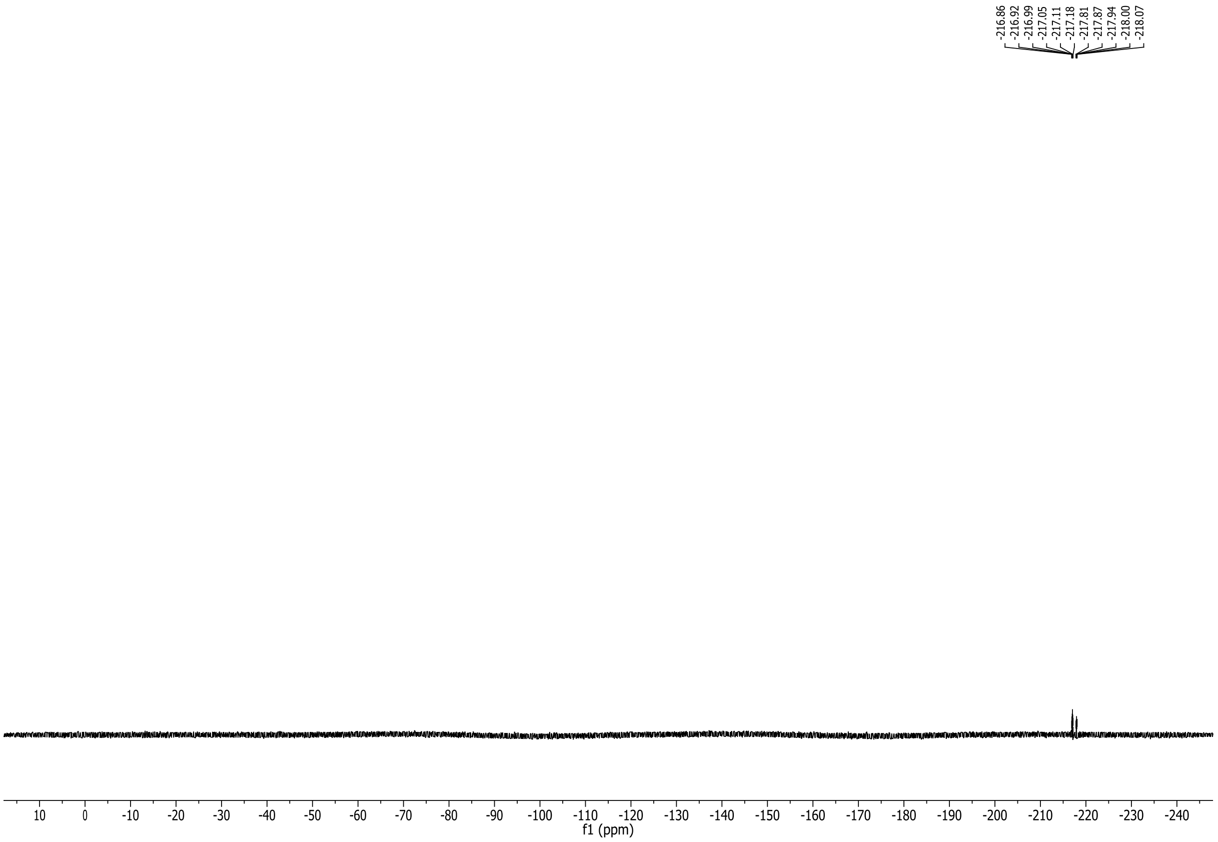

Figure S57. ^{19}F NMR
